# Anti‐tumoural activity of the G‐quadruplex ligand pyridostatin against BRCA1/2‐deficient tumours

**DOI:** 10.15252/emmm.202114501

**Published:** 2022-02-02

**Authors:** Florian J Groelly, Manuela Porru, Jutta Zimmer, Hugo Benainous, Yanti De Visser, Anastasiya A Kosova, Serena Di Vito, Violeta Serra, Anderson Ryan, Carlo Leonetti, Alejandra Bruna, Annamaria Biroccio, Madalena Tarsounas

**Affiliations:** ^1^ Genome Stability and Tumourigenesis Group, The MRC Oxford Institute for Radiation Oncology, Department of Oncology University of Oxford Oxford UK; ^2^ Area of Translational Research IRCCS Regina Elena National Cancer Institute Rome Italy; ^3^ Department of Ecological and Biological Sciences (DEB) University of Tuscia Viterbo Italy; ^4^ Experimental Therapeutics Group Vall d’Hebron Institute of Oncology Barcelona Spain; ^5^ Lung Cancer Translational Science Research Group, The MRC Oxford Institute for Radiation Oncology, Department of Oncology University of Oxford Oxford UK; ^6^ Molecular Pathology Division Centre for Cancer Evolution The Institute of Cancer Research London UK; ^7^ Present address: Medicines Discovery Catapult Cheshire UK

**Keywords:** BRCA1, BRCA2, DNA damage responses, G‐quadruplex ligands, pyridostatin, Cancer, DNA Replication, Recombination & Repair

## Abstract

The cells with compromised BRCA1 or BRCA2 (BRCA1/2) function accumulate stalled replication forks, which leads to replication‐associated DNA damage and genomic instability, a signature of *BRCA1/2*‐mutated tumours. Targeted therapies against *BRCA1/2*‐mutated tumours exploit this vulnerability by introducing additional DNA lesions. Because homologous recombination (HR) repair is abrogated in the absence of BRCA1 or BRCA2, these lesions are specifically lethal to tumour cells, but not to the healthy tissue. Ligands that bind and stabilise G‐quadruplexes (G4s) have recently emerged as a class of compounds that selectively eliminate the cells and tumours lacking BRCA1 or BRCA2. Pyridostatin is a small molecule that binds G4s and is specifically toxic to BRCA1/2‐deficient cells *in vitro*. However, its *in vivo* potential has not yet been evaluated. Here, we demonstrate that pyridostatin exhibits a high specific activity against BRCA1/2‐deficient tumours, including patient‐derived xenograft tumours that have acquired PARP inhibitor (PARPi) resistance. Mechanistically, we demonstrate that pyridostatin disrupts replication leading to DNA double‐stranded breaks (DSBs) that can be repaired in the absence of BRCA1/2 by canonical non‐homologous end joining (C‐NHEJ). Consistent with this, chemical inhibitors of DNA‐PKcs, a core component of C‐NHEJ kinase activity, act synergistically with pyridostatin in eliminating BRCA1/2‐deficient cells and tumours. Furthermore, we demonstrate that pyridostatin triggers cGAS/STING‐dependent innate immune responses when BRCA1 or BRCA2 is abrogated. Paclitaxel, a drug routinely used in cancer chemotherapy, potentiates the *in vivo* toxicity of pyridostatin. Overall, our results demonstrate that pyridostatin is a compound suitable for further therapeutic development, alone or in combination with paclitaxel and DNA‐PKcs inhibitors, for the benefit of cancer patients carrying *BRCA1/2* mutations.

## Graphical abstract

DNA replication and repair defects caused by loss of BRCA1/2 can be exploited therapeutically to specifically target tumours carrying mutations in these genes. Pyridostatin and its combinations with NU‐7441 and/or paclitaxel, specifically eliminate BRCA1/2‐deficient tumours *in vivo*.

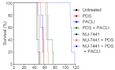

**The paper explained**

**Problem**

Mutations in *BRCA1* and *BRCA2* are frequently found in familial cancers, including breast, ovarian and prostate cancers, as well as in sporadic cancers. Exploiting the vulnerabilities of *BRCA1/2*‐mutated tumours with targeted therapies enables specific elimination of these tumours. However, resistance to both standard chemotherapeutic regimens and targeted therapies rapidly develops in patients. Therefore, there is an imperative need to identify novel drug candidates or treatment strategies to treat BRCA1/2‐deficient tumours.

**Results**

We report that the G‐quadruplex ligand pyridostatin specifically inflicts DNA damage and eliminates BRCA1/2‐deficient tumours *in vivo*, including PDTXs resistant to PARP inhibitors. We demonstrate that, in the absence of BRCA1/2, pyridostatin causes replication fork stalling and DNA double‐strand breaks, which can be repaired by C‐NHEJ reactions. Furthermore, we show that pyridostatin‐inflicted DNA damage leads to formation of cGAS‐associated micronuclei, which trigger innate immune responses. Finally, our study demonstrates that pyridostatin is well‐tolerated *in vivo* and that its combination with paclitaxel and the NU‐7441 DNA‐PKcs inhibitor exhibits long‐term anti‐cancer activity against BRCA1/2‐deficient tumours and substantially increases overall survival in mice.

**Impact**

Pyridostatin is a strong candidate drug for targeting BRCA1/2‐deficient tumours and for overcoming PARPi resistance *in vivo*. The combination of this compound with DNA‐PKcs inhibitors and paclitaxel could represent an effective treatment for the eradication of BRCA1/2‐mutated tumours. Additionally, our results suggest that pyridostatin may potentiate the efficacy of immune checkpoint inhibitors. Thus, pyridostatin has a clear potential for further clinical development.

## Introduction

BRCA1 and BRCA2 tumour suppressors have key roles in maintaining genome integrity (Zhao *et al*, 2019; Tarsounas & Sung, 2020). Firstly, they promote RAD51 loading onto RPA‐coated single‐stranded DNA (ssDNA) at sites of DSBs, thereby initiating DSB repair via HR reactions. Secondly, they act during DNA replication to protect stalled replication forks against nucleolytic degradation, thus preventing their conversion into detrimental DNA lesions. Thirdly, BRCA1 and BRCA2 promote the restart of stalled forks, thus facilitating genome replication. The replication roles of BRCA1 and BRCA2 are thought to be mediated by loading and/or stabilisation of RAD51 nucleoprotein filaments at sites of stalled forks (Bhat & Cortez, 2018). Consequently, BRCA1/2 inactivation is associated with accumulation of stalled forks, replication‐associated DNA damage and genome instability that can drive tumour formation.

Consistent with this, patients with familial breast, ovarian, prostate, pancreatic and other cancers often harbour heterozygous germline *BRCA1/2* mutations (Michl *et al*, 2016b). Moreover, somatic *BRCA1/2* mutations have recently been reported in sporadic cancers (Lal *et al*, 2019). Accumulation of replication‐associated DNA damage is characteristic of *BRCA1/2*‐mutated tumours and increases their vulnerability to targeted therapies, with inhibitors of poly(ADP‐ribose) polymerases (PARPs) as a prominent example (Lord & Ashworth, 2016). PARPis are specifically lethal to BRCA1/2‐deficient tumours because PARP1/2 enzymes, the targets of PARPi, bind to DNA ends. PARPis act by trapping PARP enzymes on DNA ends, leading to replication‐associated DNA damage when BRCA1/2 is abrogated (Murai *et al*, 2012; Pascal & Ellenberger, 2015). In addition, PARPis suppress the ability of PARP enzymes to PARylate their substrates, which is essential for the repair of ssDNA breaks that arise during DNA replication. More recently, PARPis have been shown to substantially potentiate the type I innate immune responses triggered by loss of BRCA1 or BRCA2 (Ding *et al*, 2018; Pantelidou *et al*, 2019; Reisländer *et al*, 2019). Thus, in addition to inflicting replication‐associated DNA damage and suppressing PARP1/2‐dependent DNA repair, PARPis act by enhancing the immunogenicity of BRCA1/2‐deficient tumours, thereby facilitating their elimination by the immune system.

In spite of tremendous efforts towards their optimisation, PARPis currently used in the clinic remain vulnerable to acquired drug resistance. *BRCA1*‐deleted tumours can acquire PARPi resistance via loss of 53BP1 (Bouwman *et al*, 2010; Bunting *et al*, 2010), REV7 (Xu *et al*, 2015) or the shieldin complex (Dev *et al*, 2018; Noordermeer *et al*, 2018; Tomida *et al*, 2018). In *BRCA2*‐deleted tumours, PARPi resistance can be triggered by loss of the poly(ADP‐ribose) glycohydrolase PARG (Gogola *et al*, 2018). Because PARPi resistance occurs frequently in cancer patients with *BRCA1/2* mutations (Gogola *et al*, 2019), there is a clinical need for new therapies, which can target resistant tumours.

Stalling of replication forks at physical barriers that obstruct their progression leads to replication‐associated pathologies, collectively known as replication stress (Zeman & Cimprich, 2014). Examples of replication barriers include DNA secondary structures (e.g. G4s), DNA repetitive elements (e.g. minisatellites, rDNA, telomeres) or sites of transcription‐replication conflicts (R‐loops; (Hamperl & Cimprich, 2016; Techer *et al*, 2017)). G4s are thought to form spontaneously on G‐rich ssDNA displaced during fork movement (Lipps & Rhodes, 2009). They consist of stacks of two or more G‐quartets formed by four guanines via Hoogsteen base pairing stabilised by a monovalent cation (Murat & Balasubramanian, 2014). More than 700,000 sequences with G4‐forming potential have been computationally identified in the human genome (Chambers *et al*, 2015). Given their thermodynamic stability, G4s can cause uncoupling of replisome components leading to fork stalling, which has the potential to trigger genomic instability. During physiological DNA replication, G4 are resolved by cellular helicases, thus enabling genome duplication (Tarsounas & Tijsterman, 2013). However, stabilisation of these DNA secondary structures by G4 ligands leads to persistent stalled replication forks and fork collapse, leading to replication‐associated DNA lesions. These accumulate specifically in the cells in which key DNA repair pathways (HR or non‐homologous end joining, NHEJ) are compromised (Zimmer *et al*, 2016; Xu *et al*, 2017).

Our previous work led to the discovery that G4 ligands (e.g. pyridostatin, PhenDC, RHPS4) are specifically toxic to HR‐compromised cells, including those that are BRCA1/2‐deficient (Zimmer *et al*, 2016). Importantly, we demonstrated that pyridostatin, the G4 ligand with the highest toxicity against the cells lacking BRCA1 or BRCA2, can also kill BRCA1‐deficient cells that acquired PARPi resistance (Zimmer *et al*, 2016). Subsequent studies led to the characterisation of a second G4 ligand, CX‐5461, which is also specifically toxic to BRCA1/2‐deficient cells and tumours (Xu *et al*, 2017). The CX‐5461 properties unravelled in this study made it a good candidate for further testing in a phase 1 clinical trial in cancer patients carrying DNA repair defects, including *BRCA1/2* mutations (https://clinicaltrials.gov/show/NCT02719977). Given that approximately 95% of the drugs tested in phase 1 trials fail to be certified for clinical use (Wong *et al*, 2019), we concentrated our efforts on characterisation of novel drugs, with mechanisms of action similar to CX‐5461, but with potentially superior pharmacological properties, which could therefore be more effective in the clinic. Moreover, a major cause of treatment failure in patients is development of drug resistance. Thus, we reasoned that a better understanding of the mechanism of action of such novel anti‐tumour drugs may enable the design of combination therapies for clinical use that could be more effective than individual drugs.

In this study, we characterise the *in vivo* potential of pyridostatin, a drug with well‐characterised toxicity against BRCA1/2‐deficient cells *in vitro*. We show that xenograft tumours lacking BRCA1 or BRCA2 are hypersensitive to pyridostatin and that the *in vivo* activity of this compound is similar to that of the PARPi talazoparib. Moreover, we demonstrate that pyridostatin shows anti‐tumour efficacy in BRCA1‐deficient, PARPi‐resistant tumour models, including patient‐derived xenografts. A key mechanism of action of pyridostatin is activation of cGAS/STING‐dependent innate immune responses, which may underlie its efficacy against BRCA1/2‐mutated tumours *in vivo*. We find that the DNA damage inflicted by pyridostatin can be repaired even in the absence of BRCA1 or BRCA2 by C‐NHEJ reactions and, consistent with this, we report synergistic effect between pyridostatin and the DNA‐PKcs inhibitor NU‐7441. A three‐drug combination consisting of pyridostatin, NU‐7441 and paclitaxel can effectively suppress growth or even eradicate BRCA1‐ or BRCA2‐deficient tumours and we propose that this triple combination represents an effective therapeutic strategy against *BRCA*‐mutated cancers. Altogether, our results suggest that the G4 ligand pyridostatin is a compound suitable for targeting BRCA1/2‐deficient tumours and for overcoming PARPi resistance *in vivo*, thus highlighting its potential for therapeutic development.

## Results

### Pyridostatin has anti‐tumoral activity against BRCA2‐deficient xenografts

**Figure 1:**
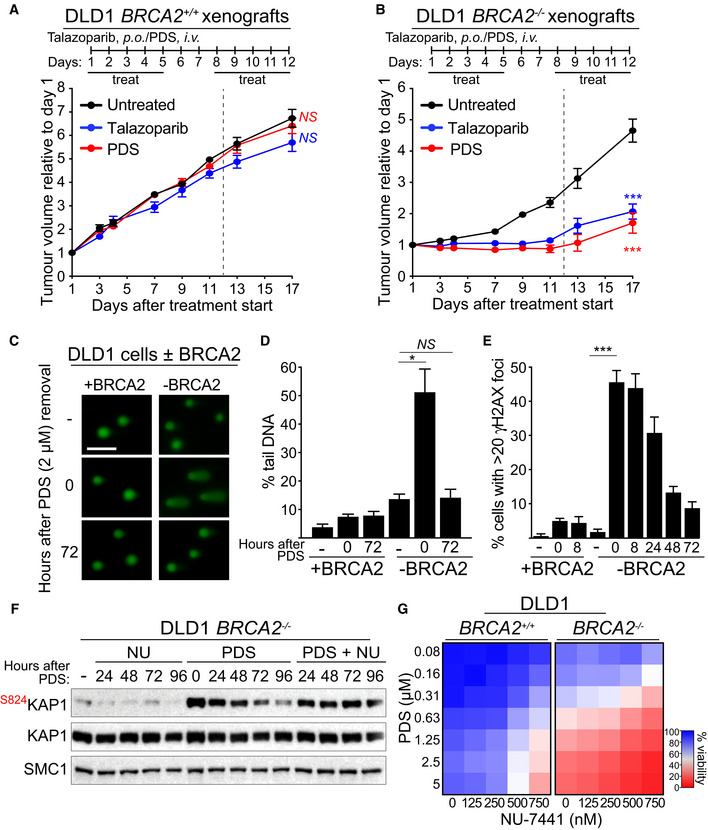
Pyridostatin inhibits growth of BRCA2‐deficient DLD1 xenograft tumours and inflicts repairable DNA damage in BRCA2‐deficient cells A, BCB17‐SCID male mice were injected intramuscularly into the hind leg muscles with (A) BRCA2‐proficient (*BRCA2* ^+/+^) or (B) BRCA2‐deficient (*BRCA2* ^−/−^) DLD1 cells. Pyridostatin (PDS) was administered intravenously (*i.v*.; 7.5 mg/kg/day) and talazoparib was administered orally (*p.o*.; 0.33 mg/kg/day), over the indicated periods of time. Vertical dashed line indicates end of treatment. Tumour volume was measured at the timepoints shown on the graph and expressed relative to tumour volume at the beginning of treatment. Each experimental group included *n* = 5 mice. Error bars represent SEM. *P* values were calculated between treated and untreated tumours at day 17, using an unpaired two‐tailed *t*‐test. ***P ≤ 0.001; *NS*, *P* > 0.05CDNA breaks were measured using alkaline comet assay in BRCA2‐proficient (+BRCA2) or ‐deficient (−BRCA2) human DLD1 cells treated with 2 µM of pyridostatin (PDS) for 16 h and released into fresh medium without pyridostatin. Representative images are shown. Scale bar represents 100 µm.DQuantification of DNA breaks shown in (C). Graph and error bars represent the mean and SEM of *n* = 3 independent experiments. A minimum of 50 cells were analysed per condition per experiment. *P* values were calculated using an unpaired two‐tailed *t*‐test. *P ≤ 0.05; *NS*, *P* > 0.05.EQuantification of γH2AX foci visualised using immunofluorescence staining in cells treated as in (C). A minimum of 200 cells were analysed per condition per experiment. Graph and error bars represent the mean and SEM of *n* = 3 independent experiments. *P* values were calculated using an unpaired two‐tailed *t*‐test. ***P ≤ 0.001; *NS*, *P* > 0.05.FBRCA2‐deficient (−BRCA2) human DLD1 cells were treated with 2 µM of pyridostatin (PDS) for 24 h. Next, pyridostatin was removed and cells were released in a medium containing 250 nM NU‐7441 (NU). Whole‐cell extracts were prepared at the indicated timepoints after release and immunoblotted as shown. SMC1 was used as a loading control. KAP1 phosphorylation site is indicated in red.GDose‐dependent viability assays of BRCA2‐proficient (+BRCA2) or ‐deficient (−BRCA2) human DLD1 cells treated with pyridostatin (PDS) and NU‐7441 at the indicated concentrations for 6 days. Graphs represent average values obtained from of *n* = 3 independent experiments, each performed in technical triplicates. Data information: Exact *P* values for (A‐B, D‐E) are provided in Appendix Table S8. Source data are available online for this figure.

Compounds that bind and stabilise G4s have been shown to be active against BRCA1/2‐deficient xenograft tumours established in mice (RHPS4 and CX‐5461). However, these have not yet been demonstrated to benefit patients with *BRCA* mutations. Moreover, *BRCA*‐mutated tumours are difficult to treat because they rapidly develop resistance to targeted therapies (e.g. PARP inhibitors; PARPi). Therefore, it is imperative to identify new G4 ligands that not only eliminate BRCA‐deficient tumours but also counteract resistant disease. Our previously published results (Zimmer *et al*, 2016) demonstrated that the G4 ligand pyridostatin is specifically toxic to BRCA2‐deficient cells *in vitro*. Here, we evaluated the potential of pyridostatin in eliminating BRCA2‐deficient xenograft tumours *in vivo*. To address this, we generated xenografts in CB17‐SCID mice using the isogenic *BRCA2* ^+/+^ (BRCA2‐proficient) and *BRCA2* ^−/−^ (BRCA2‐deficient) human colorectal adenocarcinoma DLD1 cells (Fig 1A and B). We extensively optimised conditions for *in vivo* use of pyridostatin and established that a dose schedule of 7.5 mg/kg/day administered intravenously for five consecutive days, followed by a 2‐day break and a second 5‐day treatment were well tolerated, as demonstrated by the lack of significant weight loss with no adverse clinical signs (Appendix Table S1). Using these conditions, we found that pyridostatin effectively and specifically inhibited growth of xenograft tumours established from BRCA2‐deficient DLD1 cells (Fig 1B). As a control, we used the PARPi talazoparib, known for its ability to eradicate BRCA1/2‐deficient tumours in mice (Shen *et al*, 2013) and recently licensed for use in metastatic breast cancer patients carrying *BRCA1/2* germline mutations (Litton *et al*, 2018). The anti‐tumoral effect of pyridostatin against the BRCA2‐deficient tumours was similar to talazoparib, and neither drug impaired the growth of BRCA2‐proficient tumours (Fig 1A; Appendix Table S1).

Furthermore, we investigated the *in vivo* response to pyridostatin using a second tumour model, established from isogenic *BRCA2* ^+/+^ and *BRCA2* ^−/−^ colorectal carcinoma HCT116 cells (Xu *et al*, 2014). Pyridostatin showed selective toxicity against BRCA2‐deficient HCT116 cell‐derived tumours (Appendix Fig S1A and B; Appendix Table S2), similarly to its effect in DLD1 cell‐derived xenografts.

Our previous work showed that pyridostatin treatment causes DNA damage accumulation in cells with compromised HR repair, including BRCA2‐deficient cells (Zimmer *et al*, 2016). Consistently, immunohistochemical (IHC) analyses revealed that BRCA2‐deficient, but not BRCA2‐proficient, tumours exhibited increased level of the DNA damage marker γH2AX upon exposure to either pyridostatin or talazoparib (Appendix Fig S1C–F). These results indicated that pyridostatin can specifically suppress not only the growth of the cells (Zimmer *et al*, 2016), but also of tumours lacking BRCA2 and that it acts *in vivo* by inflicting DNA damage.

### Pyridostatin induces DNA damage that is repaired by canonical non‐homologous end‐joining

In the course of our *in vivo* experiments using BRCA2‐deficient DLD1 and HCT116 cell‐derived xenografts we observed that, in spite of initial inhibition, tumours treated with pyridostatin resumed growth at the end of the treatment (Fig 1B; Appendix Fig S1B). This suggested that the DNA damage inflicted by pyridostatin, which underlies its toxicity against these tumours, can be repaired in the absence of BRCA2. We therefore attempted to gain further insight into the origins of the DNA damage induced by pyridostatin in BRCA2‐deficient cells and to identify potential DNA repair pathways that can promote its repair.

Aberrant replication is commonly associated with DNA damage accumulation (Zeman & Cimprich, 2014). Our previous work (Zimmer *et al*, 2016) demonstrated that treatment with pyridostatin slows down replication fork progression in BRCA2‐deficient cells, suggesting that G4s stabilised by pyridostatin assemble persistent DNA secondary structures that obstruct replication. BRCA2 has a central role in protecting stalled replication forks against nucleolytic degradation, illustrated by extensive shortening of nascent DNA when replication is arrested with hydroxyurea (HU) and BRCA2 is abrogated (Schlacher *et al*, 2011). We therefore investigated whether pyridostatin can cause fork stalling and degradation in BRCA2‐deficient cells, similarly to HU. To address this, we conducted DNA fibre assays, in which successive pulse‐labelling with CldU and IdU was followed by 5‐h HU or pyridostatin treatment (Appendix Fig S2A). The relative replication track length, expressed as the ratio of IdU to CldU tracks (Michl *et al*, 2016a), provided means to quantify fork stability. We found that, whilst neither HU nor pyridostatin had an effect on the relative track length in BRCA2‐proficient cells, both compounds led to significant attrition of the newly synthesised DNA in BRCA2‐deficient cells (Appendix Fig S2A). Addition of mirin, an MRE11 inhibitor, rescued this phenotype. These results suggested that pyridostatin‐stalled forks become substrates for MRE11‐dependent degradation in the absence of BRCA2.

We then assessed the consequences of replication fork instability in BRCA2‐deficient cells, by visualising the DNA breaks induced by pyridostatin and measuring their repair. Distinctly from previous studies, we investigated here whether the DNA damage inflicted by pyridostatin can be repaired in the cells lacking homologous recombination (i.e. BRCA1/2‐deficient) after removal of the drug and, if so, which repair pathways are required. To do this, we monitored either DSBs directly using established techniques (comet assay and mitotic chromosomes spreading) or DNA damage markers (i.e. γH2AX foci, ATM/ATR activation) in BRCA1/2‐deficient cells, during a recovery period of 3 days after the end of pyridostatin treatment. First, we used alkaline comet assays (Fig 1C and D) to quantify DNA damage by comparing the percentage of tail DNA relative to the total DNA in individual cells. We observed a significant increase in tail DNA, reflecting accumulation of DNA breaks, in BRCA2‐deficient but not BRCA2‐proficient DLD1 cells, upon treatment with 2 µM of pyridostatin for 16 h. Importantly, after releasing BRCA2‐deficient cells into fresh media for 72 h, the level of DNA breakage was reduced to that of untreated cells. Next, we visualised chromosome breaks and aberrations (e.g. radial chromosomes) using Giemsa‐stained spreads of metaphase chromosomes (Appendix Fig S2B and C). Treatment with pyridostatin caused an increase in broken/aberrant chromosomes in the cells lacking BRCA2, which were resolved 72 h after removing the compound from the media. These results indicated that pyridostatin‐induced DNA damage can be repaired in BRCA2‐independent manner after the treatment ended.

Our previous work demonstrated that pyridostatin inflicts DNA damage leading to ATM/ATR‐dependent checkpoint activation and G2/M arrest in the cells with compromised BRCA2 function (Zimmer *et al*, 2016). We therefore investigated whether pyridostatin‐induced ATM kinase activation is also attenuated in BRCA2‐deficient cells after removal of the compound. Treatment with pyridostatin triggered phosphorylation of KAP1 at Ser824 and phosphorylation of RPA at Ser4/Ser8 (Appendix Fig S3A), both well‐characterised ATM targets (Blackford & Jackson, 2017). Importantly, these modifications were diminished to the level of untreated cells within 72 h of release from pyridostatin treatment. In addition, γH2AX and 53BP1 foci, markers for DNA damage accumulation, showed a robust induction upon pyridostatin treatment specifically in the cells lacking BRCA2 (Fig 1E; Appendix Fig S3B and C), and gradually decreased after removal of the compound. Moreover, the ATM‐dependent G2/M arrest elicited by pyridostatin in BRCA2‐deficient cells (Zimmer *et al*, 2016), was also reversed by removal of pyridostatin from the media (Appendix Fig S3D), thus recapitulating the ATM signalling attenuation observed under the same conditions (Appendix Fig S3A).

Our results so far demonstrated that G4 stabilisation by pyridostatin inflicts DNA lesions and activates a potent DNA damage response (DDR) in BRCA2‐deficient cells, which, surprisingly, become gradually attenuated after pyridostatin removal from the media. This suggested that DNA repair reactions occur in BRCA2‐deficient cells, in spite of their compromised HR activity. We first investigated the involvement of alternative non‐homologous end joining (A‐NHEJ) in these repair events because tumours lacking BRCA1/2 have been reported to rely on this pathway for their survival (Ceccaldi *et al*, 2015). POLQ is an error‐prone polymerase central to A‐NHEJ repair (Yousefzadeh *et al*, 2014), where it facilitates microhomology annealing of ssDNA overhangs generated by end‐resection (Kent *et al*, 2015). Moreover, POLQ is required in *C. elegans* to prevent genomic instability stemming from endogenous G4s stabilised by genetic ablation of the DOG‐1/FANCJ helicase (Kruisselbrink *et al*, 2008; Castillo Bosch *et al*, 2014). We inhibited POLQ expression in human *BRCA2* ^+/+^ and *BRCA2* ^−/−^ DLD1 cells using siRNA (Appendix Fig S4A) and observed that, surprisingly, POLQ abrogation had no effect on the pyridostatin sensitivity of *BRCA2* ^−/−^ cells in clonogenic survival assays (Appendix Fig S4B). Consistent with this, we found that POLQ depletion did not affect either the levels of pyridostatin‐induced DNA damaged in *BRCA2* ^−/−^ cells, visualised using γH2AX or 53BP1 foci (Appendix Fig S4C and D), or their repair kinetics in recovery assays (Appendix Fig S4E and F). Taken together, these results indicate that POLQ is not required for the repair of pyridostatin‐induced DNA damage in these cells, during or after treatment with the compound.

We next investigated whether the C‐NHEJ pathway is implicated in the repair of pyridostatin‐induced DNA lesions in the absence of BRCA2. The DNA‐PKcs kinase is a core component of the C‐NHEJ pathway, which orchestrates ligation of DNA ends by bringing them in close proximity and recruiting the DNA ligase IV/XRCC4 complex to complete the repair reaction (Calsou *et al*, 2003). We treated *BRCA2* ^−/−^ cells with pyridostatin for 16 h, then released them in media containing the DNA‐PKcs chemical inhibitor NU‐7441 for 4 days (Fig 1F). Under these conditions, pyridostatin‐induced KAP1 phosphorylation at Ser824, a marker of ATM activation, persisted for 96 h after pyridostatin was replaced with NU‐7441 in the media. This suggested that pyridostatin‐induced DNA damage is repaired by C‐NHEJ in *BRCA2* ^−/−^ cells. As a control for the specificity of NU‐7441, we tested its effect of on the viability of HAP1 cells carrying a deletion of the *PRKDC* gene, which encodes DNA‐PKcs (Appendix Fig S5A and B). No significant difference in cell viability was detected between *PRKDC* wild type (*PRKDC* WT) and *PRKDC‐*deleted (*PRKDC* KO) cells upon treatment with NU‐7441 (Appendix Fig S5A and B) suggesting lack of significant off‐target effects, although the inhibitor showed some toxicity against *PRKDC* KO cells (Appendix Fig S5B). Human cells lacking DNA‐PKcs were reported to be sensitive to pyridostatin (Xu *et al*, 2017). We recapitulated these results using *PRKDC‐*deleted HAP1 cells (Appendix Fig S5C). Importantly, the combination of pyridostatin and NU‐7441 showed no additional toxicity to *PRKDC* ^−/−^ HAP1 cells relative to pyridostatin alone, further validating the specificity of this inhibitor for DNA‐PKcs (Appendix Fig S5D).

Given that DNA‐PKcs is required for the repair of pyridostatin‐induced lesions in the absence of BRCA2, we next tested the effect of pyridostatin/NU‐7441 combination on *BRCA2* ^+/+^ and *BRCA2* ^−/−^ DLD1 cell survival using viability assays (Fig 1G). We observed that addition of NU‐7441 potentiated the toxicity of pyridostatin against cells lacking BRCA2, consistent with a synergistic effect of the two compounds in this background. Taken together, these results established that the repair of DNA damage induced by pyridostatin in the absence of BRCA2 is dependent on the C‐NHEJ and does not require the A‐NHEJ repair pathway.

### Pyridostatin treatment triggers cGAS/STING‐dependent innate immune responses in BRCA2‐deficient cells

We and others have shown that BRCA2‐deficient cells and tumours activate innate immune responses orchestrated by the cGAS/STING pathway, as a result of spontaneous DNA damage accumulation (Ding *et al*, 2018; Chabanon *et al*, 2019; Pantelidou *et al*, 2019; Reisländer *et al*, 2019). These immune responses caused by chronic loss of BRCA1 or BRCA2 function are potentiated by PARPi treatment, consistent with the ability of these drugs to increase endogenous DNA damage levels in the cells lacking BRCA2 (Ding *et al*, 2018; Pantelidou *et al*, 2019; Reisländer *et al*, 2019). Because pyridostatin also inflicts DNA damage in BRCA2‐deficient cells (Fig 1C–E; Appendix Fig S2B and C; Appendix Fig S3), we investigated whether this G4 ligand can also trigger immune responses.

**Figure 2:**
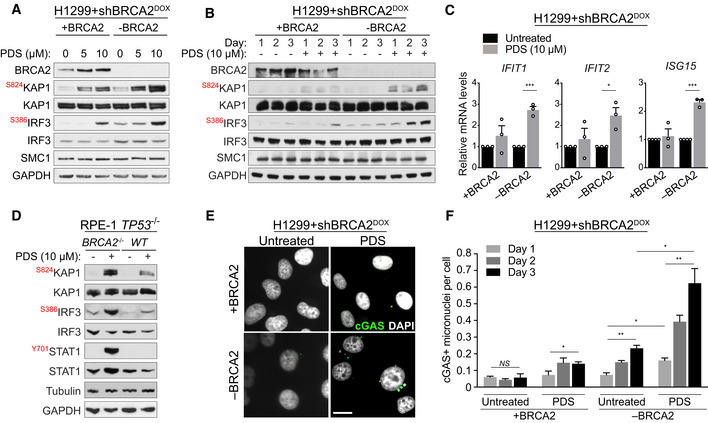
Pyridostatin activates innate immune responses in BRCA2‐deficient cells H1299+shBRCA2^DOX^ cells were grown for 4 days in the presence (‐BRCA2) or absence (+BRCA2) of DOX and subsequently treated with 5 or 10 μM pyridostatin (PDS) for 3 days. Whole‐cell extracts were immunoblotted as indicated. KAP1 and IRF3 phosphorylation sites are shown in red. SMC1 and GAPDH were used as loading controls.H1299+shBRCA2^DOX^ cells were grown for 4 days in the presence or absence of DOX and subsequently treated with 10 of μM pyridostatin (PDS) for 1, 2 or 3 days. Whole‐cell extracts were immunoblotted as indicated. KAP1 and IRF3 phosphorylation sites are shown in red. SMC1 and GAPDH were used as loading controls.Quantitative RT‐PCR of cells grown as in (A) and treated with 10 µM of pyridostatin (PDS) for 3 days was performed using primers specific for the indicated genes. mRNA levels are expressed relative to GAPDH and to untreated cells. Error bars represent the SEM of *n* = 3 independent experiments, each performed in technical triplicate. *P* values were calculated using an unpaired two‐tailed *t*‐test. *P ≤ 0.05; ***P ≤ 0.001.
*BRCA2* ^+/+^ and *BRCA2* ^−/−^ RPE‐1 cells were treated with 10 μM of pyridostatin (PDS) for 2 days. Whole‐cell extracts were immunoblotted as indicated. KAP1, IRF3 and STAT1 phosphorylation sites are shown in red. Tubulin and GAPDH were used as loading controls.H1299+shBRCA2^DOX^ cells treated as in (B) were fixed and prepared for immunofluorescence with antibody against cGAS. DNA was counterstained with DAPI. Scale bar represents 20 µm.Quantification of cGAS‐positive micronuclei per cells shown in (E). Graph and error bars represent the mean and SEM of *n* = 3 independent experiments. A minimum of 250 cells were analysed per condition per experiment. *P* values were calculated using an unpaired two‐tailed *t*‐test. *P ≤ 0.05; **P ≤ 0.01; *NS*, *P* > 0.05. Data information: Exact *P* values for (C, F) are provided in Appendix Table S8. Source data are available online for this figure.

To address this, we cultured H1299 human cells carrying a DOX‐inducible *BRCA2* shRNA cassette in the presence or absence of pyridostatin for 3 days (Fig 2A). Immunoblotting demonstrated not only effective suppression of BRCA2 expression by DOX treatment, but also dose‐dependent activation of ATM signalling by pyridostatin, illustrated by increased KAP1 phosphorylation at Ser824. The latter was also detected in BRCA2‐proficient cells, possibly as a consequence of DNA lesions caused by the long exposure (3 days) to a relatively high concentration of pyridostatin (10 µM). Importantly, we observed that treatment of BRCA2‐deficient cells with pyridostatin induced phosphorylation of IRF3 at Ser386, indicative of its nuclear translocation (Lin *et al*, 1998) and cGAS/STING pathway activation (Ishikawa *et al*, 2009; Tanaka & Chen, 2012; Sun *et al*, 2013). We concluded that pyridostatin triggers innate immune responses in BRCA2‐deficient cells, as a consequence of DNA damage accumulation. To strengthen this conclusion, we performed time course experiments in which we monitored activation of ATM signalling and cGAS/STING pathway in time (Fig 2B). Our results indicate a clear increase in the levels of KAP1 Ser824 and IRF3 Ser386 phosphorylation in BRCA2‐deficient H1299 cells with duration of pyridostatin treatment.

Activation of the cGAS/STING axis also triggers interferon signalling, detectable as enhanced transcription of interferon stimulated genes (ISGs; Reisländer *et al*, 2020). Consistent with this, quantitative RT‐PCR revealed a substantial increase in the mRNA levels of ISGs (*IFIT1*, *IFIT2*, *ISG15*) specifically in pyridostatin‐treated BRCA2‐deficient cells (Fig 2C).

Next, we generated *BRCA2* ^−/−^ human RPE‐1 cells using CRISPR/Cas9‐mediated gene deletion, as an additional cellular model for testing the impact of pyridostatin on the cells lacking BRCA2. Loss of BRCA2 protein expression in two different *BRCA2* ^−/−^ RPE‐1 clones was demonstrated using immunoblotting and sensitivity to the PARPi olaparib (Appendix Fig S6A). Moreover, *BRCA2* ^+/+^, but not *BRCA2* ^−/−^, RPE‐1 cells accumulated RAD51 nuclear foci upon exposure to ionising radiation (Appendix Fig S6B), further confirming abrogation of BRCA2 function. Treatment of *BRCA2* ^−/−^ RPE‐1 cells with 10 µM of pyridostatin for 48 h induced robust KAP1 Ser824 and IRF3 Ser386 phosphorylation (Fig 2D), indicative of DNA damage accumulation and innate immune response activation, respectively. In these cells, pyridostatin also induced phosphorylation of STAT1 at Tyr701 (Fig 2D), a marker of activated interferon signalling (Shuai *et al*, 1993).

Genomic instability triggers micronuclei formation, likely as a result of chromosome mis‐segregation during mitosis. Recognition of micronuclei by the cytosolic DNA sensor cGAS activates the cGAS/STING pathway and downstream innate immune responses. We monitored the frequency of cGAS‐associated micronuclei in the cells treated with pyridostatin (Fig 2E) and observed a time‐dependent increase in BRCA2‐deficient cells relative to the wild‐type counterparts (Fig 2F). These results demonstrate that innate immunity in BRCA2‐deficient cells stems from DNA damage and genomic instability elicited by pyridostatin.

### Pyridostatin overcomes PARPi resistance in *Brca1‐*deleted cells and tumour models

**Figure 3:**
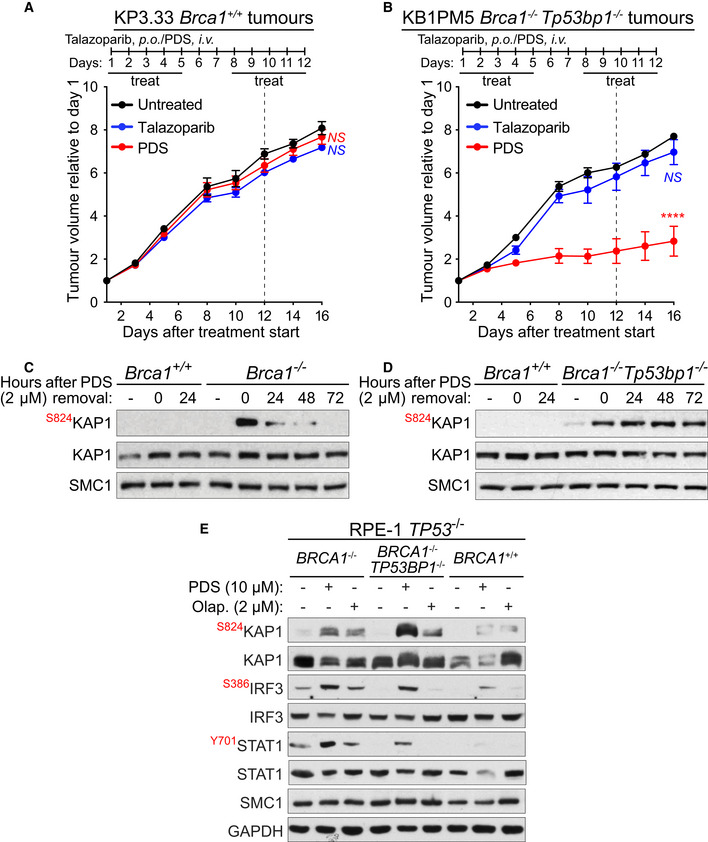
Pyridostatin impairs growth of PARPi‐resistant BRCA1‐deficient tumours and triggers DNA damage and innate immune responses in BRCA1‐deficient PARPi‐resistant cells A, BFVB female mice were injected intramuscularly with (A) BRCA1‐proficient KP3.33 (*Brca*1^+^/^+^) or (B) BRCA1/53BP1‐deficient KB1PM5 (*Brca*1^−/−^, *Tp53bp1* ^−/−^ *)* mouse mammary tumour cells. Pyridostatin (PDS) was administered intravenously (*i.v*.; 7.5 mg/kg/day) and talazoparib was administered orally (*p.o*.; 0.33 mg/kg/day), over the indicated periods of time. Vertical dotted line indicates end of treatment. Tumour volume was measured at the timepoints shown on the graph and expressed relative to tumour volume at the beginning of treatment. Each experimental group included *n* = 5 mice. Error bars represent SEM. *P* values were calculated between treated and untreated tumours at day 16, using an unpaired two‐tailed *t*‐test. ****P ≤ 0.0001; *NS*, *P* > 0.05.C, DBRCA1‐proficient KP3.33 (*Brca1* ^+/+^), BRCA1‐deficient KB1PM5 (*Brca1* ^−/−^) and BRCA1/53BP1‐deficient KB1PM5 (*Brca1* ^−/−^/*Tp53bp1* ^−/−^) mouse mammary tumour cells were treated with 2 µM of pyridostatin (PDS) for 24 h and released into fresh medium without pyridostatin. Whole‐cell extracts were prepared 0 to 72 h after release and immunoblotted as indicated. SMC1 was used as a loading control. KAP1 phosphorylation site is indicated in red.E
*BRCA1* ^+/+^, *BRCA1* ^−/−^ and *BRCA1* ^−/−^/*TP53BP1* ^−/−^ RPE‐1 cells were treated with 10 μM of pyridostatin (PDS) or 2 μM of olaparib for 2 days. Whole‐cell extracts were immunoblotted as indicated. KAP1, IRF3 and STAT1 phosphorylation sites are shown in red. SMC1 and GAPDH were used as loading controls. Data information: Exact *P* values for (A, B) are provided in Appendix Table S8. Source data are available online for this figure.

The clinical efficacy of PARPi against BRCA1/2‐deficient tumours is limited by the rapid development of drug resistance (Gourley *et al*, 2019). Therefore, concerted efforts are currently focused on the development of therapeutical strategies that eliminate resistant disease. Our previously published work (Zimmer *et al*, 2016) demonstrated that *Brca1* ^−/−^ mouse cells that acquired PARPi resistance via loss of 53BP1 can be targeted by pyridostatin. Here, we tested the ability of this compound to inhibit growth of *Brca1* ^−/−^ *Tp53bp1* ^−/−^ xenograft tumours established in mice (Fig 3A and B). We found that treatment with pyridostatin effectively suppressed tumour growth, in contrast with the PARPi talazoparib (Fig 3B; Table 1). Neither drug had an effect on the growth of *Brca1* ^+/+^ tumours (Fig 3A).

**Table 1:** *In vivo* anti‐tumour efficacy of pyridostatin and talazoparib on *Brca1* ^+/+^ and *Brca1* ^−/−^ *Tp53bp1* ^−/−^ allografts.

Treatment	Tumour volume inhibition (%)	Tumour growth delay (days)	Stable disease	Body weight loss (%)	Toxic deaths
*Brca1* ^+/+^ Pyridostatin	9	0	0/5	0	0/5
*Brca1* ^−/−^ *Tp53bp1* ^−/−^ Pyridostatin	65	10	1/5	0	0/5
*Brca1* ^+/+^ Talazoparib	11	0	0/5	0	0/5
*Brca1* ^−/−^ *Tp53bp1* ^−/−^ Talazoparib	22	0	0/5	0	0/5

FVB female mice were injected intramuscularly with 4 × 10^6^ cells per mouse. Tumours were allowed grow to approximately 250 mm^3^ before initiation of treatment (day 1). Mice were treated with pyridostatin (*i.v*.; 7.5 mg/kg/day) and talazoparib (*p.o*.; 0.33 mg/kg/day) for five consecutive days, followed by 2‐day break and five more days of treatment. Each experimental group included *n* = 5 mice. Tumour volume inhibition was calculated at the nadir of the effect using the formula: (1 ‐ [tumour volume in treated mice] / [tumour volume in untreated mice]) ×100 and expressed as average for *n* = 5 mice in each group. Tumour growth delay was calculated as the median time in days required for untreated and treated tumours to reach 700 mm^3^. Stable disease was defined as mice in which tumour volume did not change for at least 2 weeks after initiation of treatment. Body weight loss is reported as weight at the end of treatment relative to the first day of treatment (%), as average for *n* = 5 mice in each group.

Having established that pyridostatin triggers DNA damage signalling in BRCA2‐deficient cells, which is silenced after compound removal from the media (Appendix Fig S3A), we now tested whether *Brca1* ^−/−^ and *Brca1* ^−/−^ *Tp53bp1* ^−/−^ show a similar repair capacity. To address this, we monitored KAP1 phosphorylation at Ser824, as a marker for DNA damage‐induced ATM activation, in the cells treated with pyridostatin and released in media without the compound. Pyridostatin did not trigger ATM activation in *Brca1* wild‐type cells, but inflicted DNA damage in *Brca1* ^−/−^ cells, which was repaired upon release from treatment (Fig 3C). In contrast, in *Brca1* ^−/−^ *Tp53bp1* ^−/−^ cells pyridostatin‐induced ATM activation persisted after compound removal (Fig 3D), indicating that pyridostatin inflicts unrepairable DNA damage in these cells.

To gain an understanding of the mechanism that prevents the repair of pyridostatin‐induced DNA damage in the absence of BRCA1 and 53BP1, we conducted cell fractionation experiments. The cells were treated with pyridostatin for 24 h, allowed to recover for 72 h after removal of the compound, followed by cell fractionation (Appendix Fig S7A). Tubulin was used as a marker for the soluble fraction, and SMC1 as a marker for the chromatin‐bound fraction. We observed that XRCC4, a central component of the C‐NHEJ pathway, was not detectable in *Brca1* ^−/−^ *Tp53bp1* ^−/−^ cells, in contrast to *Brca1* ^+/+^ or *Brca1* ^−/−^ cells, either in the chromatin fraction or the whole cell extract (Appendix Fig S7A and B). These results suggest that the inability of *Brca1* ^−/−^ *Tp53bp1* ^−/−^ to repair the DNA damage induced by pyridostatin is due to loss of a key C‐NHEJ activity (i.e. XRCC4).

Next, we addressed whether pyridostatin can trigger innate immune responses in PARPi‐resistant cells. In these experiments, we used human RPE‐1 cells carrying *BRCA1* gene deletion (*BRCA1* ^−/−^; Zimmermann *et al*, 2018). Treatment of these cells with 10 µM of pyridostatin induced DSBs signalling, as shown by KAP1 Ser824 phosphorylation (Fig 3E). Similar results were obtained upon treatment with 2 µM of olaparib, used as a control. Both IRF3 Ser386 and STAT1 Tyr701 phosphorylation were induced by pyridostatin, and to a lesser extent by olaparib, in the *BRCA1* ^−/−^ cells. These results suggested that pyridostatin triggers innate immune responses associated with DNA damage accumulation in the cells lacking BRCA1 (Fig 3E), similarly to BRCA2‐deficient cells (Fig 2A, B and D). We next tested the effect of the two drugs in BRCA1/53BP1‐deficient RPE‐1 cells (*BRCA1* ^−/−^ *TP53BP1* ^−/−^), which are resistant to PARPi (Dev *et al*, 2018). Strikingly, phosphorylation of IRF3 Ser386 and STAT1 Tyr701 were increased solely after pyridostatin treatment in these cells (Fig 3E). Consistently, pyridostatin caused higher levels of DNA damage compared with olaparib, as indicated by KAP1 Ser824 phosphorylation. Taken together, these results demonstrate that pyridostatin triggers cGAS/STING‐dependent immune responses in BRCA1/2‐deficient cells, including those that have acquired PARPi resistance, and that these responses correlate with the pyridostatin ability to inflict ATM‐activating DNA damage.

### PARPi‐resistant BRCA1‐deficient patient‐derived xenograft tumours are targeted by pyridostatin

**Figure 4:**
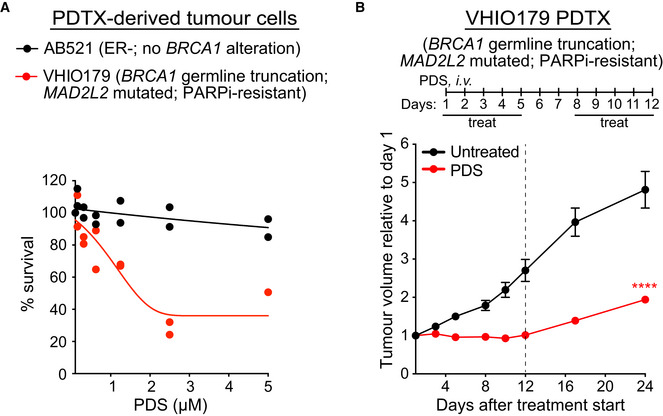
Pyridostatin inhibits growth of BRCA1‐deficient human PDTCs and PDTXs Dose‐dependent viability assays of PDTCs derived from breast cancer samples (Bruna *et al*, 2016) treated with pyridostatin (PDS) at the indicated doses. Values from *n* = 2 independent experiments are shown. AB521: ER‐negative tumour, no *BRCA1* alteration; VHIO179: tumour with *BRCA1* germline mutation and *MAD2L2* inactivating mutation (olaparib‐resistant).VHIO179 PDTXs were grafted into CB17‐SCID female mice. Pyridostatin was administered intravenously (7.5 mg/kg/day) over the indicated periods of time. Vertical dotted line indicates end of treatment. Tumour volume was measured at the timepoints shown on the graph and expressed relative to tumour volume at the beginning of treatment. Each experimental group included *n* = 7 mice. Error bars represent SEM. *P* values were calculated between treated and untreated tumours at day 24, using an unpaired two‐tailed *t*‐test. ****P ≤ 0.0001. Data information: Exact *P* values for (B) are provided in Appendix Table S8.

To further confirm the sensitivity of PARPi‐resistant tumours to pyridostatin, we used *ex vivo* cultures of patient‐derived tumour xenograft cells (PDTCs; Fig 4A), known to recapitulate tumour vulnerability to specific drugs (Bruna *et al*, 2016). BRCA1‐proficient PDTCs (AB521) showed no growth defects when cultured in the presence of pyridostatin. In contrast, VHIO179, a tumour carrying *BRCA1* germline truncation and previously shown to be resistant to treatment with PARPi due to an inactivating mutation in the *MAD2L2 (REV7)* gene (Bruna *et al*, 2016; Cruz *et al*, 2018), was hypersensitive to pyridostatin. To investigate whether this response was recapitulated in the tumour context, we grafted the VHIO179 patient‐derived tumour xenografts (PDTX) into CB17‐SCID female mice and treated them with pyridostatin. This treatment effectively inhibited tumour growth relative to untreated tumours (Fig 4B; Table 2). Overall, these results demonstrated that *in vivo* pyridostatin has an inhibitory effect against *BRCA1*‐mutated patient xenograft tumours that developed PARPi resistance.

**Table 2:** *In vivo* anti‐tumour efficacy of pyridostatin on BRCA1‐deficient human PDTXs.

Treatment	Tumour volume inhibition (%)	Tumour growth delay (days)	Stable disease	Body weight loss (%)	Toxic deaths
Pyridostatin	60	16	6/7	0	0/7

CB17‐SCID female mice were implanted with small tumour fragments derived from the VHIO179 PDTXs. Tumours were allowed grow to approximately 200 mm^3^ before initiation of treatment (day 1). Mice were treated with pyridostatin (*i.v*.; 7.5 mg/kg/day) for five consecutive days, followed by 2‐day break and five more days of treatment. Each experimental group included *n* = 7 mice. Tumour volume inhibition was calculated at the nadir of the effect using the formula: (1 ‐ [tumour volume in treated mice] / [tumour volume in untreated mice]) ×100 and expressed as average for *n* = 7 mice in each group. Tumour growth delay was calculated as the median time in days required for untreated and treated tumours to reach 400 mm^3^. Stable disease was defined as mice in which tumour volume did not change for at least 2 weeks after initiation of treatment. Body weight loss is reported as weight at the end of treatment relative to the first day of treatment (%), as average for *n* = 7 mice in each group.

### Anti‐tumour activity of drug combinations containing pyridostatin against BRCA‐deficient xenografts

**Figure 5:**
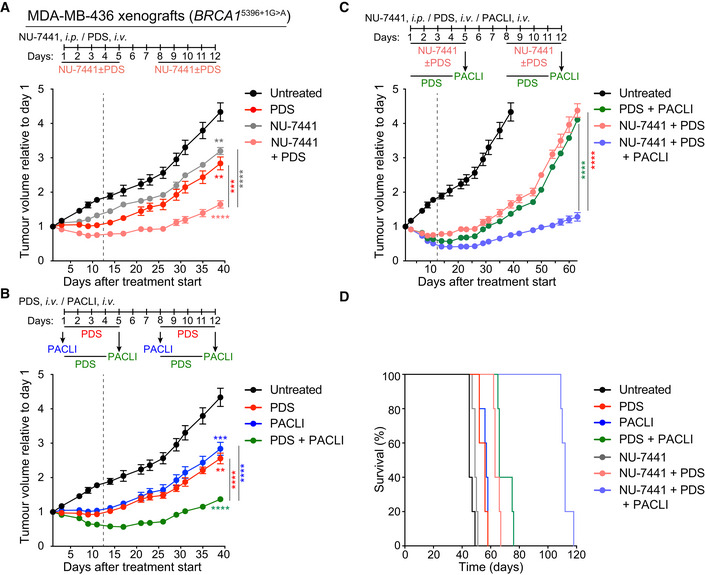
Antitumoral effect of pyridostatin, NU‐7441 and paclitaxel combinations in BRCA1‐deficient xenograft tumours BRCA1‐deficient MDA‐MB‐436 human cells were injected intramuscularly in SCID female mice. Pyridostatin (PDS) was administered intravenously (*i.v*.; 7.5 mg/kg/day) and NU‐7441 was administered intraperitoneally (*i.p*.; 10 mg/kg/day), over the indicated periods of time. Vertical dashed line indicates end of treatment. Tumour volume was measured at the timepoints shown on the graph and expressed relative to tumour volume at the beginning of treatment. Each experimental group included *n* = 5 mice. Error bars represent SEM. *P* values were calculated at day 39 using an unpaired two‐tailed *t*‐test, between treated and untreated tumours, or between the NU‐7441+PDS combination and each single treatment. **P ≤ 0.01; ***P ≤ 0.001; ****P ≤ 0.0001.Pyridostatin (PDS) was administered intravenously (*i.v*.; 7.5 mg/kg/day) over the indicated periods of time, and paclitaxel (PACLI) was administered intravenously (*i.v*.; 20 mg/kg/day) on indicated single days. Vertical dashed line indicates end of treatment. Tumour volume was measured at the timepoints shown on the graph and expressed relative to tumour volume at the beginning of treatment. Each experimental group included *n* = 5 mice. Error bars represent SEM. *P* values were calculated at day 39 using an unpaired two‐tailed *t*‐test, between treated and untreated tumours, or between the PDS+PACLI combination and each single treatment. **P ≤ 0.01; ***P ≤ 0.001; ****P ≤ 0.0001.Pyridostatin (PDS) was administered intravenously (*i.v*.; 7.5 mg/kg/day), NU‐7441 was administered intraperitoneally (*i.p*.; 10 mg/kg/day), over the indicated periods of time, and paclitaxel (PACLI) was administered intravenously (*i.v*.; 20 mg/kg/day) on indicated single days. Vertical dashed line indicates end of treatment. Tumour volume was measured at the timepoints shown on the graph and expressed relative to tumour volume at the beginning of treatment. Each experimental group included *n* = 5 mice. Error bars represent SEM. *P* values were calculated at day 63 using an unpaired two‐tailed *t*‐test between the NU‐7441+PDS+PACLI combination and each double treatment. ****P ≤ 0.0001.Kaplan‐Meier survival curve for mice treated as in (C), with each treatment group containing *n* = 5 mice. Statistical significance was assessed by Log‐rank test (****P < 0.0001). Data information: Exact *P* values for (A‐C) are provided in Appendix Table S8 and (D) in Appendix Table S3.

Our *in vitro* results (Fig 1C–E; Appendix Figs S2B and S3A–D) demonstrated that the DNA damage inflicted by pyridostatin in BRCA2‐deficient cells is repaired after removal of the drug, suggesting that the toxicity of this compound is reversible and tumour growth may recover in the long term. Therefore, we next attempted to identify drug combinations that prevent attenuation of the pyridostatin response and/or increase its *in vivo* efficacy. To address this, we established xenograft tumours in CB17‐SCID mice using BRCA1‐compromised MDA‐MB‐436 mammary breast cancer cells (Fig 5), which carry an inactivating 5,396+1G>A mutation in *BRCA1* gene (Elstrodt *et al*, 2006), and monitored their long‐term (up to 63 days) response to pyridostatin alone, or in combination with other drugs.

We focused on the combination between pyridostatin and the DNA‐PKcs inhibitor NU‐7441, given the synergy between the two drugs observed *in vitro* against BRCA2‐deficient cells (Fig 1G). Additionally, we sought to evaluate pyridostatin in combination with a drug routinely used in the clinic. We chose paclitaxel, because it is a chemotherapeutic drug frequently used to treat breast and ovarian cancer. Although its precise anti‐tumoral activity is not fully elucidated (Mitchison *et al*, 2017), paclitaxel is known to interfere with mitosis by stabilising microtubules and to induce apoptosis in mitosis‐arrested cells via cGAS activation (Zierhut *et al*, 2019). When tested *in vitro*, the combination of pyridostatin with NU‐7441 or with paclitaxel, strongly reduced the ability of MDA‐MB‐436 cells to form colonies, whilst cells treated with the triple combination did not survive (Appendix Fig S7C and D).

Consistent with the *in vitro* results, the combinations of pyridostatin with NU‐7441 or with paclitaxel were effective *in vivo* against MDA‐MB‐436 xenografts (Fig 5A and B). Treatment with each drug showed an initial anti‐tumour effect, followed by recovery after administration of the compound had ended. Although the combination of pyridostatin with NU‐7441 or paclitaxel were more potent than each drug alone and caused stable disease in all treated mice (Table 3), tumour growth resumed approximately 30 days after initiation of treatment (Fig 5A and B).

**Table 3:** *In vivo* anti‐tumour efficacy of pyridostatin, NU‐7441, paclitaxel and their combination on MDA‐MB‐436 xenografts.

Treatment	Tumour volume inhibition (%)	Stable disease	Partial response	Complete response	Increase in survival (%)^a^	Body weight loss (%)	Toxic deaths
NU‐7441	26	0/5	0/5	0/5	9	3	0/5
Pyridostatin	42	1/5	0/5	0/5	24	7	0/5
Paclitaxel	46	3/5	0/5	0/5	27	9	0/5
Pyridostatin + NU‐7441	58	5/5	0/5	0/5	40	8	0/5
Pyridostatin + Paclitaxel	71	5/5	0/5	0/5	47	7	0/5
Pyridostatin + NU‐7441 + Paclitaxel	82	0/5	4/5	1/5	149	8	0/5

**Figure 6:**
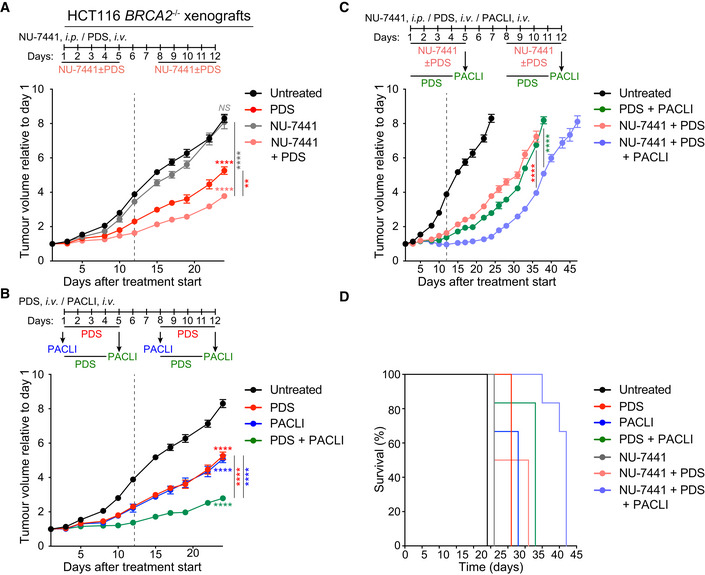
Antitumoral effect of pyridostatin, NU‐7441 and paclitaxel combinations in BRCA2‐deficient HCT116 xenograft models BRCA2‐deficient HCT116 human cells were injected intramuscularly in SCID male mice. Pyridostatin (PDS) was administered intravenously (*i.v*.; 7.5 mg/kg/day) and NU‐7441 was administered intraperitoneally (*i.p*.; 10 mg/kg/day), over the indicated periods of time. Vertical dashed line indicates end of treatment. Tumour volume was measured at the timepoints shown on the graph and expressed relative to tumour volume at the beginning of treatment. Each experimental group included *n* = 6 mice. Error bars represent SEM. *P* values were calculated at day 24 using an unpaired two‐tailed *t*‐test *NS*, *P* > 0.05; **P ≤ 0.01; ****P ≤ 0.0001.Pyridostatin (PDS) was administered intravenously (*i.v*.; 7.5 mg/kg/day) over the indicated periods of time, and paclitaxel (PACLI) was administered intravenously (*i.v*.; 20 mg/kg/day) on indicated single days. Vertical dashed line indicates end of treatment. Tumour volume was measured at the timepoints shown on the graph and expressed relative to tumour volume at the beginning of treatment. Each experimental group included *n* = 6 mice. Error bars represent SEM. *P* values were calculated at day 24 using an unpaired two‐tailed *t*‐test ****, *P* ≤ 0.0001.Pyridostatin (PDS) was administered intravenously (*i.v*.; 7.5 mg/kg/day), NU‐7441 was administered intraperitoneally (*i.p*.; 10 mg/kg/day), over the indicated periods of time, and paclitaxel (PACLI) was administered intravenously (*i.v*.; 20 mg/kg/day) on indicated single days. Vertical dashed line indicates end of treatment. Tumour volume was measured at the timepoints shown on the graph and expressed relative to tumour volume at the beginning of treatment. Each experimental group included *n* = 6 mice. Error bars represent SEM. *P* values were calculated at day 36 or 38 using an unpaired two‐tailed *t*‐test ****, *P* ≤ 0.0001.Kaplan‐Meier survival curve for mice treated as in (C), with each treatment group containing *n* = 6 mice. Statistical significance was assessed by Log‐rank test (****P < 0.0001). Data information: Exact *P* values for (A‐C) are provided in Appendix Table S8 and (D) in Appendix Table S6.

Tumours were allowed to grow for 6 days to approximately 220 mm^3^ before initiation of treatment (day 1). Mice were treated with pyridostatin (*i.v*.; 7.5 mg/kg/day) at days 1–5 and 8–12 and NU‐7441 (*i.p*.; 10 mg/kg/day) at days 1–5 and 8–12, 2 h before injection of pyridostatin. Paclitaxel (*i.v*.; 20 mg/kg) was administered at day 5 and 12, for the double and triple combinations (Fig 6C). Each experimental group included *n* = 5 mice. Tumour volume inhibition was calculated at the nadir of the effect using the formula: (1 ‐ [tumour volume in treated mice] / [tumour volume in untreated mice]) ×100 and expressed as average for *n* = 5 mice in each group. Stable disease was defined as mice in which tumour volume did not change for at least 2 weeks after initiation of treatment. Partial or complete responses were defined as mice in which ≥ 50% reduction of tumour volume or tumour disappearance, respectively, were observed for at least 2 weeks after initiation of treatment. Increase in survival was calculated by comparing median survival of treated relative to untreated mice (%). Body weight loss is reported as weight at the end of treatment relative to the first day of treatment (%), as average for *n* = 5 mice in each group.

^a^ Animals were euthanized for ethical reasons when tumours reached a mean of 1.2–1.5 cm^3^ in volume.

Given that the pyridostatin/NU‐7441 and pyridostatin/paclitaxel combinations were well‐tolerated in mice (Table 3) and that combination of the three drugs virtually abrogated survival of MDA‐MB‐436 *in vitro* (Appendix Fig S7C and D), we next established conditions for treating BRCA1‐deficient xenograft tumours with the pyridostatin/NU‐7441/paclitaxel combination. We found that treatment with this triple combination led to a persistent anti‐tumour response up to 63 days after drug administration was initiated (Fig 5C, Table 3). Although each drug or two‐drug combinations significantly increased mouse survival relative to untreated animals, the combination of the three drugs led to a substantial increase in therapeutic efficacy. Indeed, we observed 100% survival for approximately 115 days after initiation of treatment (Fig 5D; Appendix Table S3). The survival advantage conferred by the pyridostatin/NU‐7441/paclitaxel combination correlated with its long‐term anti‐tumoral activity observed *in vivo*.

To determine whether the pyridostatin/NU‐7441/paclitaxel combination specifically suppresses growth of BRCA‐deficient, but not BRCA‐proficient tumours, we established xenograft tumours using *BRCA2* ^+/+^ and *BRCA2* ^−/−^ HCT116 human cells. Consistent with *in vitro* results (Appendix Fig S7C and D), neither pyridostatin nor its combination with NU‐7441 and/or paclitaxel suppressed growth of *BRCA2* ^+/+^ tumours (Appendix Fig S8A–C, Appendix Table S4) or had an impact on mouse survival (Appendix Fig S8D, Appendix Table S5). In contrast, the combination of pyridostatin with NU‐7441 or paclitaxel showed an anti‐tumoral effect superior to each single drug against *BRCA2* ^−/−^ HCT116 tumours, although they did not prevent tumour growth in the long term (Fig 6A–C; Table 4). The triple combination of pyridostatin/NU‐7441/paclitaxel effectively inhibited tumour growth for approximately 10 days following the end of the treatment (Fig 6C; Table 4) and resulted in 100% survival for 40 days after initiation of the treatment (Fig 6D; Appendix Table S6). We concluded that pyridostatin in combination with the DNA‐PKcs inhibitor NU‐7441 and paclitaxel has a specific and potent anti‐BRCA1/2‐deficient tumour activity superior to each compound alone, and to the combination of pyridostatin with each single compound, thereby supporting the potential of this triple combination to be exploited therapeutically in *BRCA1/2*‐mutated cancer patients.

**Table 4:** *In vivo* anti‐tumour efficacy of pyridostatin, NU‐7441, paclitaxel and their combination on HCT116 BRCA2^−/−^ xenografts.

Treatment	Tumour volume inhibition (%)	Stable disease	Partial response	Complete response	Increase in survival (%)^a^	Body weight loss (%)	Toxic deaths
NU‐7441	18	0/6	0/6	0/6	8	2	0/6
Pyridostatin	43	0/6	0/6	0/6	29	3	0/6
Paclitaxel	45	0/6	0/6	0/6	38	5	0/6
Pyridostatin + NU‐7441	57	0/6	0/6	0/6	29	4	0/6
Pyridostatin + Paclitaxel	67	3/6	0/6	0/6	58	7	0/6
Pyridostatin + NU‐7441 + Paclitaxel	81	6/6	0/6	0/6	96	8	0/6

Tumours were allowed to grow for 6 days to approximately 220 mm^3^ before initiation of treatment (day 1). Mice were treated with pyridostatin (*i.v*.; 7.5 mg/kg/day) at days 1–5 and 8–12 and NU‐7441 (*i.p*.; 10 mg/kg/day) at days 1–5 and 8–12, 2 h before injection of pyridostatin. Paclitaxel (*i.v*.; 20 mg/kg) was administered at days 1 and 8 and at days 5 and 12, for the double and triple combinations (Appendix Fig S8). Each experimental group included *n* = 6 mice. Tumour volume inhibition was calculated at the nadir of the effect using the formula: (1 ‐ [tumour volume in treated mice]/[tumour volume in untreated mice]) x100 and expressed as average for *n* = 6 mice in each group. Stable disease was defined as mice in which tumour volume did not change for at least 2 weeks after initiation of treatment. Partial or complete responses were defined as mice in which ≥ 50% reduction of tumour volume or tumour disappearance, respectively, were observed for at least 2 weeks after initiation of treatment. Increase in survival was calculated by comparing median survival of treated relative to untreated mice (%). Body weight loss is reported as weight at the end of treatment relative to the first day of treatment (%), as average for *n* = 5 mice in each group.

^a^ Animals were euthanised for ethical reasons when tumours reached a mean of 1.7–2 cm^3^ in volume.

## Discussion

In this study, we report that pyridostatin is a compound suitable for *in vivo* use and has high specificity against BRCA1/2‐deficient tumours. These results are important for the following reasons: (i) since its discovery as a compound toxic to cancer cells *in vitro* (Rodriguez *et al*, 2008), pyridostatin was suspected to be toxic in mice and therefore unsuitable for *in vivo* studies. Pyridostatin toxicity was attributed to its ability to alter transcription and to inflict DNA lesions associated with failed replication (Rodriguez *et al*, 2012). Here, we demonstrate that pyridostatin is well‐tolerated in mice and has robust anti‐tumoral activity, when administered intravenously; (ii) pyridostatin inhibits growth of BRCA1‐ and BRCA2‐deficient tumours in mice, thus its previously demonstrated *in vitro* activity (Zimmer *et al*, 2016) can be recapitulated *in vivo*; (iii) pyridostatin inhibits growth of *BRCA1*‐deleted patient‐derived xenografts that have acquired PARPi resistance, thus providing a means to target this difficult to eliminate tumour subset; (iv) mechanistically, we demonstrate that, in the absence of BRCA1 or BRCA2, pyridostatin disrupts DNA replication and inflicts replication‐associated DNA damage that can be repaired by C‐NHEJ. In addition, pyridostatin triggers a type I innate immune response in the cells lacking BRCA1/2, which may potentiate its anti‐tumoral activity; (v) pyridostatin combination with the DNA‐PKcs inhibitor NU‐7441 and paclitaxel shows long‐term efficacy against BRCA1‐deficient tumours. Thus, pyridostatin‐based drug combinations may represent a viable strategy for the treatment of *BRCA1/2*‐mutated cancer patients.

Pyridostatin was initially designed and characterised as a small molecule that can bind telomeric G4s (Rodriguez *et al*, 2008) and was subsequently demonstrated to bind and stabilise G4s genome‐wide (Rodriguez *et al*, 2012). As a consequence, pyridostatin interferes with transcription and replication, and inflicts replication‐associated DSBs. More recently, it was proposed that pyridostatin may promote TOP2 trapping on the DNA, in addition to G4 stabilisation, thus generating multiple types of cytotoxic replication barriers (Olivieri *et al*, 2020). Importantly, we discovered that pyridostatin is selectively toxic to HR‐deficient cells and can specifically kill BRCA1/2‐deficient cells *in vitro* (Zimmer *et al*, 2016). It was subsequently shown that the ability to eliminate BRCA1/2‐deficient cells and tumours is also a feature of the G4 ligand CX‐5461 (Xu *et al*, 2017), which is currently evaluated in clinical trials in patients with DNA repair defects (NCT02719977 ClinicalTrials.gov). CX‐5461 was originally identified as an inhibitor of Pol I‐dependent rRNA synthesis (Drygin *et al*, 2011), with the ability to activate p53‐dependent apoptosis (Bywater *et al*, 2012). Because it interferes with rDNA transcription, thus altering the translation capacity of the cell, CX‐5461 shows significant cytotoxicity, even at low concentrations. The results of the first phase I clinical trial for dose escalation study indicates dose‐dependent and ‐independent adverse effects in patients (Khot *et al*, 2019).

Due to its potent anti‐proliferative effects against a panel of cancer cell lines (Rodriguez *et al*, 2012), it had been anticipated that pyridostatin is unsuitable for *in vivo* experiments. We have performed extensive tolerability studies in mice and established a schedule for pyridostatin administration (Fig 1A and B; Appendix Table S1), that enabled us to test *in vivo* its anti‐tumour potential. Our results demonstrate that pyridostatin effectively inhibits growth of BRCA2‐deficient xenograft tumours, thus recapitulating our *in vitro* results. Moreover, pyridostatin inflicted DNA damage in tumours, similarly to PARPi, which provides a mechanistic basis for its tumour‐killing potential. Our results using CX‐5461 and pyridostatin in mice at the maximum tolerated dose for each drug, showed that pyridostatin has a higher therapeutic efficacy against BRCA1/2‐deficient tumours, when compared to CX‐5461 (Appendix Table S7).

Pyridostatin is a compound with extensively characterised mechanism of action. Earlier studies showed that it inflicts DSBs at sites of G4 inferred computationally (Rodriguez *et al*, 2012) and that these DSBs are specifically lethal when BRCA2 is abrogated (Zimmer *et al*, 2016). Here, we demonstrate that DNA damage induced by pyridostatin in the absence of BRCA2 does not exclusively depend on the ability of this compound to bind telomeric DNA, but stems from replication defects caused by fork stalling at sites throughout the genome, where pyridostatin binds to DNA. In the present work, we provide further mechanistic detail into the replication pathologies inflicted by pyridostatin in BRCA2‐deficient cells. We show that replication forks arrested by treatment with pyridostatin are degraded by the MRE11 nuclease, conceivably leading to lethal DSB accumulation. This represents a key novel aspect relative to previous studies (Zimmer *et al*, 2016; Xu *et al*, 2017), which suggested diminished replication rate as a result of G4 stabilisation, as a possible origin of the DSBs induced by pyridostatin or CX‐5461, respectively.

Importantly, in the current work, we are not simply monitoring DNA damage accumulation and activation of downstream signalling when the cells are treated with G4 ligands. Instead, we use recovery‐from‐treatment assays, to monitor the repair of DSBs induced by pyridostatin. These assays show for the first time that, in the absence of BRCA2, pyridostatin‐induced DSBs are repaired by DNA‐PKcs‐dependent C‐NHEJ reactions. Whilst very effective, this type of repair is less accurate, introducing unstable DNA rearrangements (e.g. radial chromosomes) that potentially underlie pyridostatin toxicity against BRCA2‐deficient cells. On the other hand, C‐NHEJ repair may enable BRCA2‐deficient and tumours to develop resistance to pyridostatin, in the long term. Consistent with this, we find that targeting C‐NHEJ with DNA‐PKcs inhibitors enhanced the efficacy of pyridostatin treatment against BRCA1/2‐deficient tumours, thus the two compounds act synergistically in this context. These results are distinct from those reported by (Zimmer *et al*, 2016; Xu *et al*, 2017), which merely showed that abrogating HR or NHEJ sensitises the cells to the G4 ligands pyridostatin and CX‐5461, respectively.

In addition to its well‐documented ability to bind and stabilise G4 structures (Rodriguez *et al*, 2012; Chambers *et al*, 2015), pyridostatin has recently been proposed to inhibit TOP2 activity by trapping this enzyme on the DNA (Olivieri *et al*, 2020). Such dual mode of action is not uncommon among G4 ligands, having been also suggested for CX‐5461, which acts as an inhibitor of Pol I‐mediated rRNA synthesis (Drygin *et al*, 2011), in addition to binding G4s (Xu *et al*, 2017). More recently, CX‐5461 has been also annotated as a TOP2 inhibitor (Bruno *et al*, 2020). Conceivably, the DNA damage triggered by treatment with pyridostatin originates from replication fork stalling at genomic sites with G4 forming potential, or at sites where TOP2 is immobilised on chromatin. Both types of replication barriers induce DNA damage, which is particularly toxic to BRCA1/2‐deficient cells and tumours. Here, we demonstrate that, in the absence of BRCA1 or BRCA2, the DNA lesions inflicted by pyridostatin, regardless of origin, require C‐NHEJ reactions for their repair.

One key result reported in our study is that pyridostatin restricts the growth of BRCA1/2‐deficient tumours that have become resistant to PARPi. The molecular basis of this inhibition in the tumour model used in our study (allografts established using mouse tumour‐derived *Brca1* ^−/−^ *Tp53bp1* ^−/−^ cells; Jaspers *et al*, 2013) is loss of C‐NHEJ repair. We demonstrated this by examining recruitment to the chromatin of XRCC4, a component of the XRCC4/LigIV complex that provides a central C‐NHEJ activity (Ochi *et al*, 2014). Although HR is partially restored in BRCA1‐deficient cells upon loss of 53BP1 (Bunting *et al*, 2010), our results indicate that this is insufficient to repair DSBs induced by pyridostatin and that the toxicity of this drug stems from defective C‐NHEJ. Further strengthening the anti‐tumour activity of pyridostatin against PARPi‐resistant tumours is its inhibitory effect against *BRCA1*‐deleted PDTXs, which carry inactivating mutations in the *REV7 (MAD2L2)* gene (Bruna *et al*, 2016). The REV7 (MAD2L2) protein encoded by this gene promotes NHEJ at telomeres and during CSV recombination (Boersma *et al*, 2015; Xu *et al*, 2015), pointing to the key role of this repair pathway in mediating repair of pyridostatin‐induced damage in the context of PARPi resistance. Our results have profound clinical implications, because PARPi, although extremely effective in eliminating BRCA‐deficient tumours in the clinic (Ashworth & Lord, 2018), remain prone to acquired resistance. Counteracting PARPi‐resistant cancers currently represents a major clinical challenge. In addition to pyridostatin, the G4 ligand CX‐5461 (Zimmer *et al*, 2016; Xu *et al*, 2017), the alkylating agent chlorambucil (Tacconi *et al*, 2019), ionising radiation (Dev *et al*, 2018; Barazas *et al*, 2019) and cisplatin (Bunting *et al*, 2012; Cruz *et al*, 2018; Dev *et al*, 2018) have been reported as DNA damaging treatments that can counteract PARPi resistance in BRCA1/2‐deficient cells and tumours. However, only one clinical trial has so far investigated the cisplatin sensitivity in PARPi‐resistant BRCA1‐deficient tumours (Ang *et al*, 2013).

We further report here that pyridostatin triggers cGAS/STING‐dependent innate immune responses in the cells lacking BRCA1 or BRCA2. This recapitulates the effect of PARPi in the same genetic backgrounds (Reisländer *et al*, 2019) and is based on the ability of the two compounds to inflict DNA damage that triggers accumulation of cytoplasmic DNA recognised by the DNA sensor cGAS (Erdal *et al*, 2017; Mackenzie *et al*, 2017). In the case of PARPi, the ability to activate immune responses directly correlates with their anti‐tumour activity, as highlighted in pre‐clinical models of BRCA1‐deficient ovarian (Huang *et al*, 2015; Ding *et al*, 2018) and triple negative breast tumours (Pantelidou *et al*, 2019). The anti‐tumour effect of PARPi is enhanced in immuno‐competent mice, where PARPi increased the levels of infiltrating lymphocytes within the tumour and showed synergy with immune blockade inhibitors (Ding *et al*, 2018; Pantelidou *et al*, 2019). Our results with pyridostatin provide a strong rationale for testing it in combination with immune blockade drugs (e.g. anti‐PD‐1 or anti‐CTLA4 antibodies) for the treatment of BRCA1/2‐deficient tumours.

The DNA‐PKcs inhibitor NU‐7441 potentiated the toxicity of pyridostatin against BRCA2‐deficient cells. Evaluation of the same combination *in vivo* also showed an enhancement of the anti‐tumoral effect of pyridostatin by NU‐7441. However, this was followed by tumour re‐growth in the long term (after 30 days from initiation of treatment). We thus sought to combine pyridostatin with paclitaxel, a chemotherapeutic drug recently reported to stimulate apoptotic cGAS‐dependent responses via prolonged mitotic arrest (Zierhut *et al*, 2019), and also to increase immune responses via enhanced micronuclei formation resulting from chromosome mis‐segregation during mitosis (Weaver, 2014; Mitchison *et al*, 2017). Although tumour inhibition was initially observed, the combination was ineffective in suppressing tumour growth in the long term. Given that the pyridostatin/NU‐7441 and pyridostatin/paclitaxel combinations were well‐tolerated we assessed the pyridostatin/NU‐7441/paclitaxel combination. We found that treatment with the triple combination led to a persistent anti‐tumour response up to 63 days from initiation of treatment, reaching a maximum of 82% tumour volume inhibition (Fig 5C; Table 3). The superiority of the triple combination was emphasised when we applied the *m*odified *r*esponse *e*valuation *c*riteria in *s*olid *t*umours (mRECIST; Gao *et al*, 2015). According to these criteria, 5/5 mice had stable disease in response to pyridostatin/NU‐7441 and pyridostatin/paclitaxel combinations, whilst upon exposure to pyridostatin/NU‐7441/paclitaxel combination 4/5 mice showed a partial response and 1/5 showed a complete response to treatment (Table 3). Consistent with the effect of pyridostatin/NU‐7441/paclitaxel combination on tumour growth, we observed a significant increase of mice survival (149%) relative to the untreated cohort, whilst 47 and 40% increase in survival was observed in pyridostatin/paclitaxel‐ and pyridostatin/NU‐744‐treated groups, respectively.

When C‐NHEJ reactions are inhibited, DNA lesions inflicted by pyridostatin in BRCA1/2‐deficient cells and tumours persist even after compound removal. Therefore, DNA‐PKcs inhibition may represent an effective maintenance therapy following an initial tumour response to pyridostatin or its combinations with paclitaxel. While NU‐7441 was administrated intraperitoneally, other DNA‐PKcs inhibitors (e.g. CC‐115 or M3814, see below) currently used in clinical trials are administered orally, making them ideal candidates for a maintenance strategy. Because of the synergistic, long‐lasting benefits of the pyridostatin/NU‐7441/paclitaxel combination, multiple cycles of the triple combination or of the pyridostatin/paclitaxel combination, could be followed by daily DNA‐PKcs inhibitor administration as an alternative maintenance strategy, which can be further investigated.

Combinations of pyridostatin with WEE1 and USP1 inhibitors have recently been reported to act synergistically in inhibiting growth of cancer cells *in vitro* (Zyner *et al*, 2019). These, however, have not yet been tested in preclinical models. Our findings highlight the chemotherapeutic potential of pyridostatin/NU‐7441/paclitaxel combination to treat BRCA1/2‐deficient tumours. Among the three drugs, only paclitaxel is currently used in the clinic. NU‐7441, although a highly specific DNA‐PKcs inhibitor (Zhao *et al*, 2006; Davidson *et al*, 2013), is only suitable for laboratory use. Multiple DNA‐PKcs inhibitors have been developed and are currently tested in clinical trials (NCT02516813, NCT02316197, NCT01353625 and NCT02833883). Among these, the dual DNA‐PK/TOR kinase inhibitor CC‐115 (Mortensen *et al*, 2015), which targets ATM‐deficient cancer cells *in vitro* (Tsuji *et al*, 2017) showed promising response in phase I clinical trials. Whether its inhibitory effects extend to BRCA1/2‐deficient cells and tumours remains to be evaluated.

In summary, our study supports the suitability of pyridostatin for further clinical development, as a therapeutic strategy that can benefit cancer patients with *BRCA1/2*‐mutated and PARPi‐resistant disease.

## Materials and Methods

### Cell lines and growth conditions

*BRCA2* ^+/+^ and *BRCA2* ^−/−^ human colorectal adenocarcinoma DLD1 cells (Horizon Discovery; Zimmer *et al*, 2016), human breast carcinoma MDA‐MB‐436 cells were cultivated in monolayers in IMDM medium (Gibco) supplemented with 10% of foetal bovine serum (Life Technologies). *BRCA2* ^+/+^ and *BRCA2* ^−/−^ human colorectal carcinoma HCT116 cells (Ximbio, Cancer Research Technology) were grown in McCoy’s 5a media (Life Technologies) with 10% of foetal bovine serum and 1% of penicillin/streptomycin. *BRCA1* ^−/−^ and *BRCA2* ^−/−^ human retinal pigment epithelial RPE‐1 cells (transduced with hTERT and *TP53*‐deleted; (Zimmermann *et al*, 2018)) were cultivated in DMEM supplemented with 10% of foetal bovine serum in the presence of 2 μg/ml blasticidin (Life Technologies). *BRCA1/2*‐wild type or *BRCA1* ^−/−^ *TP53BP1* ^−/−^ RPE‐1 cells (a gift from Dr Stephen Jackson, Gurdon Institute, Cambridge; Dev *et al*, 2018) were cultivated in F12/DMEM supplemented with 10% of foetal bovine serum.

Human non‐small‐cell lung carcinoma H1299 carrying a doxycycline‐inducible shRNA against BRCA2 (Lai *et al*, 2017; Reisländer *et al*, 2019) were cultivated in monolayer in DMEM (Sigma Aldrich) supplemented with 10% of tetracycline‐free FBS (Takara Bio). Expression of shRNA against BRCA2 was induced by adding 2 µg/ml of doxycycline (Sigma‐Aldrich) in the growth medium. *PRKDC*‐deleted and *PRKDC* wild type near‐haploid HAP1 cells were cultivated in monolayers in DMEM medium (Sigma Aldrich) supplemented with 10% of foetal bovine serum (Life Technologies).

Mouse mammary tumour cell lines KP3.33 (*Brca1* ^+/+^ control), KB1PM5 (*Brca1* ^−/−^, PARP inhibitor‐sensitive) and KB1PM5 (*Brca1* ^−/−^ *Tp53bp1* ^−/−^, PARP inhibitor‐resistant; Jaspers *et al*, 2013) were cultured at 37°C, 5% of CO_2_ and 3% of oxygen in complete medium DMEM/F‐12 (Life Technologies) supplemented with 10% of foetal bovine serum (Life Technologies), 1% of penicillin/streptomycin (Sigma Aldrich), 5 μg/ml of insulin (Sigma Aldrich), 5 ng/ml epidermal growth factor (Life Technologies) and 5 ng/ml of cholera toxin (Gentaur). Cell lines were genotyped and routinely tested for mycoplasma contamination.

### CRISPR/Cas9‐mediated *BRCA2* knockout

RPE‐1 cells (transduced with hTERT, *TP53*‐deleted and expressing Cas9) were seeded in 96‐well plates at a density of 1 × 10^4^ cells per well. The following day, the cells were transfected with 50 nM of tracrRNA (U‐002005) plus crRNA targeting *BRCA2* (CM‐003462‐01, Dharmacon) using Dharmafect 1 (Dharmacon) according to manufacturer’s instructions. The cells were grown for 14–18 h and the medium was replaced with fresh growth medium. The cells were grown for 3 days and single‐cell clones were then established in 96‐well plates.

### siRNA transfection

DLD1 cells (8 × 10^5^) were reverse‐transfected with 40 nM of siRNA in 6‐cm dishes using Dharmafect 1 (Dharmacon) according to manufacturer’s instructions. In brief, 5 μl of Dharmafect reagent were added to 0.5 ml of Opti‐MEM reduced serum media (Thermo Fisher Scientific) and incubated at room temperature for 5 min. This solution was then mixed with 0.5 ml of Opti‐MEM reduced serum media containing siRNA, incubated at room temperature for 20 min and then added to the cell suspension. The cells were grown for 24 h and the medium was replaced with fresh growth medium. AllStars negative control siRNA was obtained from Qiagen. POLQ siRNA was obtained from Dharmarcon (UUU CAU AUA AAC AUU CUG G).

### *In vivo* xenograft experiments

CB17‐SCID mice (CB17/Icr‐Prkdcscid/IcrIcoCrl, male or female), FVB female mice were purchased from Charles River Laboratories (Calco, Italy). The mice were maintained in high‐efficiency, particulate air HEPA‐filtered racks and were fed autoclaved laboratory rodent diet. All animal procedures were in compliance with the national and international directives (D.L. March 4, 2014, no. 26; directive 2010/63/EU of the European Parliament and of the council; Guide for the Care and Use of Laboratory Animals, United States National Research Council, 2011).

To generate xenografts derived from DLD1 and HCT116 BRCA2‐proficient or ‐deficient cells, CB17‐SCID male mice 6 weeks old were injected intramuscularly, into the hind leg muscles, with 5 × 10^6^ cells per mouse. When a tumour volume of approximately 250 mm^3^ was evident, mice were randomised to start the treatments.

To generate the PARPi‐resistant mouse tumour model, FVB female mice 6 weeks old were injected intramuscularly into the hind leg muscles with 4 × 10^6^ KP3.33 (*Brca1* ^+/+^) cells or KB1PM5 (*Brca1* ^−/−^ *Tp53bp1* ^−/−^) mouse mammary tumour cells. Each experimental group included five mice. When a tumour volume of approximately 250 mm^3^ was evident, mice were randomised and the treatment started.

To generate xenografts derived from MDA‐MB‐436 cells, CB17‐SCID female mice 6 weeks old were injected intramuscularly with 4 × 10^6^ cells per mouse. When a tumour volume of approximately 220 mm^3^ was evident (6 days after cell injection), treatment was initiated. Each experimental group included five mice.

Talazoparib (BMN 673, Selleckchem) was dissolved in 10% of dimethylacetamide, 6% of solutol HS, 84% of PBS and administered orally at doses of 0.33 mg/kg/day for five consecutive days, followed by 2‐day break and five more days of treatment (Wang *et al*, 2016). Pyridostatin (Sigma‐Aldrich) was dissolved in saline solution and administered intravenously at doses of 7.5 mg/kg/day for five consecutive days, followed by 2‐day break and five more days of treatment. NU‐7441 (Selleckchem) was dissolved in 5% of DMSO, 40% PEG300, 5% of Tween‐80 and administered intraperitoneally at doses of 10 mg/kg/day for five consecutive days, followed by 2‐day break and five more days of treatment (Zhao *et al*, 2006). Paclitaxel (Sandoz S.p.A, Novartis) was dissolved in saline solution and administered intravenously at doses of 20 mg/kg/day at day 1 and day 8 of treatment (Bizzaro *et al*, 2018). When combined with other compounds, paclitaxel was administered intravenously at day 5 and 12 of treatment, pyridostatin and NU‐7441 were administered intravenously and intraperitoneally, respectively, for four consecutive days, followed by a 3‐day break and four more days of treatment. NU‐7441 was administered 2 h before pyridostatin. At indicated time points, tumour volumes were measured in two dimensions using a caliper and tumour weight was estimated from tumour volume (1 mg = 1 mm^3^). The student’s *t*‐test (unpaired, two‐tailed) was used for single pair‐wise comparisons. Differences were considered statistically significant when *P* < 0.05. Survival curves of mice were processed using the Kaplan–Meier method, and statistical significance was assessed by log‐rank test. Data were plotted using GraphPad Prism Software 8.3.

### Generation of PDTX models

Fresh tumour samples from patients with gBRCA breast cancer were prospectively collected for implantation into mice under an institutional IRB‐approved protocol and the associated informed consent, or by the National Research Ethics Service, Cambridgeshire 2 REC (REC reference number: 08/H0308/178) (Bruna *et al*, 2016).

The VHI0179 patient‐derived tumour xenografts (PDTXs) were generated from a patient breast tumour with a BRCA1 germline truncation and resistant to Olaparib due to REV7 mutation. Written informed consent was obtained from all patients and the experiments conformed to the principles set out in the WMA Declaration of Helsinki and the Department of Health and Human Services Belmont Report. Frozen tumour fragments (15–20 mm^3^) were coated in Matrigel (Corning) and implanted using a small incision in a subcutaneous pocket made in one side of the lower back into one CB17‐SCID female mice 6 weeks old. When the tumour reached approximately 400 mm^3^, tumour was explanted from the sacrificed mouse, cut into fragments of about 15–20 mm^3^ and implanted again subcutaneously in fourteen CB17‐ SCID female mice. When the tumour reached approximately 200 mm^3^, mice were randomised in vehicle and treated group to start the treatments. Each experimental group included seven mice.

### *Ex vivo* drug experiments

The *ex vivo* drug treatment protocol was performed as previously described (Bruna *et al*, 2016). Briefly, frozen patient‐derived tumour xenografts (PDTXs) were thawed and dissociated into single cell suspensions by combining mechanical and enzymatic dissociation using the soft tumour dissociation protocol on a GentleMACS Dissociator and the human tumour dissociation kit (Miltenyi Biotec, Cat ID 130‐093‐235) according to the manufacturer instructions. Single cells were plated at ~40,000 cells/ml in 50 µl per well in 384 well plates and dosed 72 h after plating. The selected drugs were added to the wells after 24 h of seeding using Echo Liquid Handler 550 (Labcyte). Cell viability was assessed using CellTiterGlo 3D Cell Viability (Promega) 6–10 days after dosing following manufacturer specifications and normalised against blank wells and control wells treated by Dimethyl Sulfoxide (DMSO, Sigma). Plates were read on the Pherastar plate reader using the Luminescence module.

### Immunoblotting

The cells were harvested with trypsin and washed in PBS. The cells were then lysed using loading buffer supplemented with 100 mM of DTT, protease‐ and phosphatase inhibitor cocktails. Samples were briefly sonicated using a probe sonicator, heated to 70°C for 10 min and centrifuged at > 10,000 *g* for 7 min. Equal amounts of protein was loaded on NuPAGE‐Novex 10, 12 or 4–12% of Bis‐Tris and NuPAGE‐Novex 3–8% of Tris‐Acetate gels (Life Technologies), which were run according to manufacturer’s instructions. Proteins were transferred onto a nitrocellulose membrane (30 V, 100 min). The membrane was blocked in 5% of milk dissolved in PBST, incubated with primary antibodies and subsequently with HRP‐conjugated secondary antibodies. Detection was achieved by enhanced chemiluminescence detected on X‐ray films.

### Antibodies

The following antibodies were used for immunoblotting: rabbit polyclonal antibody raised against 53BP1 (1:5,000, NB100‐304, Novus Biologicals), DNA‐PKcs (1:1,000, A300‐519A, Bethyl Laboratories), phosphorylated KAP1 (Ser824) (1:1,000, A300‐767A, Bethyl Laboratories), KAP1 (1:5,000, A300‐274A, Bethyl Laboratories), phosphorylated RPA (Ser4/Ser8) (1:4,000, A300‐245A, Bethyl Laboratories), phosphorylated IRF3 (Ser386) (1:1,000, ab76493, Abcam), IRF3 (1:1,000, ab76409, Abcam), phosphorylated STAT1 (Tyr701) (1:1,000, 9167, Cell Signalling), STAT1 (1:1,000, 9175, Cell Signalling), SMC1 (1:5,000, BL308, Bethyl Laboratories); mouse monoclonal antibodies raised against XRCC4 (1:1,000, 611506, BD biosciences), BRCA2 (1:1,000, OP95, Calbiochem), RPA (1:1,000, ab2175, Abcam), GAPDH (1:30,000, 6C5, Novus Biologicals), α‐Tubulin (1:30,000, TAT‐1, Cancer Research UK Monoclonal Antibody Service) and POLQ (1:10,000, (Fernandez‐Vidal *et al*, 2014)).

### Resazurin‐based cell viability assays

The cells were seeded in 96‐well plates using cell numbers that enabled untreated cells to reach 80–90% confluency at the end of the assay. The following day, the cells were treated as indicated. Six days later, the cells were incubated in a medium containing 10 μg/ml of resazurin for 2 h. Cell viability was determined by fluorescence (590 nm) using a plate reader (POLARstar, Omega). Cell viability was expressed relative to mock‐treated cells of the same genotype.

### Clonogenic survival assays

*BRCA2* ^+/+^ and *BRCA2* ^−/−^ DLD1 cells were plated in technical triplicate at densities between 200 and 4,000 cells per well in 6‐well plates. Drug treatment was initiated after the cells had adhered. Colonies were stained with 0.5% of crystal violet in 50% of methanol and 20% of ethanol in dH2O after 10–14 days. The cell survival was expressed relative to untreated cells of the same cell line.

Human MDA‐MB‐436 cells were seeded at a density of 1 × 10^5^ cells per dish in 60‐mm dishes. On the next day, cells were treated with 5 µM of NU‐7441, 0.3 µM of pyridostatin or 0.1–3 nM of paclitaxel for 24 h. For NU‐7441 plus pyridostatin or NU‐7441 plus paclitaxel combinations, drugs were added simultaneously for 24 h. For pyridostatin plus paclitaxel combination, the cells were treated with pyridostatin for 24 h and subsequently treated with paclitaxel for 24 h. For NU‐7441 plus pyridostatin plus paclitaxel combination, the cells were treated with NU‐7441 plus pyridostatin for 24 h and the medium was subsequently treated with paclitaxel for 24 h. To evaluate cell colony‐ forming ability, at the end of treatments, the cells were lifted with trypsin and seeded in technical triplicate at density of 1,000 cells per dish. Colonies were stained with 2% of methylene blue in 60% of ethanol after 13 days and counted (> 50 cells equalled one colony).

### Preparation of metaphase chromosome spreads

The cells were arrested in mitosis by overnight incubation with 0.1 μg/ml KaryoMAX colcemid (Life Technologies). They were collected by mitotic shake‐off and swollen in hypotonic buffer (0.03 M of sodium citrate) at 37°C for 25 min. Next, the cells were fixed in freshly‐prepared 3:1 mix of methanol:glacial acetic acid and nuclear preparations were dropped onto slides pre‐soaked in 45% of acetic acid prior to being allowed to dry overnight. The following day, mitotic chromosomes were stained using Giemsa (VWR) and viewed with a Leica DMI6000B inverted microscope equipped with a HCX PL APO 100×/1.4–0.7 oil objective.

### Alkaline comet assay

Alkaline comet assays were performed as previously described (Parsons *et al*, 2009). In brief, 2 × 10^5^ cells were embedded in 1% low‐melting agarose in PBS on a microscope slide. Subsequently, the cells were lysed in buffer containing 2.5 M of NaCl, 100 mM of EDTA, 10 mM of Tris‐HCl pH 10.5, 1% of DMSO and 1% of Triton X‐100 for 1 h at 4°C. To denature the DNA, the slides were incubated in cold electrophoresis buffer (300 mM of NaOH, 1 mM of EDTA, 1% of DMSO, pH > 13) for 30 min in the dark. Following electrophoresis at 25 V and 300 mA for 25 min, the DNA was neutralised with 0.5 M of Tris‐HCl pH 8.0. After staining with SYBR Gold, specimens were viewed using a Nikon eclipse Ni‐E microscope. Tail measurements were performed using the Komet 5.5 image analysis software (Andor Technology).

### Flow cytometry

Asynchronous cells were pulse‐labelled 25 μM of EdU for 30 min. The cells were harvested with trypsin, washed with PBS and fixed using 90% of methanol. Incorporated EdU was detected using the Click‐iT EdU Alexa Fluor 647 Flow Cytometry Assay Kit (Invitrogen) according to manufacturer’s instructions. The cells were re‐suspended in PBS containing 20 μg/ml of propidium iodide and 400 μg/ml of RNaseA. Samples were processed using flow cytometry. 10,000 events were analysed per condition using FlowJo software.

### Immunofluorescence

The cells were grown on coverslips, washed in PBS and lysed in hypotonic solution (85.5 mM of NaCl and 5 mM of MgCl_2_) for 5 min at room temperature. Next, the cells were fixed with 4% of paraformaldehyde for 10 min at RT, then permeabilised in 4% of paraformaldehyde supplemented with 0.03% of SDS. Coverslips were rinsed with PBS with 0.4% of Photoflo and briefly incubated in antibody dilution buffer (1% of goat serum, 0.3% of BSA, 0.005% of Triton X‐100 in PBS). The cells were incubated with anti‐γH2AX (1:2,000, 05‐636, Merck Millipore), anti‐53BP1 (1:5,000, NB100‐304, Novus Biologicals), anti‐RAD51 (1:5,000, 70‐001, Bio Academia) or with anti‐cGAS (1:200, 15102, Cell Signalling) antibody overnight at room temperature, rinsed with PBS plus 0.4% Photoflo and incubated with Alexa Fluor‐conjugated secondary antibodies (1:400, Thermo Fisher Scientific) secondary antibody for 1 h at room temperature. Coverslip were rinsed three times, dried and mounted on microscope slides using Prolong Antifade Gold supplemented with DAPI (Thermo Fisher Scientific). Specimens stained with anti‐γH2AX‐ and anti‐53BP1 antibodies were imaged with an inverted microscope (Leica DMI6000B) and fluorescence imaging workstation equipped with a HCX Plan‐ Apochromat 100×/1.4–0.7 oil objective. Images were acquired using a DFC350 FX R2 digital camera (Leica) and LAS‐AF software (Leica). Specimen stained with anti‐cGAS antibody was imaged with an upright Nikon Ni‐E microscope with a CoolLED PE‐4000 for fluorescence excitation and an Andor Zyla 4.2 Plus camera. Images were acquired using a 60× magnification 1.4 NA Plan‐ApoChromat objective using the Nikon NIS Elements software. Quantifications were performed using ImageJ (National Institutes of Health, USA).

### DNA fibre assay

DNA was first pulse‐labelled with 25 μM of CldU (Sigma‐Aldrich # C6891) for 30 min and subsequently pulse‐labelled with 250 μM of IdU (Sigma‐Aldrich #I7125) for 30 min, followed by incubation for 5 h with 2 µM of pyridostatin or 2 mM of HU, in the presence or absence of 25 µM of mirin. The cells were washed with warm PBS three times in between treatments with thymidine analogues CldU and IdU, as well as hydroxyurea and pyridostatin. The cells were harvested with trypsin and 0.5 × 10^6^ cells were resuspended in cold PBS. Next, 7 μl of lysis buffer (200 mM of Tris‐HCl pH 7.4, 50 mM of EDTA, 0.5% of SDS) were mixed with 2‐μl cell suspension on a microscopy slide and incubated horizontally for 7 min at room temperature. DNA was spread by tilting the slide manually at an angle of approximately 30°. Slides were air‐dried and incubated in methanol/acetic acid (3:1) for 10 min. Slides were then rehydrated with PBS (2 × 3 min) and DNA was denatured in 2.5 M of HCl (VWR #20252.244) for 1 h at room temperature. Slides were washed several times in PBS until a pH of 7–7.5 was reached, followed by incubation in blocking solution (2% BSA, 0.1% Tween 20 (Sigma‐Aldrich #P7949) in PBS) for 40 min at room temperature and in rat anti‐CldU (1:500, ab6326, Abcam) and mouse anti‐IdU (1:100, 347580, Becton Dickinson) primary antibodies for 2.5 h at room temperature. After five washes in PBS‐Tween (0.2% of Tween 20 in PBS) for 3 min and one short wash in blocking solution, the slides were incubated with anti‐rat Cy3 (1:300, 712‐165‐153, Jackson Immuno Research) and anti‐mouse Alexa Fluor 488‐conjugated (1:300, R37120, Invitrogen) secondary antibodies for 1 h at room temperature. Slides were then washed as before, air‐dried and mounted in Prolong Antifade Gold (Thermo Fisher Scientific #P36930). Images were acquired and analysed as described for immunofluorescence.

### Immunohistochemistry

Tissues collected from experimental mice were fixed in 10% of formalin pH 7.4 (VWR Chemicals) for 24 h before dehydrating in 70% of ethanol for 24 h and embedding in paraffin (ThermoScientific, Histostar and Histoplast parafin). Formalin‐fixed and paraffin‐embedded tissue samples were sectioned onto slides (ThermoScientific Superfrost Ultra‐Plus) at 3–5 µm thickness (for DLD1 tumours) or at 2 µm thickness (for HCT116 tumours). Immunohistochemistry was performed by de‐paraffinising (two incubations in xylene, for 3 min each), dehydrating (two incubations in 100% of EtOH, for 3 min each) and rehydrating the slides (successive incubations in 95, 90, 70 and 50% of EtOH, for 3 min each). Controlled antigen retrieval was induced with citrate buffer (pH 6.0) for 2 min at 110°C (BioCare Medical, for DLD1 tumours) or at high pH by PT Link (Dako Omnis, for HCT116 tumours). Endogenous peroxidase was blocked for 10 min using peroxidase‐blocking solution (Dako Omnis) and non‐specific antibody binding sites were blocked for 20 min using protein blocking buffer (Dako Omnis). For DLD1 tumours, slides were incubated with anti‐phosphorylated H2AX (Ser139) (1:500, ab11174, Abcam) overnight at 4°C. For HCT116 tumours, slides were incubated with anti‐phosphorylated H2AX (Ser139) (1:300, #9718, 20E3, Cell Signalling,). Next, slides were covered with secondary antibody (Dako Omnis) for 30 min and the signal was developed using diaminobenzidine as chromogen substrate (EnVision™ FLEX Dako Omnis). Tumour sections were counterstained with haematoxylin. After chromogen development, slides were washed, dehydrated with alcohol and xylene and mounted with coverslips using a permanent mounting medium. Immunostaining results were recorded as percentage of the cells positive for γH2AX staining.

### Quantitative RT‐PCR

The cells were grown in a 6‐well plate and harvested with trypsin and washed in PBS. For RNA reverse transcription, the Ambion Kit Power SYBR™ Green Cells‐to‐CT™ Kit (#4402954) was used according to manufacturer’s instructions. Quantitative PCR was performed in technical triplicate using SYBR Green technology on the StepOnePlus Real‐Time PCR System (Thermo Fisher Scientific). Gene expression was normalised to *GAPDH* and calculated relative to control cells as 2^−ΔΔCT^. The following primer pairs were used: *IFIT1*, forward TAC CTG GAC AAG GTG GAG AA and reverse GTG AGG ACA TGT TGG CTA GA; *IFIT2*, forward TGT GCA ACC TAC TGG CCT AT and reverse TTG CCA GTC CAG AGG TGA AT; *ISG15*, forward GCG AAC TCA TCT TTG CCA GTA and reverse CCA GCA TCT TCA CCG TCA G; *GAPDH*, forward GAC AGT CAG CCG CAT CTT CT and reverse ACC AAA TCC GTT GAC TCC GA.

## Author contributions

**Florian, J Groelly:** Formal analysis; Validation; Investigation; Methodology; Writing—original draft; Writing—review & editing. **Manuela Porru:** Formal analysis; Validation; Investigation; Methodology; Writing—original draft; Writing—review & editing. **Jutta Zimmer:** Formal analysis; Investigation; Methodology. **Hugo Benainous:** Formal analysis; Investigation; Methodology. **Yanti De Visser:** Formal analysis; Investigation. **Anastasiya Kosova:** Formal analysis; Investigation. **Serena Di Vito:** Formal analysis; Investigation; Methodology. **Violeta Serra:** Resources; Methodology. **Anderson Ryan:** Resources; Methodology. **Carlo Leonetti:** Investigation; Methodology. **Alejandra Bruna:** Formal analysis; Investigation; Methodology. **Annamaria Biroccio:** Conceptualization; Supervision; Funding acquisition; Investigation; Methodology. **Madalena Tarsounas:** Conceptualization; Resources; Supervision; Funding acquisition; Investigation; Visualization; Methodology; Writing—original draft; Writing—review & editing.

In addition to theCRediTauthor contributions listed above, the contributions in detail are:

Conception and design: ABi and MT. Acquisition of data (including provided animals, provided facilities etc.): MP, FJG, JZ, HB, YDV, AAK, SDV, VS, AR, CL, ABr. Writing the manuscript: FJG, MT. Study supervision: ABi and MT.

## Supporting information



Appendix

Source Data for Appendix

Source Data for Figure 1

Source Data for Figure 2

Source Data for Figure 3

## Data Availability

This study includes no data deposited in external repositories.
