# Supplementary material for: Anti‐tumoural activity of the G‐quadruplex ligand pyridostatin against BRCA1/2‐deficient tumours
Source: EMBO Mol Med. 2022 Feb 2;14(3):e14501. doi: 10.15252/emmm.202114501 (PMC8899905; doi:10.15252/emmm.202114501)
Supplement: Supplementary file 1 — Appendix [file EMMM-14-e14501-s004.pdf]

Appendix – Groelly, Porru et al.

TABLE OF CONTENT

- **Appendix Figure S1.** Pyridostatin inhibits growth of BRCA2-deficient HCT116 cell derived xenograft tumours and inflicts DNA damage in BRCA2-deficient tumours.

- **Appendix Figure S2.** Pyridostatin triggers stalled fork degradation and repairable DNA breakage in BRCA2-deficient cells.

- **Appendix Figure S3.** The ATM-activating DNA damage induced by pyridostatin in human BRCA2-deficient cells is repaired after drug removal.

- **Appendix Figure S4**. POLQ is not required to prevent or repair pyridostatin-induced DNA damage in BRCA2-deficient cells.

- **Appendix Figure S5**. Specificity of the DNA-PKcs inhibitor NU-7441.

- **Appendix Figure S6**. CRISPR/Cas9-mediated *BRCA2* knockout in RPE-1 cells.

- **Appendix Figure S7.** NHEJ abrogation in PARPi-resistant cells and the anti-proliferative effect of pyridostatin, NU-7441 and paclitaxel combinations *in vitro*.

- **Appendix Figure S8**. Effect of pyridostatin, NU-7441 and paclitaxel combinations in BRCA2-proficient HCT116 xenograft models.

- **Appendix Table S1.** *In vivo* anti-tumour efficacy of pyridostatin and talazoparib on $BRCA2^{+/+}$ and $BRCA2^{-/-}$ DLD1 xenografts.

- **Appendix Table S2.** *In vivo* anti-tumour efficacy of pyridostatin on $BRCA2^{+/+}$ and $BRCA2^{-/-}$ HCT116 xenografts.

- **Appendix Table S3**. Statistical analysis of the survival advantage conferred by pyridostatin, NU-7441, paclitaxel and their combination on MDA-MB-436 xenografts.

- **Appendix Table S4**. *In vivo* anti-tumour efficacy of pyridostatin, NU-7441, paclitaxel and their combination on HCT116 *BRCA2*$^{+/+}$ xenografts.

- **Appendix Table S5**. Statistical analysis of the survival advantage conferred by pyridostatin, NU-7441, paclitaxel and their combination on HCT116 *BRCA2*$^{+/+}$ xenografts.

- **Appendix Table S6**. Statistical analysis of the survival advantage conferred by pyridostatin, NU-7441, paclitaxel and their combination on HCT116 *BRCA2*$^{-/-}$ xenografts.

- **Appendix Table S7.** *In vivo* anti-tumour efficacy of pyridostatin and CX-5461 on DLD1 *BRCA2*$^{-/-}$ and HCT116 *BRCA2*$^{-/-}$ xenografts.

- **Appendix Table S8.** Exact *P* values from figures.

Appendix Figure S1. Pyridostatin inhibits growth of BRCA2-deficient HCT116 cell derived xenograft tumours and inflicts DNA damage in BRCA2-deficient tumours.

A,B CB17-SCID male mice were injected intramuscularly with (A) BRCA2-proficient (*BRCA2*$^{+/+}$) or (B) BRCA2-deficient (*BRCA2*$^{-/-}$) HCT116 cells. Pyridostatin (PDS) was administered intravenously (*i.v.*) over the indicated periods of time. Vertical dotted line indicates end of treatment. Tumour volume was measured at the indicated timepoints and expressed relative to tumour volume at the beginning of treatment. Each experimental group included n = 5 mice. Error bars represent SEM. P values were calculated between treated and untreated tumours at day 17, using an unpaired two-tailed t-test. ****, $P \leq 0.0001$; *NS*, $P > 0.05$.

C Immunohistochemical detection of γH2AX staining in sections from BRCA2-proficient (*BRCA2*$^{+/+}$) or BRCA2-deficient (*BRCA2*$^{-/-}$) DLD1 xenograft tumours treated with pyridostatin (PDS) or talazoparib, as shown in Figure 1 A,B. Representative images are shown. Scale bar represents 50 µm.

D Quantification of γH2AX staining shown in (C).

E Immunohistochemical detection of γH2AX staining in sections from BRCA2-proficient (*BRCA2*$^{+/+}$) or BRCA2-deficient (*BRCA2*$^{-/-}$) HCT116 xenograft tumours treated with pyridostatin (PDS). Representative images are shown. Scale bar represents 30 µm.

F Quantification of γH2AX staining shown in (E). For each group, graph and error bars represent the mean and SEM of 32 sections from n = 4 mice. P values were calculated using an unpaired two-tailed t-test. ****, $P \leq 0.0001$; NS, $P > 0.05$.

Exact P values for (A, B, F) are provided in Appendix Table S8.

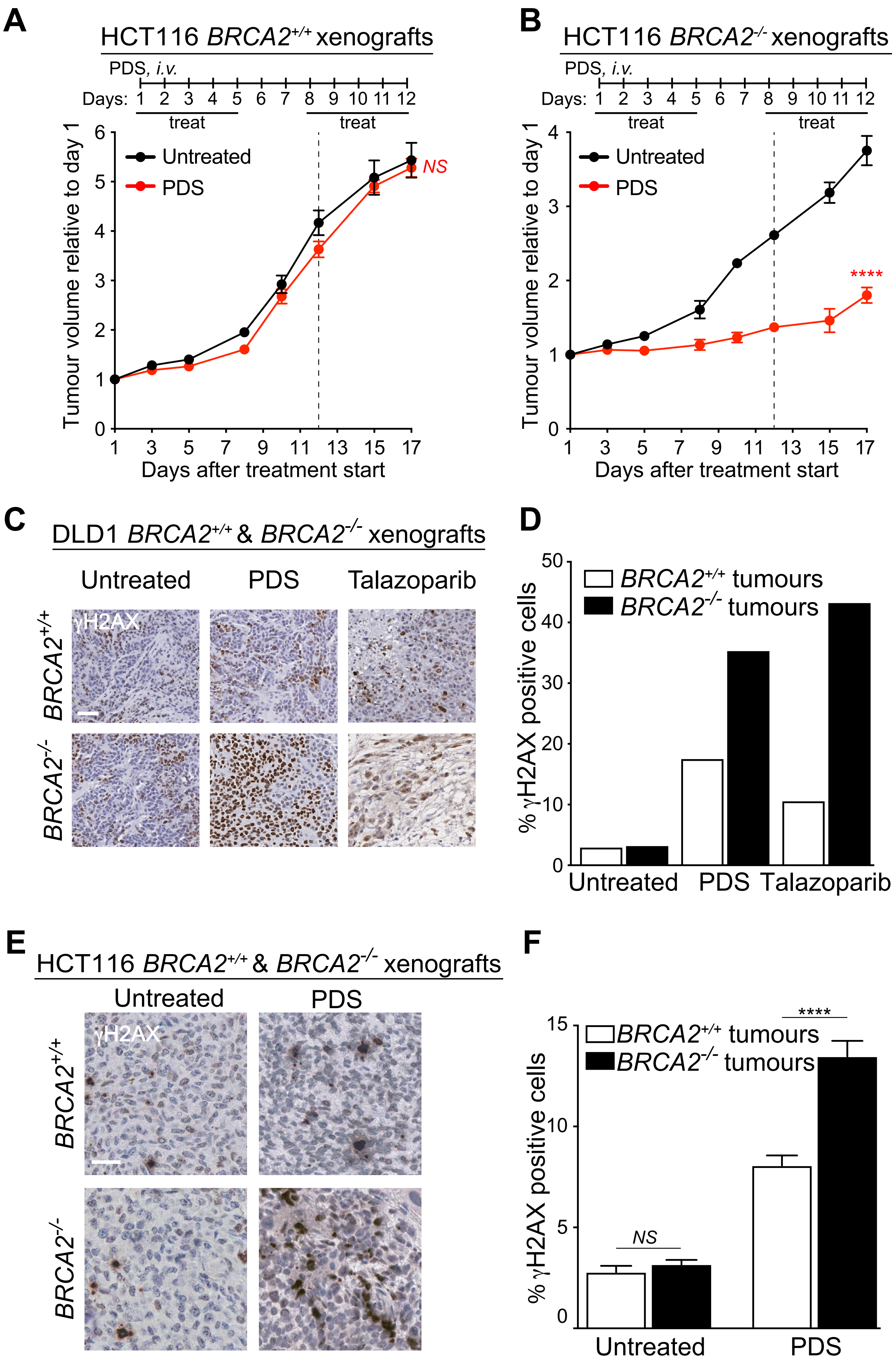
A
HCT116 BRCA2+/+ xenografts
PDS, i.v.
Days: 1 2 3 4 5 6 7 8 9 10 11 12
treat
treat
Tumour volume relative to day 1
Untreated
PDS
NS
Days after treatment start
B
HCT116 BRCA2-/- xenografts
PDS, i.v.
Days: 1 2 3 4 5 6 7 8 9 10 11 12
treat
treat
Tumour volume relative to day 1
Untreated
PDS
****
Days after treatment start
C
DLD1 BRCA2+/+ & BRCA2-/- xenografts
Untreated
PDS
Talazoparib
γH2AX
BRCA2+/+
BRCA2-/-
D
% γH2AX positive cells
BRCA2+/+ tumours
BRCA2-/- tumours
Untreated
PDS
Talazoparib
E
HCT116 BRCA2+/+ & BRCA2-/- xenografts
Untreated
PDS
γH2AX
BRCA2+/+
BRCA2-/-
F
% γH2AX positive cells
BRCA2+/+ tumours
BRCA2-/- tumours
NS
****
Untreated
PDS

Appendix Figure S2. Pyridostatin triggers stalled fork degradation and repairable DNA breakage in BRCA2-deficient cells.

A BRCA2-proficient (+BRCA2) or -deficient (-BRCA2) human DLD1 cells were pulse-labelled with CldU for 30 min, followed by IdU for 30 min. They were then incubated with 2 mM hydroxyurea (HU) or 2 µM pyridostatin (PDS), in the presence or absence of 25 µM mirin for 5 hours. Fork stability was determined using a DNA fibre assay. *P* values were calculated using a Mann-Whitney test. ****, $P \leq 0.0001$. *NS*, $P > 0.05$.

B Chromosome aberrations and chromosome breaks were visualised using Giemsa staining in BRCA2-proficient (+BRCA2) or -deficient (-BRCA2) human DLD1 cells treated with 2 µM pyridostatin (PDS) for 16 hours and released into fresh medium without pyridostatin. Representative images are shown. Scale bar represents 10 µm.

C Quantification of chromosome aberrations as shown in (B). Data were obtained from of $n = 3$ independent experiments. A minimum of 150 Giemsa-stained metaphases were analysed per condition. Red bars represent mean frequencies of chromosome aberrations. *P* values were calculated using a Mann-Whitney test. ***, $P \leq 0.001$; *NS*, $P > 0.05$.

Exact *P* values for (A, C) are provided in Appendix Table S8.

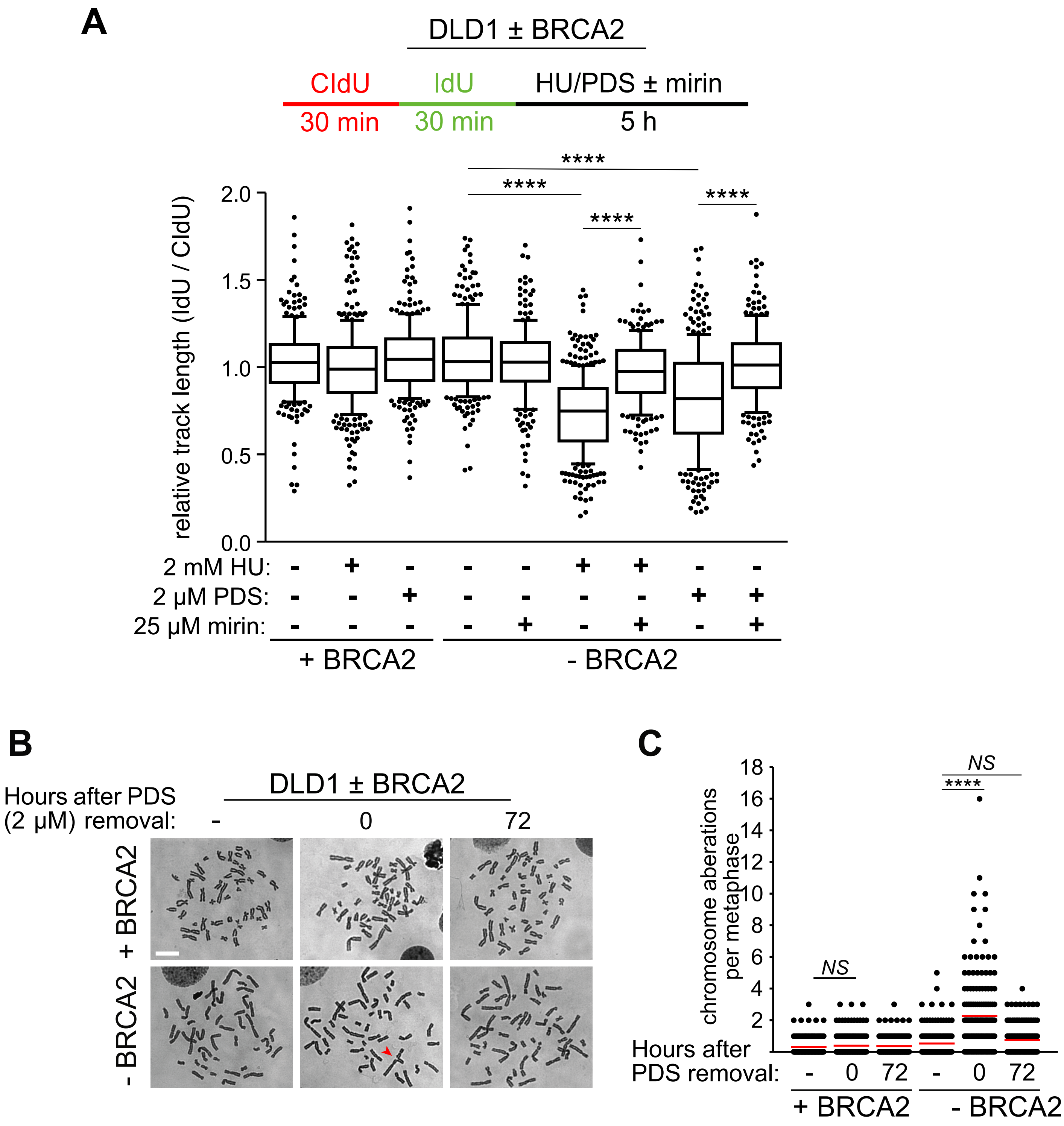
A
DLD1 ± BRCA2
CldU
IdU
HU/PDS ± mirin
30 min
30 min
5 h
****
****
****
****
relative track length (IdU / CldU)
2.0
1.5
1.0
0.5
0.0
2 mM HU: - + - - - + + - -
2 μM PDS: - - + - - - - + +
25 μM mirin: - - - - + - + - +
+ BRCA2
- BRCA2
B
DLD1 ± BRCA2
Hours after PDS
(2 μM) removal: - 0 72
+ BRCA2
- BRCA2
C
chromosome aberations
per metaphase
18
16
14
12
10
8
6
4
2
NS
****
NS
Hours after
PDS removal: - 0 72 - 0 72
+ BRCA2
- BRCA2

Appendix Figure S3. The ATM-activating DNA damage induced by pyridostatin in human BRCA2-deficient cells is repaired after drug removal.

A BRCA2-proficient (+BRCA2) or -deficient (-BRCA2) human DLD1 cells were treated with 2 μM pyridostatin (PDS) for 24 hours and released into fresh medium without pyridostatin. Whole-cell extracts were prepared at the indicated timepoints after release and immunoblotted as shown. KAP1 and RPA phosphorylation sites are shown in red. SMC1 and GAPDH were used as loading controls.

B BRCA2-proficient (+BRCA2) or -deficient (-BRCA2) human DLD1 cells treated with 2 μM pyridostatin (PDS) for 16 hours and released into fresh medium without pyridostatin before being prepared for immunofluorescence with antibodies against γH2AX and 53BP1. DNA was counterstained with DAPI. Scale bar represents 20 μm.

C Quantification of 53BP1 foci visualised using immunofluorescence staining in cells treated as in (B). A minimum of 200 cells were analysed per condition and experiment. Graph and error bars represent the mean and SEM of $n = 3$ independent experiments. P values were calculated using an unpaired two-tailed t-test. **, $P \leq 0.01$; ***, $P \leq 0.001$; *NS*, $P > 0.05$.

D BRCA2-proficient (+BRCA2) or -deficient (-BRCA2) human DLD1 cells were treated with 2 μM pyridostatin (PDS) for 16 hours and released into fresh medium without pyridostatin. They were then pulse-labelled with EdU and stained with propidium iodide. The fraction of cells in G2/M was determined using flow cytometry analysis and expressed relative to untreated BRCA2-proficient cells. Error bars represent the SEM of $n = 3$ independent experiments. P values were calculated using an unpaired two-tailed t-test. **, $P \leq 0.01$; *NS*, $P > 0.05$.

Exact P values for (C, D) are provided in Appendix Table S8.

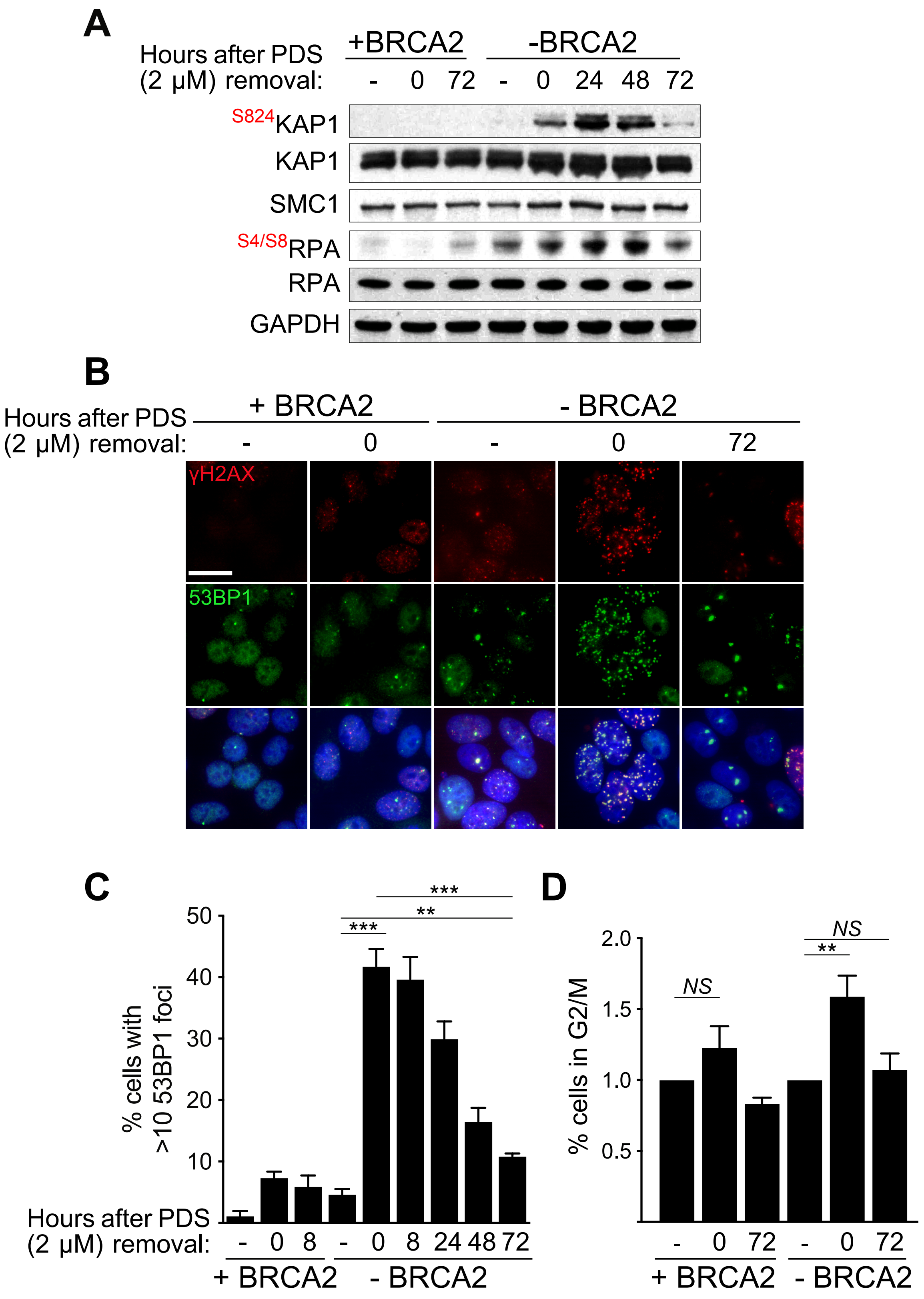
A
Hours after PDS
(2 μM) removal:
+BRCA2
-BRCA2
- 0 72 - 0 24 48 72
S824KAP1
KAP1
SMC1
S4/S8RPA
RPA
GAPDH
B
Hours after PDS
(2 μM) removal:
+ BRCA2
- BRCA2
- 0 - 0 72
γH2AX
53BP1
C
% cells with
>10 53BP1 foci
50
40
30
20
10
***
**
***
Hours after PDS
(2 μM) removal:
- 0 8 - 0 8 24 48 72
+ BRCA2
- BRCA2
D
% cells in G2/M
2.0
1.5
1.0
0.5
NS
NS
**
- 0 72 - 0 72
+ BRCA2
- BRCA2

Appendix Figure S4. POLQ is not required to prevent or repair pyridostatin-induced DNA damage in BRCA2-deficient cells.

A BRCA2-proficient (+BRCA2) and -deficient (–BRCA2) DLD1 cells transfected with control or POLQ siRNA were immunoblotted as indicated. SMC1 was used as a loading control.

B Clonogenic survival assays of cells treated as in (A) and exposed to indicated concentrations of pyridostatin. Error bars represent SEM of n = 3 independent experiments.

C BRCA2-proficient (+BRCA2) and -deficient (–BRCA2) DLD1 cells transfected with control or POLQ siRNA were treated with 2 μM pyridostatin for 16 h before prepared for immunofluorescence. Cells with > 20 γH2AX foci were quantified as shown. At least 200 cells were analysed per condition and experiment. Error bars represent SEM of n = 3 independent experiments. P values were calculated using an unpaired two-tailed t-test. *NS*, $P > 0.05$.

D Cells treated as in (C) with > 10 53BP1 foci were quantified as shown. At least 200 cells were analysed per condition and experiment. Error bars represent the SEM of n = 3 independent experiments. P values were calculated using an unpaired two-tailed t-test. *NS*, $P > 0.05$.

E BRCA2-proficient (+BRCA2) and -deficient (–BRCA2) DLD1 cells transfected with control or POLQ siRNA were treated with 2 μM pyridostatin for 16 h and released into fresh media for 72 hours before processing for immunofluorescence. Cells with > 20 γH2AX foci were quantified as shown. At least 200 cells from n = 1 experiment were analysed per condition.

F Cells treated as in (E) with > 10 53BP1 foci were quantified as shown. At least 200 cells from n = 1 experiment were analysed per condition.

Exact P values for (C, D) are provided in Appendix Table S8.

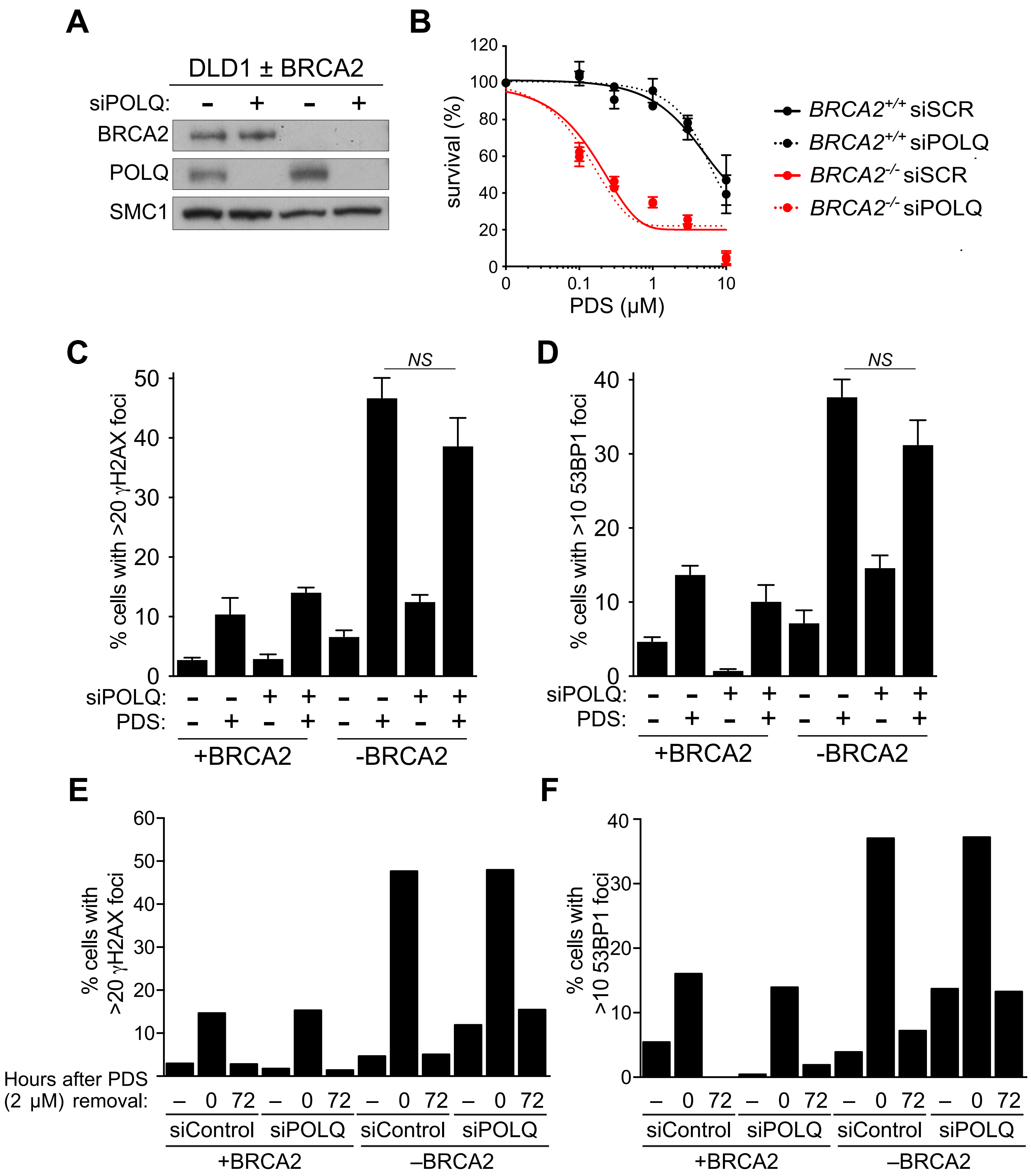
A
DLD1 ± BRCA2
siPOLQ: - + - +
BRCA2
POLQ
SMC1
B
survival (%)
PDS (μM)
BRCA2+/+ siSCR
BRCA2+/+ siPOLQ
BRCA2-/- siSCR
BRCA2-/- siPOLQ
C
% cells with >20 γH2AX foci
NS
siPOLQ: - - + + - - + +
PDS: - + - + - + - +
+BRCA2
-BRCA2
D
% cells with >10 53BP1 foci
NS
siPOLQ: - - + + - - + +
PDS: - + - + - + - +
+BRCA2
-BRCA2
E
% cells with >20 γH2AX foci
Hours after PDS (2 μM) removal: – 0 72 – 0 72 – 0 72 – 0 72
siControl siPOLQ siControl siPOLQ
+BRCA2
–BRCA2
F
% cells with >10 53BP1 foci
– 0 72 – 0 72 – 0 72 – 0 72
siControl siPOLQ siControl siPOLQ
+BRCA2
–BRCA2

Figure S5. Specificity of the DNA-PKcs inhibitor NU-7441.

A Dose-dependent viability assays of DNA-PKcs-proficient (*PRKDC* WT) or -deficient (*PRKDC KO*) human HAP1 cells treated with NU-7441 at the indicated concentrations for six days. Graphs and error bars represent average values and SEM obtained from $n = 3$ independent experiments, each performed in triplicate. Cells were immunoblotted as indicated. SMC1 was used as a loading control.

B Viability of DNA-PKcs-deficient (*PRKDC KO*) human HAP1 cells treated with NU-7441 at the indicated concentrations for six days. Graphs and error bars represent average values and SEM obtained from $n = 3$ independent experiments, each performed in triplicate. P values were calculated using an unpaired two-tailed t-test. **, $P \leq 0.01$; ***, $P \leq 0.001$; ****, $P \leq 0.0001$.

C Dose-dependent viability assays of DNA-PKcs-proficient (*PRKDC* WT) or -deficient (*PRKDC* KO) human HAP1 cells treated with pyridostatin at the indicated concentrations for six days. Graphs and error bars represent average values and SEM obtained from $n = 3$ independent experiments, each performed in triplicate.

D Dose-dependent viability assays of DNA-PKcs-proficient (*PRKDC* WT) or -deficient (*PRKDC* KO) human HAP1 cells treated with pyridostatin at the indicated concentrations, to which 0.25 μM NU-7441 was added, for six days. Graphs and error bars represent average values and SEM obtained from $n = 3$ independent experiments, each performed in triplicate. Light coloured lines represent values from (D).

Exact P values for (B) are provided in Appendix Table S8.

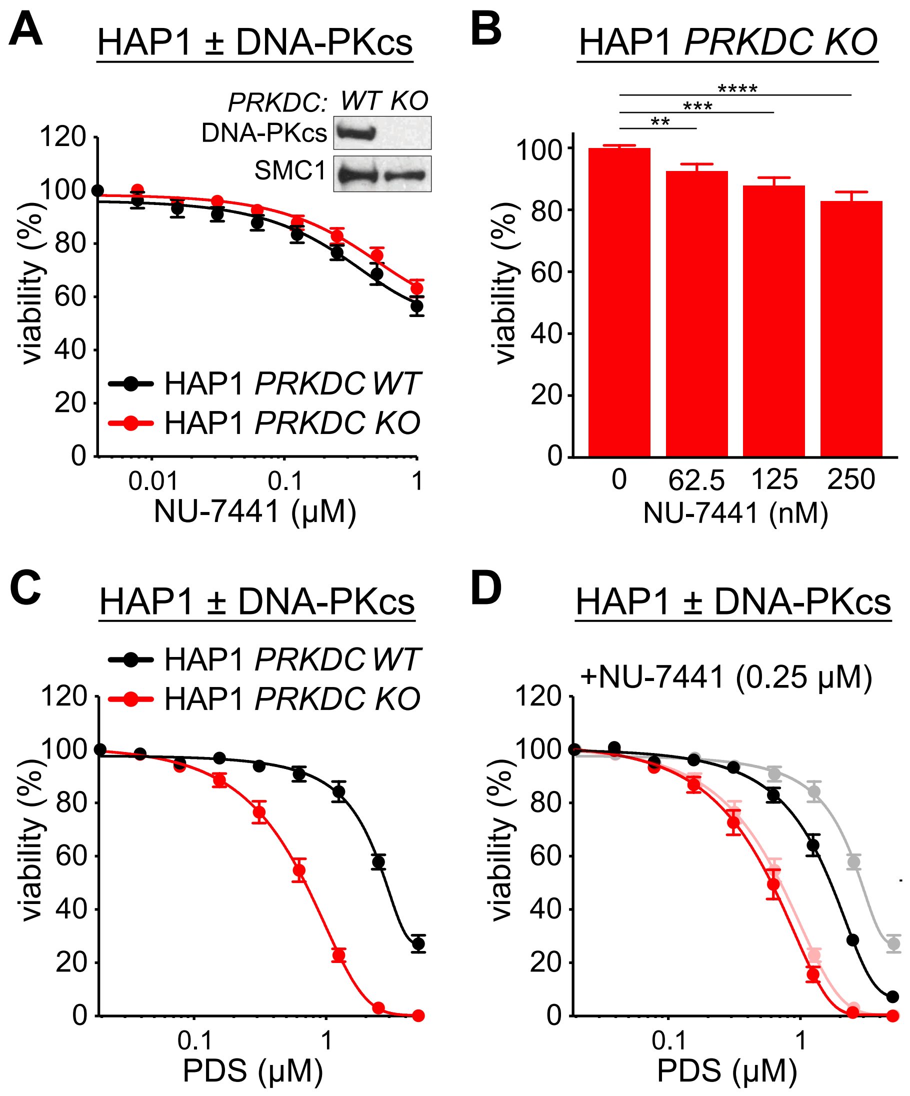
A
HAP1 ± DNA-PKcs
PRKDC: WT KO
DNA-PKcs
SMC1
viability (%)
HAP1 PRKDC WT
HAP1 PRKDC KO
NU-7441 (μM)
B
HAP1 PRKDC KO
****
***
**
viability (%)
0
62.5
125
250
NU-7441 (nM)
C
HAP1 ± DNA-PKcs
HAP1 PRKDC WT
HAP1 PRKDC KO
viability (%)
PDS (μM)
D
HAP1 ± DNA-PKcs
+NU-7441 (0.25 μM)
viability (%)
PDS (μM)

Appendix Figure S6. CRISPR/Cas9-mediated *BRCA2* knockout in RPE-1 cells.

A Dose-dependent viability assays of BRCA2-proficient (WT) or -deficient (clones 4D1 and 4D12) human RPE-1 cells treated with olaparib at the indicated concentrations for six days. Graphs and error bars represent average values and SEM obtained from $n = 3$ independent experiments, each performed in triplicate. Cells were immunoblotted as indicated. SMC1 was used as a loading control.

B BRCA2-proficient (WT) or -deficient (clones 4D1 and 4D12) human RPE-1 cells were untreated or exposed to 10 Gy ionising radiation and let to recover for 2 hours before being prepared for immunofluorescence with antibodies against γH2AX and RAD51. DNA was counterstained with DAPI. Scale bar represents 10 µm.

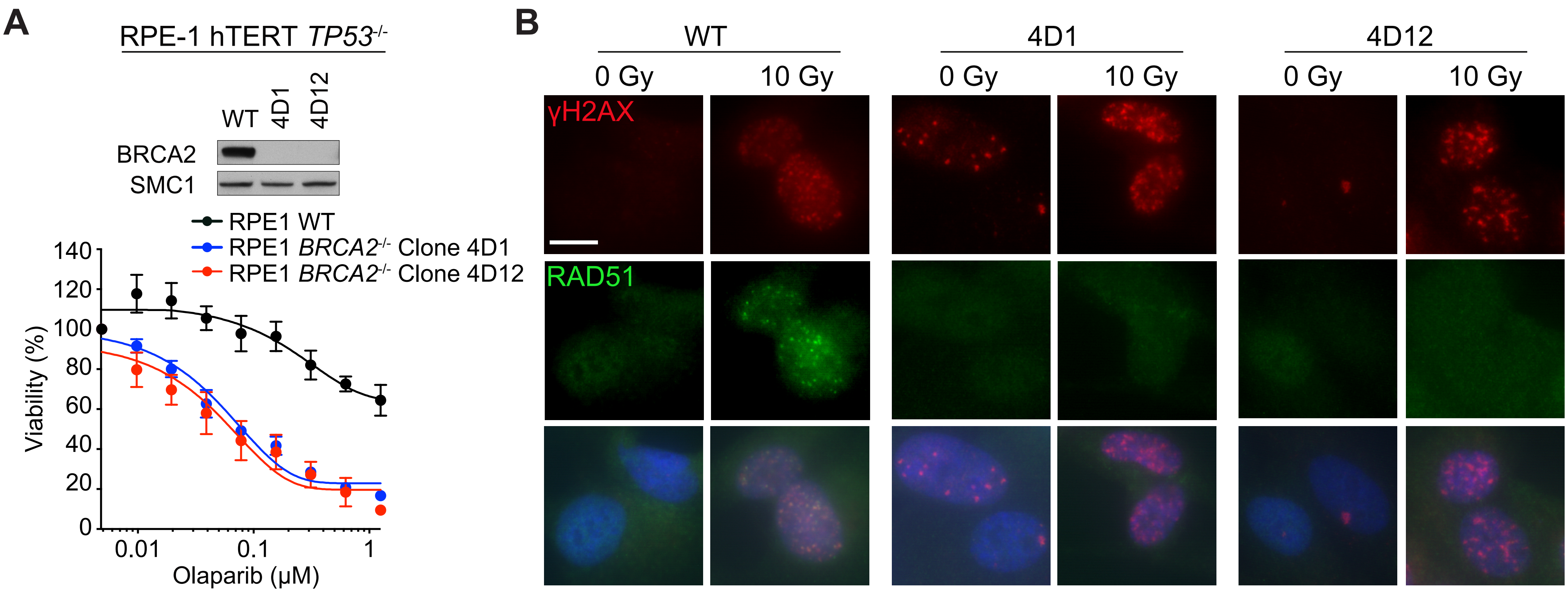
A
RPE-1 hTERT TP53-/-
WT
4D1
4D12
BRCA2
SMC1
RPE1 WT
RPE1 BRCA2-/- Clone 4D1
RPE1 BRCA2-/- Clone 4D12
Viability (%)
140
120
100
80
60
40
20
0
0.01
0.1
1
Olaparib (μM)
B
WT
4D1
4D12
0 Gy
10 Gy
0 Gy
10 Gy
0 Gy
10 Gy
γH2AX
RAD51

Appendix Figure S7. NHEJ abrogation in PARPi-resistant cells and the anti-proliferative effect of pyridostatin, NU-7441 and paclitaxel combinations *in vitro*.

A BRCA1-proficient KP3.33 ($Brca1^{+/+}$), BRCA1-deficient KB1PM5 ($Brca1^{-/-}$) and BRCA1/53BP1-deficient KB1PM5 ($Brca1^{-/-}/Tp53bp1^{-/-}$) mouse mammary tumour cells were treated with 2 μM pyridostatin (PDS) for 24 hours and released into fresh medium, before being subjected to cellular fractionation. Soluble (sol) and chromatin-bound (chromatin) fractions were immunoblotted as indicated. SMC1 and tubulin were used as loading controls for the chromatin-bound and soluble fractions, respectively.

B Whole-cell extracts prepared from cells treated as in (A) were immunoblotted as indicated. GAPDH was used as a loading control.

C Clonogenic survival assays of *BRCA1*-mutated (5396+1G>A) MDA-MB-436 cells treated with paclitaxel, NU-7441 or pyridostatin. Bar and dots represent the mean and individual values of $n = 2$ independent experiments, respectively.

D Dose-dependent clonogenic survival assays of *BRCA1*-mutated (5396+1G>A) MDA-MB-436 cells treated with paclitaxel at the indicated concentrations, to which 5 μM NU-7441, 0.3 μM pyridostatin (PDS) or combination of both was added. Dots represent the individual values of $n = 2$ independent experiments.

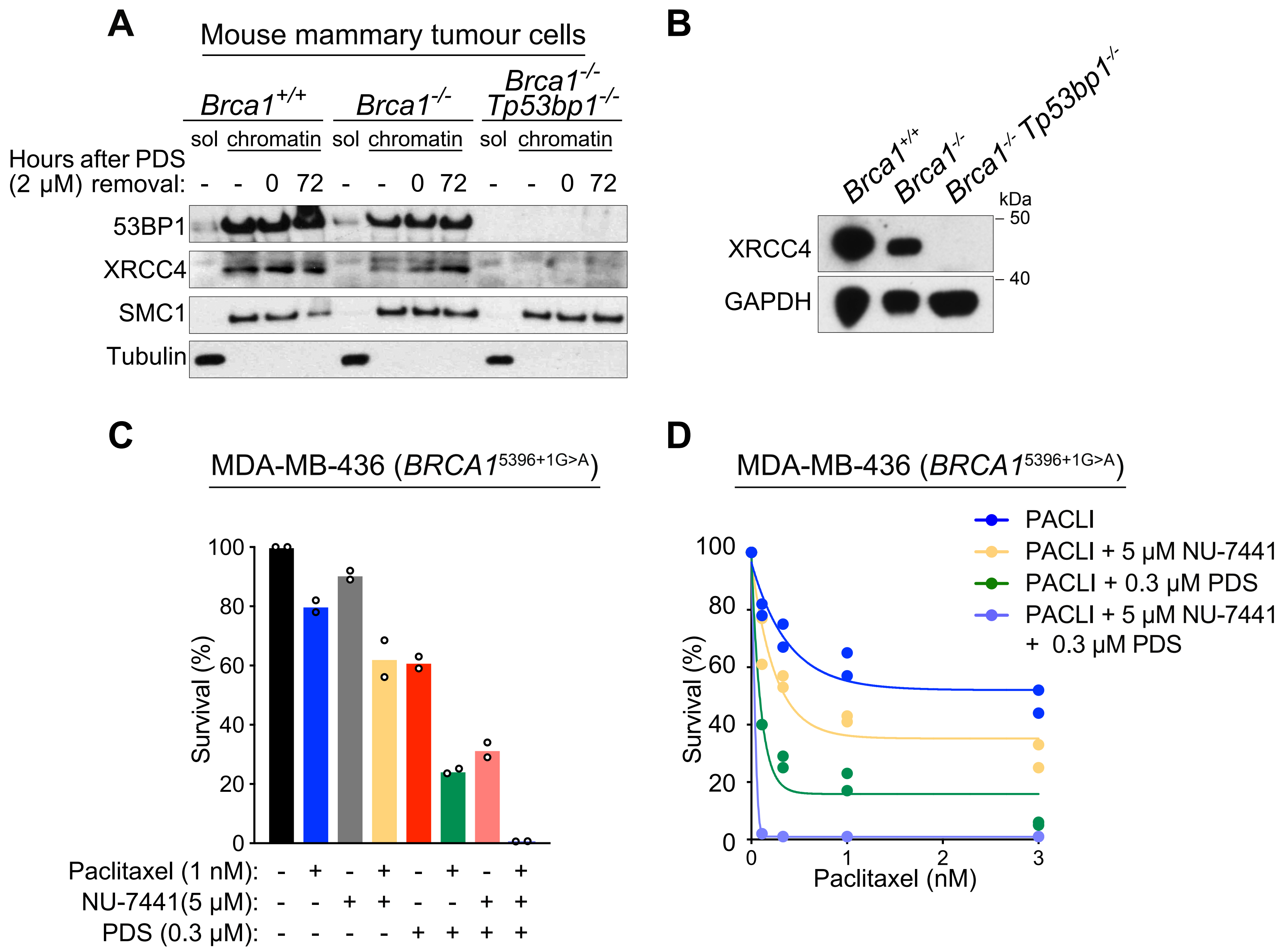
A
Mouse mammary tumour cells
Brca1+/+
Brca1-/-
Brca1-/- Tp53bp1-/-
sol chromatin sol chromatin sol chromatin
Hours after PDS (2 µM) removal: - - 0 72 - - 0 72 - - 0 72
53BP1
XRCC4
SMC1
Tubulin
B
Brca1+/+
Brca1-/-
Brca1-/- Tp53bp1-/-
kDa
50
40
XRCC4
GAPDH
C
MDA-MB-436 (BRCA15396+1G>A)
Survival (%)
100
80
60
40
20
0
Paclitaxel (1 nM): - + - + - + - +
NU-7441(5 µM): - - + + - - + +
PDS (0.3 µM): - - - - + + + +
D
MDA-MB-436 (BRCA15396+1G>A)
PACLI
PACLI + 5 µM NU-7441
PACLI + 0.3 µM PDS
PACLI + 5 µM NU-7441 + 0.3 µM PDS
Survival (%)
Paclitaxel (nM)
0
1
2
3

Appendix Figure S8. Effect of pyridostatin, NU-7441 and paclitaxel combinations in BRCA2-proficient HCT116 xenograft models.

BRCA2-proficient HCT116 human cells were injected intramuscularly in SCID male mice.

A Pyridostatin (PDS) was administered intravenously (*i.v.*; 7.5 mg/kg/day) and NU-7441 was administered intraperitoneally (*i.p.*; 10 mg/kg/day), over the indicated periods of time. Vertical dashed line indicates end of treatment. Tumour volume was measured at the timepoints shown on the graph and expressed relative to tumour volume at the beginning of treatment. Each experimental group included $n = 6$ mice. Error bars represent SEM.

B Pyridostatin (PDS) was administered intravenously (*i.v.*; 7.5 mg/kg/day) over the indicated periods of time, and paclitaxel (PACLI) was administered intravenously (*i.v.*; 20 mg/kg/day) on indicated single days. Vertical dashed line indicates end of treatment. Tumour volume was measured at the timepoints shown on the graph and expressed relative to tumour volume at the beginning of treatment. Each experimental group included $n = 6$ mice. Error bars represent SEM.

C Pyridostatin (PDS) was administered intravenously (*i.v.*; 7.5 mg/kg/day), NU-7441 was administered intraperitoneally (*i.p.*; 10 mg/kg/day), over the indicated periods of time, and paclitaxel (PACLI) was administered intravenously (*i.v.*; 20 mg/kg/day) on indicated single days. Vertical dashed line indicates end of treatment. Tumour volume was measured at the timepoints shown on the graph and expressed relative to tumour volume at the beginning of treatment. Each experimental group included $n = 6$ mice. Error bars represent SEM.

D Kaplan-Meier survival curve for mice treated as in (C), with each treatment group containing $n = 6$ mice. ***, $P \leq 0.001$; ****, $P \leq 0.0001$; *NS*, $P > 0.05$.

Exact *P* values for (A-C) are provided in Appendix Table S8 and (D) in Appendix Table S5.

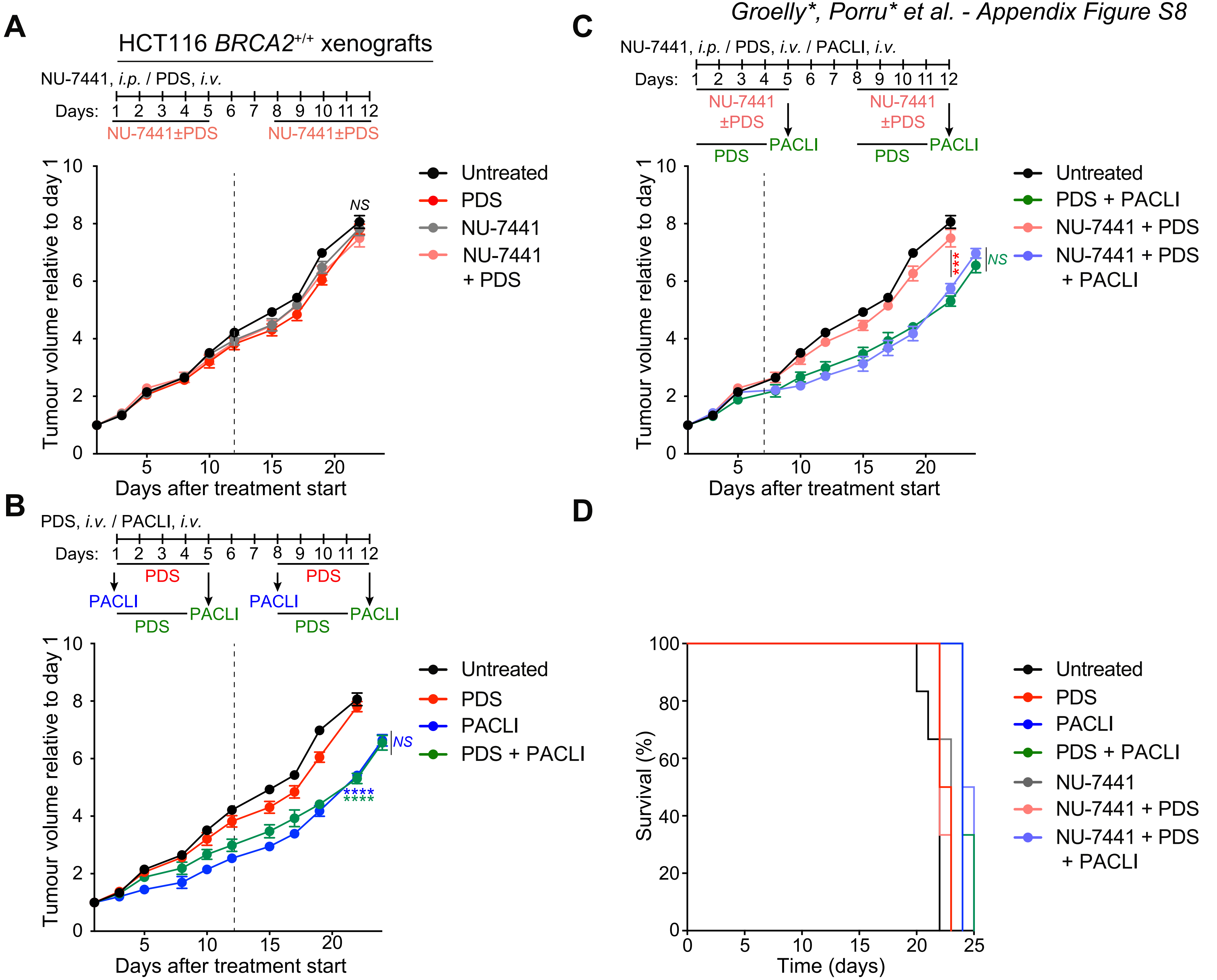
A
HCT116 BRCA2+/+ xenografts
NU-7441, i.p. / PDS, i.v.
Days: 1 2 3 4 5 6 7 8 9 10 11 12
NU-7441±PDS
NU-7441±PDS
Tumour volume relative to day 1
Days after treatment start
NS
Untreated
PDS
NU-7441
NU-7441 + PDS
B
PDS, i.v. / PACLI, i.v.
Days: 1 2 3 4 5 6 7 8 9 10 11 12
PDS
PACLI
PDS
PACLI
Tumour volume relative to day 1
Days after treatment start
NS
****
****
Untreated
PDS
PACLI
PDS + PACLI
C
NU-7441, i.p. / PDS, i.v. / PACLI, i.v.
Days: 1 2 3 4 5 6 7 8 9 10 11 12
NU-7441 ±PDS
PACLI
PDS
Tumour volume relative to day 1
Days after treatment start
***
NS
Untreated
PDS + PACLI
NU-7441 + PDS
NU-7441 + PDS + PACLI
D
Survival (%)
Time (days)
Untreated
PDS
PACLI
PDS + PACLI
NU-7441
NU-7441 + PDS
NU-7441 + PDS + PACLI

Appendix Table S1. ***In vivo*** **anti-tumour efficacy of pyridostatin and talazoparib on** ***BRCA2***$^{+/+}$ **and** ***BRCA2***$^{-/-}$ **DLD1 xenografts.**

CB17-SCID male mice were injected intramuscularly with $5x10^6$ cells per mouse. Tumours were allowed to grow to approximately 250 mm^3 before initiation of treatment (day 1). Mice were treated with pyridostatin (*i.v.*; 7.5 mg/kg/day) and talazoparib (*p.o.*; 0.33 mg/kg/day) for five consecutive days, followed by two-day break and five more days of treatment. Each experimental group included *n* = 5 mice. Tumour volume inhibition was calculated at the nadir of the effect using the formula: (1 - [tumour volume in treated mice] / [tumour volume in untreated mice]) x100 and expressed as average for *n* = 5 mice in each group. Tumour growth delay was calculated as the median time in days required for untreated and treated tumours to reach 300 mm^3. Stable disease was defined as mice in which tumour volume did not change for at least two weeks after initiation of treatment. Body weight loss is reported as weight at the end of treatment relative to the first day of treatment (%), as average for *n* = 5 mice in each group.

Treatment	Tumour volume inhibition (%)	Tumour growth delay (days)	Stable disease	Body weight loss (%)	Toxic deaths
BRCA2$^{+/+}$ **DLD1 Pyridostatin**	8	0	0/5	0	0/5
BRCA2$^{-/-}$ **DLD1 Pyridostatin**	66	10	2/5	0	0/5
BRCA2$^{+/+}$ **DLD1 Talazoparib**	19	0	0/5	0	0/5
BRCA2$^{-/-}$ **DLD1 Talazoparib**	53	8	1/5	2	0/5

Appendix Table S2. *In vivo* anti-tumour efficacy of pyridostatin on *BRCA2*$^{+/+}$ and *BRCA2*$^{-/-}$ HCT116 xenografts.

CB17-SCID male mice were injected intramuscularly with 5x10^{6} cells per mouse. Tumours were allowed to grow to approximately 250 mm^{3} before initiation of treatment (day 1). Mice were treated with pyridostatin (*i.v.*; 7.5 mg/kg/day) for five consecutive days, followed by two-day break and five more days of treatment. Each experimental group included *n* = 5 mice. Tumour volume inhibition was calculated at the nadir of the effect using the formula: (1 - [tumour volume in treated mice] / [tumour volume in untreated mice]) x100 and expressed as average for *n* = 5 mice in each group. Tumour growth delay was calculated as the median time in days required for untreated and treated tumours to reach 500 mm^{3}. Stable disease was defined as mice in which tumour volume did not change for at least two weeks after initiation of treatment. Body weight loss is reported as weight at the end of treatment relative to the first day of treatment (%), as average for *n* = 5 mice in each group.

Treatment	Tumour volume inhibition (%)	Tumour growth delay (days)	Stable disease	Body weight loss (%)	Toxic deaths
***BRCA2*$^{+/+}$ HCT116 Pyridostatin**	18	1	0/5	0	0/5
***BRCA2*$^{-/-}$ HCT116 Pyridostatin**	59	9	1/5	0	0/5

Appendix Table S3. Statistical analysis of the survival advantage conferred by pyridostatin, NU-7441, paclitaxel and their combination on BRCA1-deficient MDA-MB-436 xenografts.

Statistical significance of Kaplan-Meier survival curves (Fig. 5D) was assessed between the indicated treated groups by log-rank test.

Treatments	**Untreated**	**PDS**	**PACLI**	**PDS + PACLI**	**NU-7441**	**NU-7441 + PDS**	**NU-7441 + PDS + PACLI**
**Untreated**	-	$P = 0.0015$	$P = 0.0015$	$P = 0.0015$	$P = 0.0309$	$P = 0.0015$	$P = 0.0015$
**PDS**		-	$P = 0.3904$	$P = 0.0019$	$P = 0.0018$	$P = 0.0019$	$P = 0.0019$
**PACLI**			-	$P = 0.0027$	$P = 0.0018$	$P = 0.0027$	$P = 0.0027$
**PDS + PACLI**				-	$P = 0.0018$	$P = 0.1048$	$P = 0.0018$
**NU-7441**					-	$P = 0.0018$	$P = 0.0018$
**NU-7441 + PDS**						-	$P = 0.0018$
**NU-7441 + PDS + PACLI**							-

Appendix Table S4. *In vivo* anti-tumour efficacy of pyridostatin, NU-7441, paclitaxel and their combination on HCT116 *BRCA2*$^{+/+}$ xenografts.
CB17-SCID male mice were injected intramuscularly with 5x10^6 cells per mouse. Tumours were allowed grow for 6 days to approximately 220 mm^3 before initiation of treatment (day 1). Mice were treated with pyridostatin (*i.v.*; 7.5 mg/kg/day) at days 1-5 and 8-12 and NU-7441 (*i.p.*; 10 mg/kg/day) at days 1-5 and 8-12, two hours before injection of pyridostatin. Paclitaxel (*i.v.*; 20 mg/kg) was administered at day 1 and 8 and at day 5 and 12, for the double and triple combinations (Appendix Fig S8). Each experimental group included *n* = 6 mice. Tumour volume inhibition was calculated at the nadir of the effect using the formula: (1 - [tumour volume in treated mice] / [tumour volume in untreated mice]) x100 and expressed as average for *n* = 6 mice in each group. Stable disease was defined as mice in which tumour volume did not change for at least two weeks after initiation of treatment. Partial or complete responses were defined as mice in which ≥50% reduction of tumour volume or tumour disappearance, respectively, were observed for at least two weeks after initiation of treatment. Increase in survival was calculated by comparing median survival of treated relative to untreated mice (%). Body weight loss is reported as weight at the end of treatment relative to the first day of treatment (%), as average for *n* = 5 mice in each group.

*Animals were euthanized for ethical reasons when tumours reached a mean of 1.7 to 2 cm^3 in volume.

Treatment	Tumour volume inhibition (%)	Stable disease	Partial response	Complete response	Increase in survival (%)*	Body weight loss (%)	Toxic deaths
**NU-7441**	12	0/6	0/6	0/6	-	2	0/6
**Pyridostatin**	17	0/6	0/6	0/6	-	4	0/6
**Paclitaxel**	41	0/6	0/6	0/6	9	6	0/6
**Pyridostatin +NU-7441**	17	0/6	0/6	0/6	-	4	0/6
**Pyridostatin + Paclitaxel**	36	0/6	0/6	0/6	9	7	0/6
**Pyridostatin + NU-7441 + Paclitaxel**	43	0/6	0/6	0/6	9	7	0/6

Appendix Table S5. Statistical analysis of the survival advantage conferred by pyridostatin, NU-7441, paclitaxel and their combination on HCT116 *BRCA2*$^{+/+}$ xenografts.

Statistical significance of Kaplan-Meier survival curves (Appendix Fig S8D) was assessed between the indicated treated groups by log-rank test.

Treatments	**Untreated**	**PDS**	**PACLI**	**PDS + PACLI**	**NU-7441**	**NU-7441 + PDS**	**NU-7441 + PDS + PACLI**
**Untreated**	-	*P* = 0.0290	*P* = 0.0012	*P* = 0.0012	*P* = 0.0131	*P* = 0.0550	*P* = 0.0012
**PDS**		-	*P* = 0.0009	*P* = 0.0009	*P* = 0.5751	*P* = 0.5751	*P* = 0.0009
**PACLI**			-	*P* = 0.1380	*P* = 0.0012	*P* = 0.0006	*P* = 0.0555
**PDS + PACLI**				-	*P* = 0.0012	*P* = 0.0006	*P* = 0.5751
**NU-7441**					-	*P* = 0.2689	*P* = 0.0012
**NU-7441 + PDS**						-	*P* = 0.0006
**NU-7441 + PDS + PACLI**							-

Appendix Table S6. Statistical analysis of the survival advantage conferred by pyridostatin, NU-7441, paclitaxel and their combination on HCT116 *BRCA2$^{-/-}$* xenografts.
Statistical significance of Kaplan-Meier survival curves (Fig 6D) was assessed between the indicated treated groups by log-rank test.

Treatments	**Untreated**	**PDS**	**PACLI**	**PDS + PACLI**	**NU-7441**	**NU-7441 + PDS**	**NU-7441 + PDS + PACLI**
**Untreated**	-	P = 0.0009	P = 0.0009	P = 0.0009	P = 0.0009	P = 0.0009	P = 0.0009
**PDS**		-	P = 0.1808	P = 0.0255	P = 0.0009	P = 0.6356	P = 0.0009
**PACLI**			-	P = 0.0141	P = 0.0190	P = 0.2841	P = 0.0012
**PDS + PACLI**				-	P = 0.051	P = 0.0095	P = 0.0012
**NU-7441**					-	P = 0.0555	P = 0.0009
**NU-7441 + PDS**						-	P = 0.0009
**NU-7441 + PDS + PACLI**							-

Appendix Table S7. ***In vivo*** **anti-tumour efficacy of pyridostatin and CX-5461 on** ***BRCA2***$^{-/-}$ **DLD1 and** ***BRCA2***$^{-/-}$ **HCT116 xenografts.**

CB17-SCID male mice were were injected intramuscularly with $5x10^6$ cells per mouse. Tumours were allowed grow to approximately 250 mm^3 before initiation of treatment (day 1). Mice were treated with with pyridostatin (*i.v.*; 7.5 mg/kg/day) for five consecutive days, followed by two-day break and five more days of treatment or with CX-5461(*o.s.*; 50 mg/kg/day) three times, once every three days. Each experimental group included n = 5 mice. Tumour weight inhibition was calculated at the nadir of the effect using the formula: (1 - [tumour weight in treated mice] / [tumour weight in untreated mice]) x100 and expressed as average for n = 5 mice in each group. Tumour growth delay was calculated as the median time in days required for untreated and treated tumours to reach the same size. Stable disease was defined as mice in which tumour volume did not change for at least two weeks after initiation of treatment. Body weight loss is reported as weight at the end of treatment relative to the first day of treatment (%), as average for n = 5 mice in each group.

Treatment	Tumour weight inhibition (%)	Tumour growth delay (days)	Stable disease	Body weight loss (%)	Toxic deaths
BRCA2$^{-/-}$ **DLD1 Pyridostatin**	66	10	2/5	0	0/5
BRCA2$^{-/-}$ **DLD1 CX-5461**	46	6	0/5	25	2/5
BRCA2$^{-/-}$ **HCT116 Pyridostatin**	59	9	1/5	0	0/5
BRCA2$^{-/-}$ **HCT116 CX-5461**	47	14	0/5	24	1/5

Appendix Table S8. Exact *P* values from figures.

	Treatments	**P value (*t-test*)**
Figure 1A<br>**DLD1 *BRCA2*$^{+/+}$**	PDS vs Untreated	0.54
	Talazoparib vs Untreated	0.09
Figure 1B<br>**DLD1 *BRCA2*$^{-/-}$**	PDS vs Untreated	0.00033
	Talazoparib vs Untreated	0.00039
Figure 1D<br>**DLD1 *BRCA2*$^{-/-}$**	0 hours after PDS vs Untreated	0.0107
	72 hours after PDS vs Untreated	0.8948
Figure 1E<br>**DLD1 *BRCA2*$^{-/-}$**	0 hours after PDS vs Untreated	0.0002
Figure 2C<br>**H1299+shBRCA2^{DOX}<br>-BRCA2**	*IFIT1* PDS (10 µM) vs Untreated	0.0004
	IFIT2 PDS (10 µM) vs Untreated	0.0161
	ISG15 PDS (10 µM) vs Untreated	0.0001

Figure 2F<br>**H1299+shBRCA2^{DOX}<br>+BRCA2**	Untreated Day 3 vs Day 1	0.8964
	PDS Day 3 vs Day 1	0.0329
**-BRCA2**	Untreated Day 3 vs Day 1	0.0017
	PDS Day 3 vs Day 1	0.0066
	PDS Day 1 vs Untreated Day 1	0.0112
	PDS Day 3 vs Untreated Day 3	0.0123
Figure 3A<br>**KP3.33 *Brca1*$^{+/+}$**	PDS vs Untreated	0.4
	Talazoparib vs Untreated	0.023
Figure 3B<br>**KB1PM5 *Brca1*$^{-/-}$<br>*Tp53bp1*$^{-/-}$**	PDS vs Untreated	0.0001
	Talazoparib vs Untreated	0.25
Figure 4B<br>**VHIO179 PDTX**	PDS vs Untreated	5.8E-7

Figure 5A **MDA-MB-436**	PDS vs Untreated	0.0018
	NU-7441 vs Untreated	0.004
	PDS vs NU-7441+PDS	0.00059
	NU vs NU-7441+PDS	0.000006
	NU-7441+PDS vs Untreated	0.000014
Figure 5B **MDA-MB-436**	Paclitaxel vs Untreated	0.00042
	PDS vs PDS+Paclitaxel	0.000061
	Paclitaxel vs PDS+Paclitaxel	0.000067
	PDS+Paclitaxel vs Untreated	0.000004
Figure 5C **MDA-MB-436**	NU-7441+PDS vs NU-7441+PDS+Paclitaxel	0.000005
	PDS+Paclitaxel vs NU-7441+PDS+Paclitaxel	5.14E-8
Figure 6A **HCT116** ***BRCA2*** $^{-/-}$	PDS vs Untreated	0.000003

	NU-7441 vs Untreated	0.64
	PDS vs NU-7441+PDS	0.001
	NU vs NU-7441+PDS	7.8E-7
	NU-7441+PDS vs Untreated	1.15E-8
Figure 6B **HCT116** ***BRCA2*** $^{-/-}$	Paclitaxel vs Untreated	0.000017
	PDS vs PDS+Paclitaxel	0.000038
	Paclitaxel vs PDS+Paclitaxel	3.3E-6
	PDS+Paclitaxel vs Untreated	1.9E-9
Figure 6C **HCT116** ***BRCA2*** $^{-/-}$	NU-7441+PDS vs NU-7441+PDS+Paclitaxel	6.2E-6
	PDS+Paclitaxel vs NU-7441+PDS+Paclitaxel	4.68E-7
Appendix Figure S1A **HCT116** ***BRCA2*** $^{+/+}$	PDS vs Untreated	0.71
Appendix Figure S1B **HCT116** ***BRCA2*** $^{-/-}$	PDS vs Untreated	0.000024

Appendix Figure S1F **HCT116**	Untreated *BRCA2*$^{-/-}$ vs Untreated *BRCA2*$^{+/+}$	0.3399
	PDS BRCA2$^{-/-}$ vs PDS BRCA2$^{+/+}$	0.0001
Appendix Figure S2A **DLD1** **-BRCA2**	PDS vs Untreated	0.0001
	HU vs Untreated	0.0001
	HU+Mirin vs HU	0.0001
	PDS+Mirin vs PDS	0.0001
Appendix Figure S2C **DLD1** **+BRCA2**	0 hours after PDS removal vs Untreated	0.4308
**-BRCA2**	0 hours after PDS removal vs Untreated	0.0001
	72 hours after PDS removal vs Untreated	0.0736
Appendix Figure S3C **DLD1** **-BRCA2**	0 hours after PDS removal vs Untreated	0.0002
	72 hours after PDS removal vs Untreated	0.003
	72 vs 0 hours after PDS removal	0.0004

Appendix Figure S3D<br>**DLD1**<br>**+BRCA2**	0 hours after PDS removal vs Untreated	0.3363
**-BRCA2**	0 hours after PDS removal vs Untreated	0.0099
	72 hours after PDS removal vs Untreated	0.4422
Appendix Figure S4C<br>**DLD1**<br>**-BRCA2**	siPOLQ+PDS vs PDS	0.2336
Appendix Figure S4D<br>**DLD1**<br>**-BRCA2**	siPOLQ+PDS vs PDS	0.1805
Appendix Figure S5B<br>**HAP1**<br>***PRKDC KO***	65.2 nM vs 0 nM NU-7441	0.0065
	125 nM vs 0 nM NU-7441	0.0004
	250 nM vs 0 nM NU-7441	0.0001
Appendix Figure S8A<br>**HCT116**<br>***BRCA2*** $^{+/+}$	PDS vs Untreated	0.38
	NU-7441 vs Untreated	0.51
	PDS vs NU-7441+PDS	0.4

	NU-7441 vs NU-7441+PDS	0.4
	NU-7441+PDS vs Untreated	0.16
Appendix Figure S8B **HCT116** ***BRCA2***$^{+/+}$	Paclitaxel vs Untreated	0.000001
	PDS vs PDS+Paclitaxel	0.000002
	Paclitaxel vs PDS+Paclitaxel	0.64
	PDS+Paclitaxel vs Untreated	0.000002
Appendix Figure S8C **HCT116** ***BRCA2***$^{+/+}$	PDS+Paclitaxel vs NU-7441+PDS	0.000099
	NU-7441+PDS vs NU-7441+PDS+Paclitaxel	0.0005
	PDS+Paclitaxel vs NU-7441+PDS+Paclitaxel	0.1
