# Supplementary figures and images for: Anti‐tumoural activity of the G‐quadruplex ligand pyridostatin against BRCA1/2‐deficient tumours
Source: EMBO Mol Med. 2022 Feb 2;14(3):e14501. doi: 10.15252/emmm.202114501 (PMC8899905; doi:10.15252/emmm.202114501)

Appendix Fig S3A

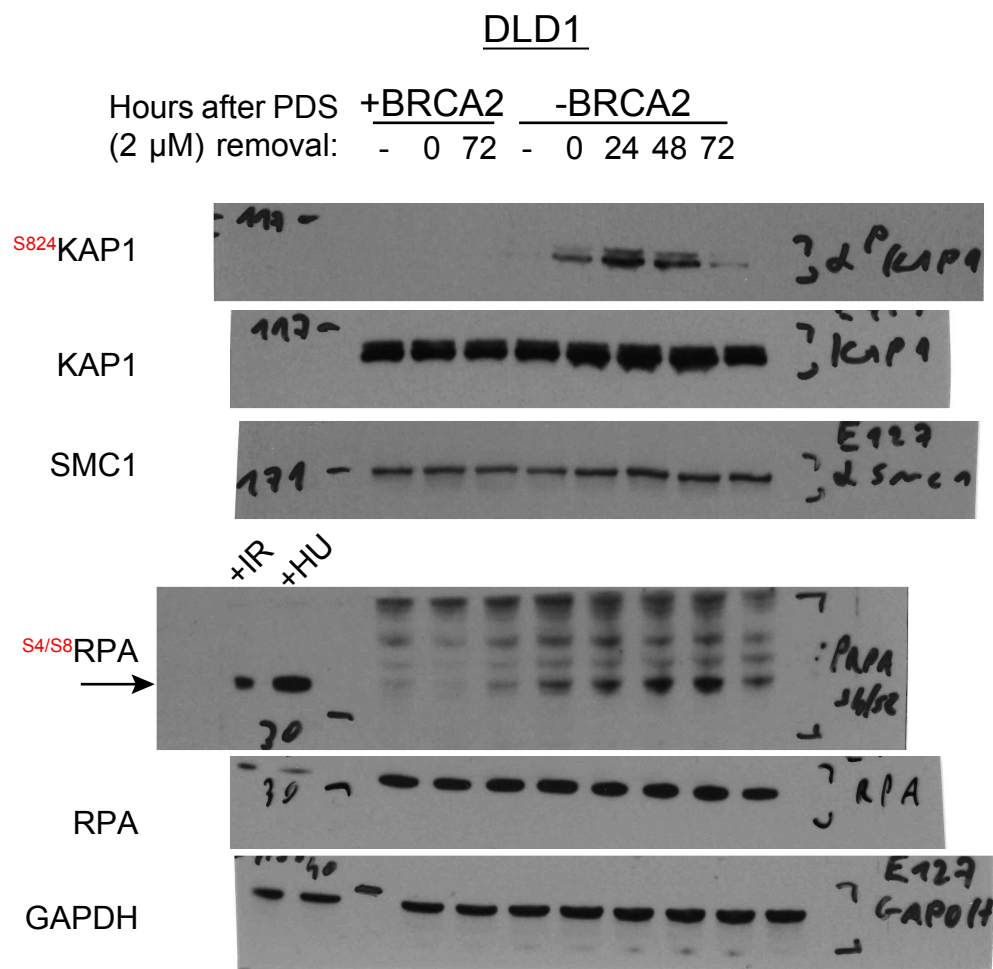

# Appendix Fig S7A

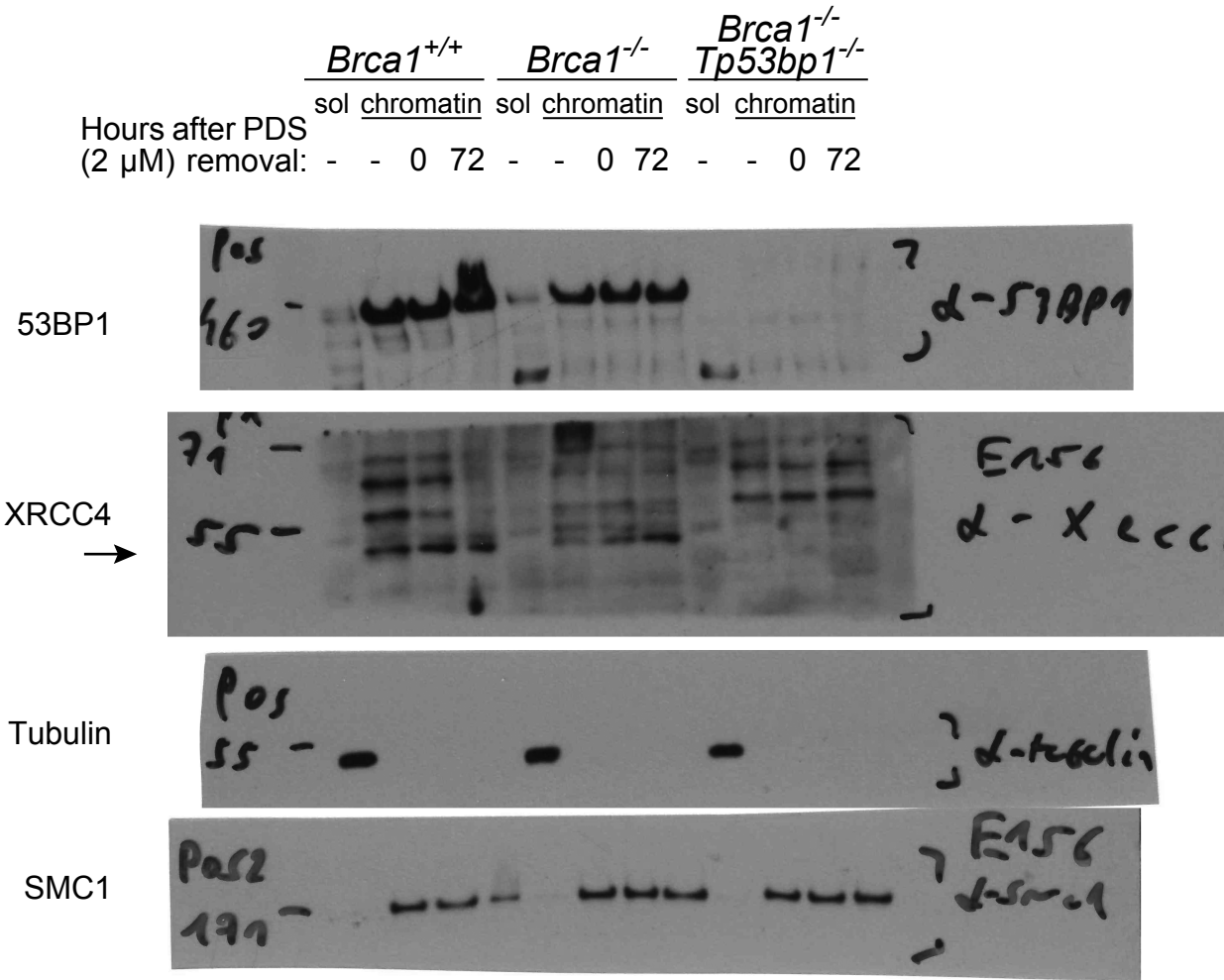

Supplement: Supplementary file 2 — Source Data for Appendix [file EMMM-14-e14501-s001.zip › emmm202114501-sup-0005-SDataAppendixFigs.pdf]

Fig 1F

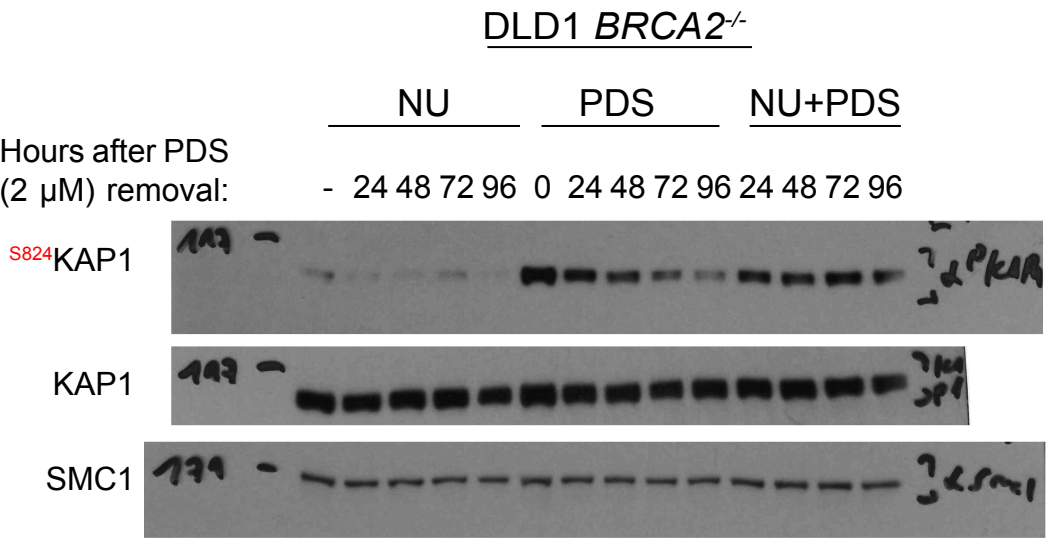

Supplement: Supplementary file 3 — Source Data for Figure 1 [file EMMM-14-e14501-s005.pdf]

Fig 2A

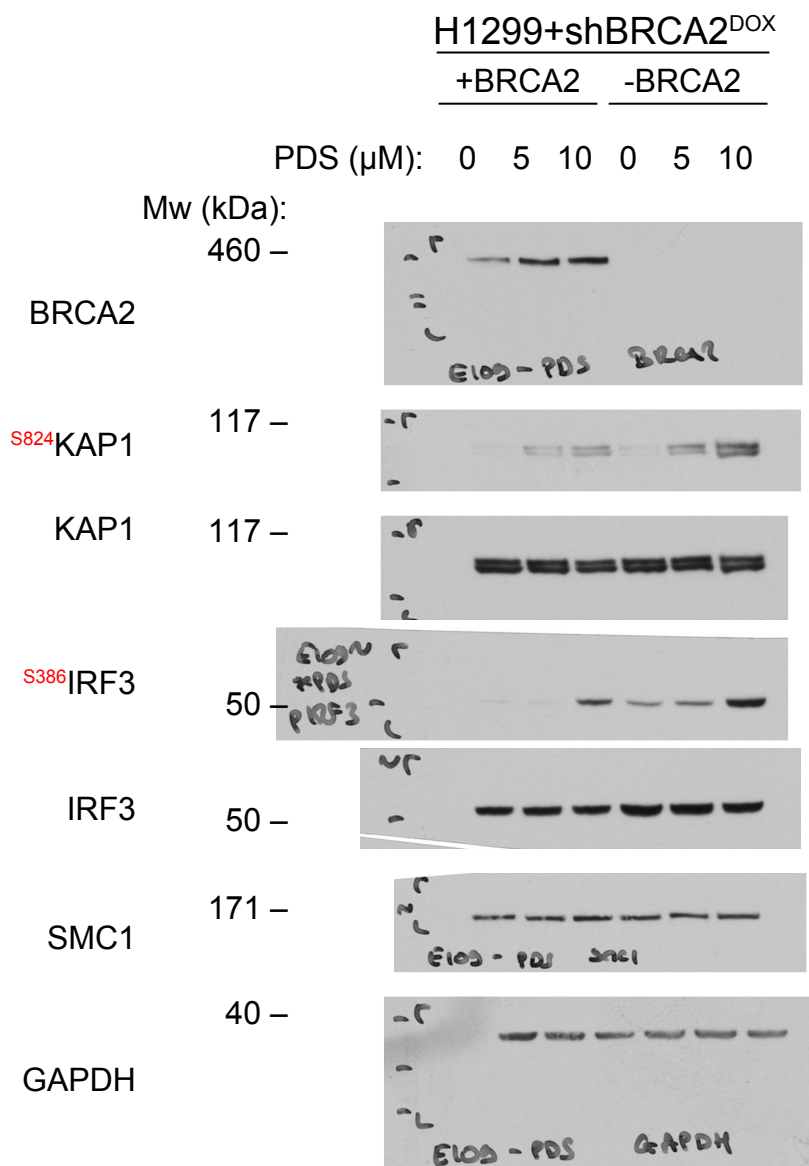

Fig 2B

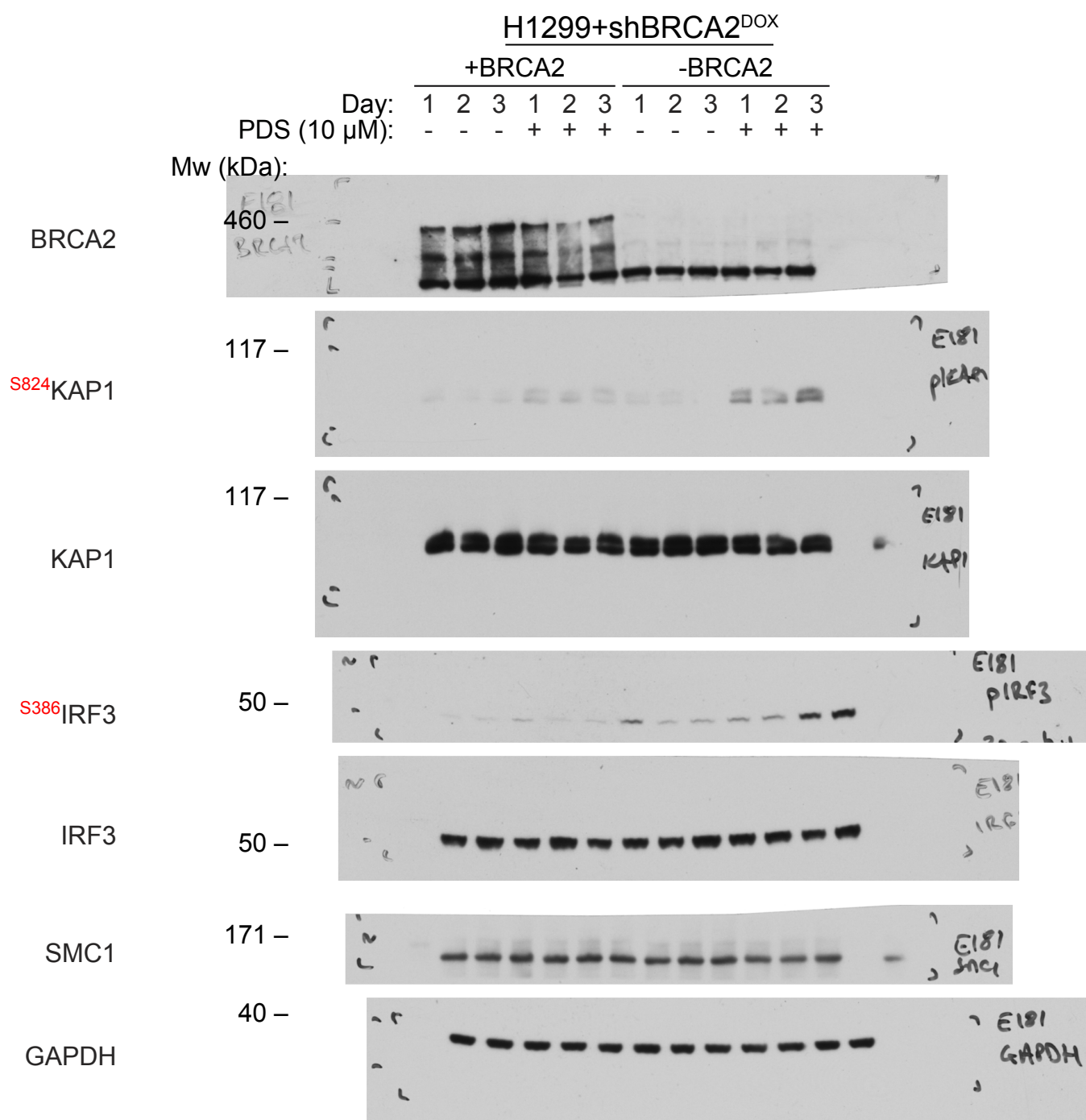

Fig 2D

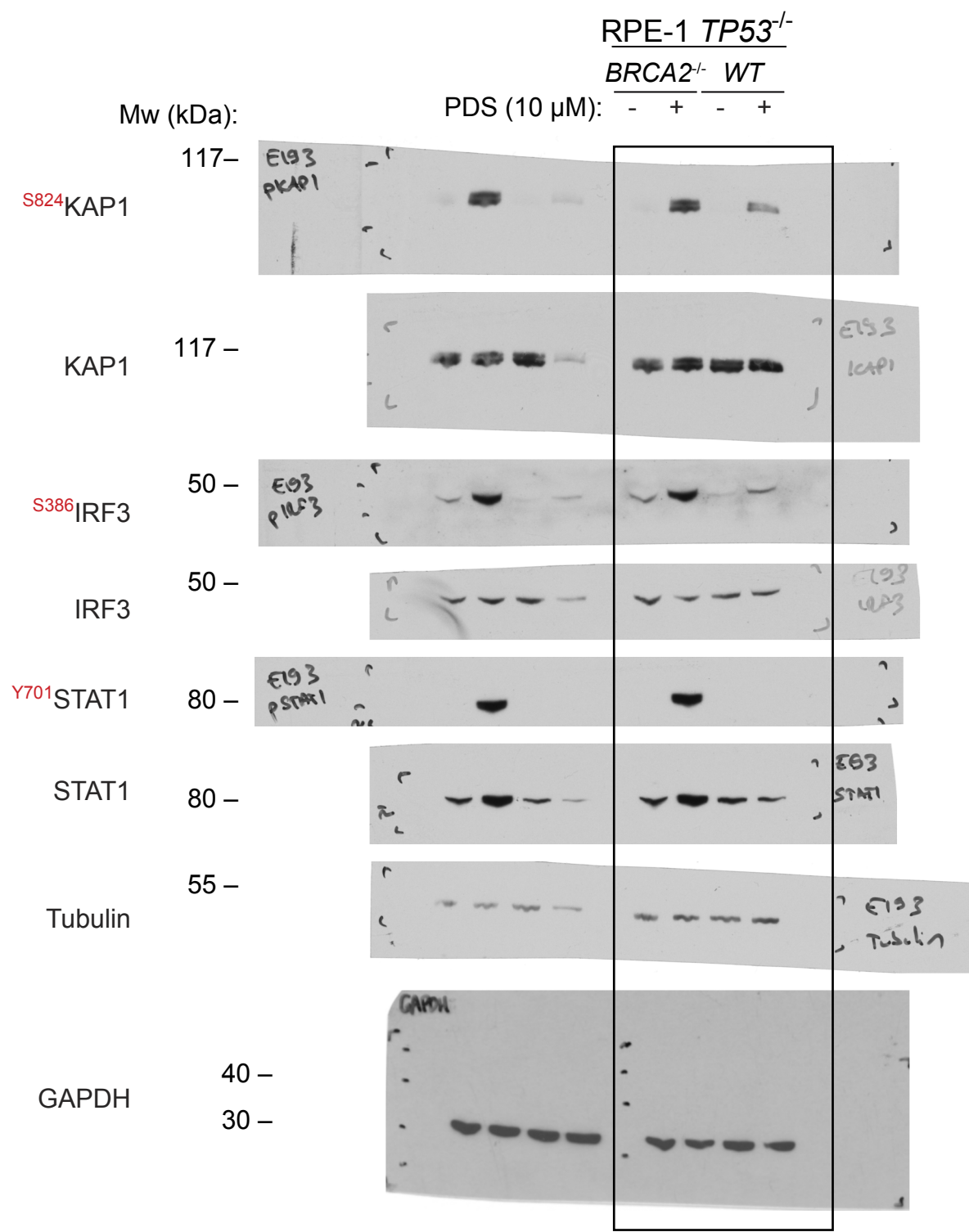

Supplement: Supplementary file 4 — Source Data for Figure 2 [file EMMM-14-e14501-s002.pdf]

Fig 3C

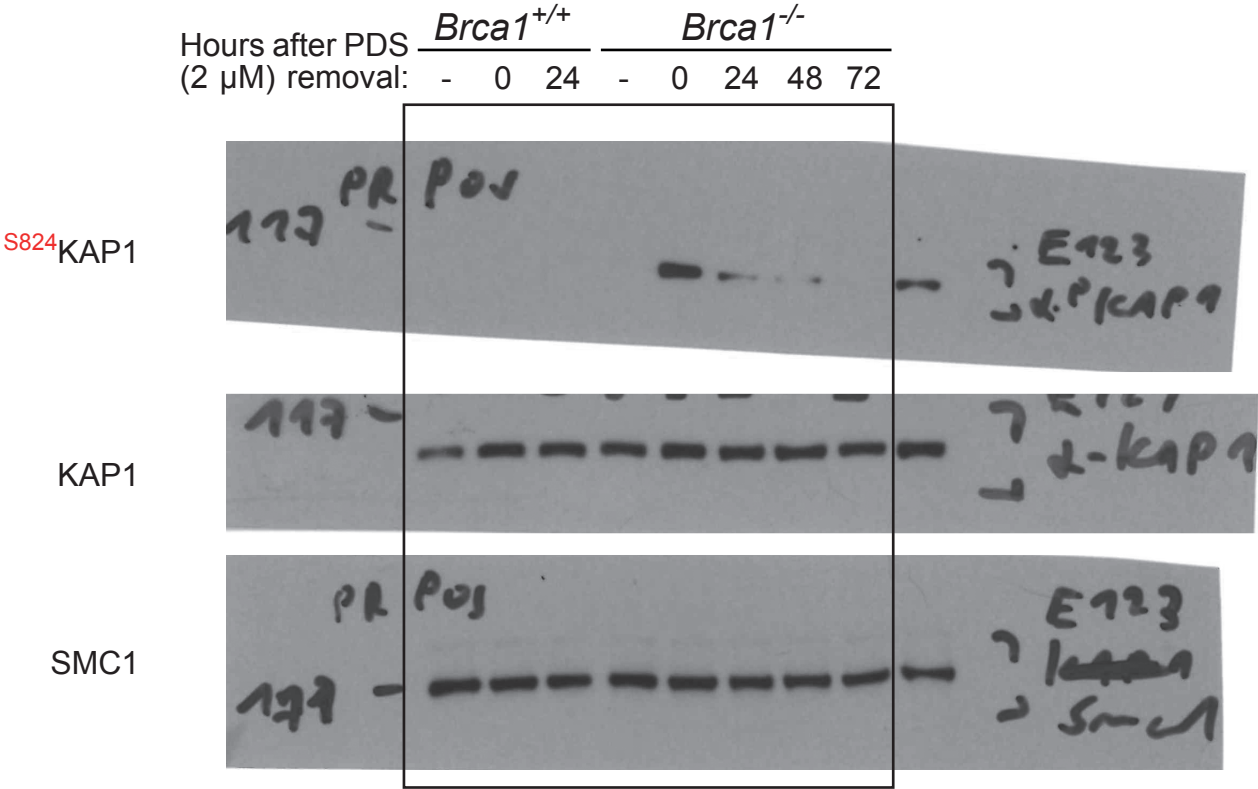

Fig 3D

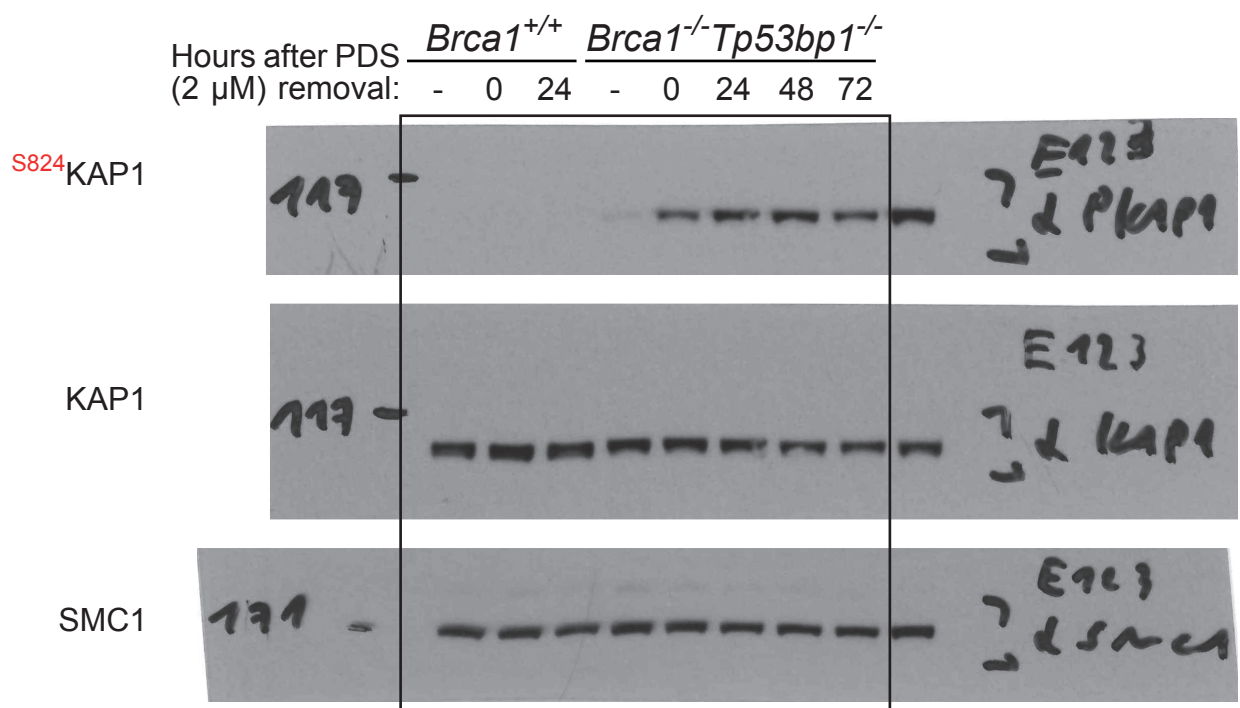

Fig 3E

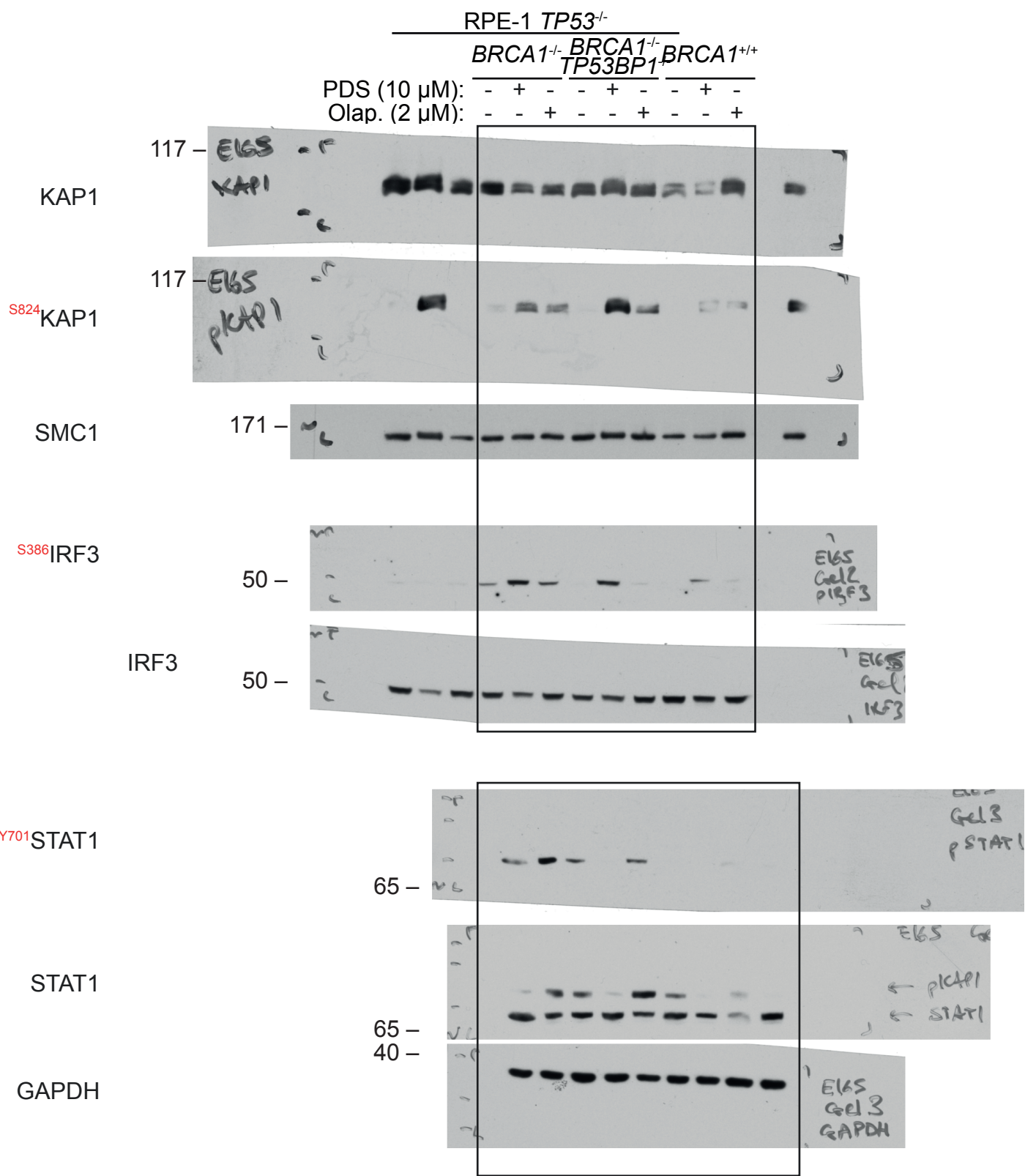

Supplement: Supplementary file 5 — Source Data for Figure 3 [file EMMM-14-e14501-s003.pdf]
