# Supplementary material for: Anti‐tumoural activity of the G‐quadruplex ligand pyridostatin against BRCA1/2‐deficient tumours
Source: EMBO Mol Med. 2022 Feb 2;14(3):e14501. doi: 10.15252/emmm.202114501 (PMC8899905; doi:10.15252/emmm.202114501)
Supplement: Supplementary file 6 [file EMMM-14-e14501-s006.pdf]

EMBO Molecular Medicine

Anti-tumoral activity of the G-quadruplex ligand pyridostatin against BRCA1/2-deficient tumours

Florian Groelly, Manuela Porru, Jutta Zimmer, Hugo Benainous, Yanti De Visser, Anastasiya Kosova, Serena Di Vito, Violeta Serra, Anderson Ryan, Carlo Leonetti, Alejandra Bruna, Annamaria Biroccio, and Madalena Tarsounas
DOI: 10.15252/emmm.202114501

Corresponding author(s): Madalena Tarsounas (madalena.tarsounas@oncology.ox.ac.uk) , Annamaria Biroccio (annamaria.biroccio@ifo.gov.it)

Review Timeline:

Submission Date:	6th May 21
Editorial Decision:	9th Jun 21
Revision Received:	23rd Sep 21
Editorial Decision:	29th Oct 21
Revision Received:	15th Nov 21
Editorial Decision:	30th Nov 21
Revision Received:	20th Dec 21
Accepted:	7th Jan 22

Editor: Lise Roth

Transaction Report:

(Note: With the exception of the correction of typographical or spelling errors that could be a source of ambiguity, letters and reports are not edited. Depending on transfer agreements, referee reports obtained elsewhere may or may not be included in this compilation. Referee reports are anonymous unless the Referee chooses to sign their reports.)

9th Jun 2021

Dear Madalena,

Thank you for the submission of your manuscript to EMBO Molecular Medicine, and please accept my apologies for the delay in getting back to you, as one referee needed more time to complete his/her review. We have now received feedback from the three reviewers who agreed to evaluate your manuscript.

As you will see from the reports below, while referee #3 is overall supportive of publication of the manuscript pending minor revisions, referees #1 and #2 raise serious concerns pertaining both to the limited novelty and to the unsupported mechanism. Referee #1 suggested to turn the manuscript into a short report, that would focus on the activation of the innate immune signaling mechanism and on the novel drug combinations.
We would therefore like to invite you to revise the manuscript along these lines, and strengthen the identified mechanism on innate immunity signaling, as well as better define the novelty of the present manuscript in light of previous publications.

Acceptance of the manuscript will entail a second round of review. EMBO Molecular Medicine encourages a single round of revision only and therefore, acceptance or rejection of the manuscript will depend on the completeness of your responses included in the next, final version of the manuscript. For this reason, and to save you from any frustrations in the end, I would strongly advise against returning an incomplete revision.

Revised manuscripts should be submitted within three months of a request for revision; they will otherwise be treated as new submissions, except under exceptional circumstances in which a short extension is obtained from the editor.

When submitting your revised manuscript, please carefully review the instructions that follow below. We perform an initial quality control of all revised manuscripts before re-review; failure to include requested items will delay the evaluation of your revision.

We require:

1) A .docx formatted version of the manuscript text (including legends for main figures, EV figures and tables). Please make sure that the changes are highlighted to be clearly visible.

2) Individual production quality figure files as .eps, .tif, .jpg (one file per figure). For guidance, download the 'Figure Guide PDF' (https://www.embopress.org/page/journal/17574684/authorguide#figureformat).

3) A .docx formatted letter INCLUDING the reviewers' reports and your detailed point-by-point responses to their comments. As part of the EMBO Press transparent editorial process, the point-by-point response is part of the Review Process File (RPF), which will be published alongside your paper.

4) A complete author checklist, which you can download from our author guidelines (https://www.embopress.org/page/journal/17574684/authorguide#submissionofrevisions). Please insert information in the checklist that is also reflected in the manuscript. The completed author checklist will also be part of the RPF.

5) It is mandatory to include a 'Data Availability' section after the Materials and Methods. Before submitting your revision, primary datasets produced in this study need to be deposited in an appropriate public database, and the accession numbers and database listed under 'Data Availability'. Please remember to provide a reviewer password if the datasets are not yet public (see https://www.embopress.org/page/journal/17574684/authorguide#dataavailability).

In case you have no data that requires deposition in a public database, please state so in this section. Note that the Data Availability Section is restricted to new primary data that are part of this study.

6) For data quantification: please specify the name of the statistical test used to generate error bars and P values, the number (n) of independent experiments (specify technical or biological replicates) underlying each data point and the test used to calculate p-values in each figure legend. The figure legends should contain a basic description of n, P and the test applied. Graphs must include a description of the bars and the error bars (s.d., s.e.m.).

7) We would also encourage you to include the source data for figure panels that show essential data. Numerical data should be provided as individual .xls or .csv files (including a tab describing the data). For blots or microscopy, uncropped images should be submitted (using a zip archive if multiple images need to be supplied for one panel). Additional information on source data and instruction on how to label the files are available at
.

8) Our journal encourages inclusion of *data citations in the reference list* to directly cite datasets that were re-used and obtained from public databases. Data citations in the article text are distinct from normal bibliographical citations and should directly link to the database records from which the data can be accessed. In the main text, data citations are formatted as follows: "Data ref: Smith et al, 2001" or "Data ref: NCBI Sequence Read Archive PRJNA342805, 2017". In the Reference list, data citations must be labeled with "[DATASET]". A data reference must provide the database name, accession number/identifiers and a resolvable link to the landing page from which the data can be accessed at the end of the reference. Further instructions are available at .

9) We replaced Supplementary Information with Expanded View (EV) Figures and Tables that are collapsible/expandable online. A maximum of 5 EV Figures can be typeset. EV Figures should be cited as 'Figure EV1, Figure EV2" etc... in the text and their respective legends should be included in the main text after the legends of regular figures.
- For the figures that you do NOT wish to display as Expanded View figures, they should be bundled together with their legends in a single PDF file called *Appendix*, which should start with a short Table of Content. Appendix figures should be referred to in the main text as: "Appendix Figure S1, Appendix Figure S2" etc.
- Additional Tables/Datasets should be labeled and referred to as Table EV1, Dataset EV1, etc. Legends have to be provided in a separate tab in case of .xls files. Alternatively, the legend can be supplied as a separate text file (README) and zipped together with the Table/Dataset file.

See detailed instructions here:
.

10) The paper explained: EMBO Molecular Medicine articles are accompanied by a summary of the articles to emphasize the major findings in the paper and their medical implications for the non-specialist reader. Please provide a draft summary of your article highlighting
- the medical issue you are addressing,
- the results obtained and
- their clinical impact.

This may be edited to ensure that readers understand the significance and context of the research. Please refer to any of our published articles for an example.

11) For more information: There is space at the end of each article to list relevant web links for further consultation by our readers. Could you identify some relevant ones and provide such information as well? Some examples are patient associations, relevant databases, OMIM/proteins/genes links, author's websites, etc...

12) Every published paper now includes a 'Synopsis' to further enhance discoverability. Synopses are displayed on the journal webpage and are freely accessible to all readers. They include a short stand first (maximum of 300 characters, including space) as well as 2-5 one-sentences bullet points that summarizes the paper. Please write the bullet points to summarize the key NEW findings. They should be designed to be complementary to the abstract - i.e. not repeat the same text. We encourage inclusion of key acronyms and quantitative information (maximum of 30 words / bullet point). Please use the passive voice. Please attach these in a separate file or send them by email, we will incorporate them accordingly.

Please also suggest a striking image or visual abstract to illustrate your article as a PNG file 550 px wide x 300-600 px high.

EMBO Molecular Medicine has a "scooping protection" policy, whereby similar findings that are published by others during review or revision are not a criterion for rejection. Should you decide to submit a revised version, I do ask that you get in touch after three months if you have not completed it, to update us on the status.

I look forward to seeing a revised form of your manuscript as soon as possible.

With kind regards,

Lise Roth, Ph.D
Editor
EMBO Molecular Medicine

***** Reviewer's comments *****

Referee #1 (Comments on Novelty/Model System for Author):

The authors use several xenograft tumor models with specific genetic deficiencies. Although for some experiments it would be useful to examine tumor models with deficiencies for both BRCA1 and BRCA2 (instead of just one), the models are suitable and are utilized in well-designed experiments.

Referee #1 (Remarks for Author):

Groelly et al. (EMM-2021-14501) examine different aspects of the activity of a specific inhibitor, pyridostatin, a member of an interesting new class of anti-cancer agents that stabilize G4 quadruplexes. It has been published previously that BRCA1/2 deficient, and other HR deficient, cells are hypersensitive to pyridostatin and other stabilizers of G4 quadruplex DNA, such as CX-5461. These authors (Zimmer et al., 2016) and others (Xu et al. 2017), both of which are cited here, together have previously shown, similar to the current manuscript, that G4 stabilizers can kill BRCA1/2-deficient cells and tumors (in xenograft models), including those that are resistant to PARP inhibitors. In general, this study is well-designed, including the description of the results of xenograft experiments in Tables 1-5. More novel aspects of the current study, which may open new possibilities for the treatment of HR-deficient tumors, including those that are resistant to PARP inhibitors, include the findings that pyridostatin activates innate immune signaling via cGAS-STING, and initial tests of combinations of pyridostatin with paclitaxel and/or NU-7441 to more effectively inhibit the growth of BRCA1-deficient tumors. While many parts of the manuscript seem relatively incremental, these latter aspects are more novel. It is the recommendation of this reviewer, that along with appropriate revisions, this manuscript might be better suited to publication in EMBO Molecular Medicine as a short report that focuses on the activation of innate immune signaling and novel drug combinations.

Specific Comments:
1. While this may be the first demonstration of the sensitivity of BRCA1/2-deficient tumors to pyridostatin, this has previously demonstrated for different G4 ligands, RHPS4 and CX-5461 (Zimmer et al. 2016; Xu et al. 2017). Therefore, the finding of tumor sensitivity to G4 ligands is not by itself particularly novel. Those studies even use some of the same cell lines, such as DLD1 and HCC116 cells that are deficient for BRCA2.

2. Xu et al. (2017) show that BRCA1-deficient xenograft tumors developed from cells that are resistant to PARP inhibitor are sensitive to CX-5461. As such, the finding here that BRCA1-deficient/PARP inhibitor resistant tumors are hypersensitive to pyridostatin is not especially novel.

3. While the authors show that BRCA1/2-deficient tumors treated with pyridostation resume growth when treatment is stopped, and show that this involves c-NHEJ and can be suppressed by the DNA-PKcs inhibitor NU-7441, this is not unexpected since Xu et al. (2017) show that c-NHEJ factors are required for cellular resistance to CX-5461.

4. Many of the descriptions of the cellular DNA damage response to G4 stabilizers, such as increased DNA double-strand breaks and chromosome breakage, activation of ATM/ATR signaling, induction of gamma-H2AX foci, and effects on DNA replication, have been documented previously by Zimmer et al. (2016) and Xu et al. (2017) albeit often with agents other than pyridostatin or depletion of other homologous recombination factors such as RAD51.

5. In Fig. S3B, the authors demonstrate that PRKDC WT and KO HAP1 cells display the same viability over a range of concentrations of NU-7441. This is not convincing evidence that NU-7441 is a specific inhibitor of DNA-PKcs. A better experiment might be to compare the viability of PRKDC KO cells to vehicle vs NU-7441.

6. The authors utilize male mice for xenograft experiments in Fig. 1/Tables 1-2 and female mice in experiments shown in Fig. 3/Table 3. At a minimum, there should be a justification for this and perhaps also discussion of how this may affect the results that were obtained.

7. Innate immune signaling is tested in H1299 cells with DOX-inducible shBRCA2, but no indication is given as to why this system rather than BRCA2-deficient DLD1 and/or HCT116 cells were utilized as elsewhere. Additionally, it might strengthen the manuscript to show activation of innate immune signaling in more than one BRCA2-deficient cell line.

8. Since combination treatments (pyridostatin, paclitaxel and/or NU-7441) is one of the most novel parts of this study, the conclusions should be strengthened by testing in vitro sensitivity to the various combinations, in addition to the in vivo experiments that have been performed.

9. Combination treatments including pyridostatin, paclitaxel and/or NU-7441 are performed with BRCA1-deficient MDA-MB-436 cells. The conclusions would be strengthened by testing BRCA2-proficient vs deficient xenografts in parallel.

10. The authors should consider whether combinations can be developed in which at least one of the drugs can be continued for maintenance, since this might make the strategy particularly effective.

11. It might be useful for the authors to consider, in the Discussion, whether there is any reason the use of pyridostatin is

preferable to CX-5461 for future pre-clinical studies.

12. In the Materials and Methods, if the authors developed H1299 cell lines carrying an inducible shBRCA2, more information on this should be given. Alternatively, the commercial source should be given.

Referee #2 (Comments on Novelty/Model System for Author):

Novelty: A previous publication (Xu et al., Nat Commun 2016) reports that another G-quadruplex stabilizer CX-5461 currently in clinical trials is selectively lethal to BRCA1/2 deficient tumors, and also presents an extensive analysis of underlying mechanisms which overlaps with the results & conclusions in this paper. The novelty of the current manuscript is limited.

Medical impact and adequacy of model system: Several factors limit potential impact in molecular medicine. Many of the experiments are carried out using DLD-1 colorectal cancer cells, which is not ideal because these cells are deficient in mismatch repair & accumulate point mutations at a very high rate. The clinical use of PDS is not yet clear, but other G4-stabilizers are already in clinical trials. So, it would be better to repeat at least key experiments using alternative cells & drugs.

Referee #2 (Remarks for Author):

In their manuscript entitled "Anti-tumoral activity of the G-quadruplex ligand pyridostatin against BRCA1/2-deficient tumors", Groelly, Porru and colleagues take advantage of various mouse model systems to demonstrate the selective toxicity of the G-quadruplex stabilizer pyridostatin (PDS) towards BRCA1/BRCA2-deficient tumors, including PARP-inhibitor resistant xenografts. In addition, the authors evaluate various drug combinations that act in combination with PDS in targeting BRCA-deficient xenografts in mice. The manuscript is a logical extension of the authors' previous work (Zimmer et al., Mol Cell 2016), confirming and extending their previous in vitro findings.

Major concerns:
1. A previous publication (Xu et al., Nat Commun 2016) reports that another G-quadruplex stabilizer CX-5461 currently in clinical trials is selectively lethal to BRCA1/2 deficient tumors, and also presents an extensive analysis of underlying mechanisms which overlaps with the results & conclusions in this paper. The novelty of the current manuscript is limited.

2. The overlaps between this manuscript and the experiments reported by Xu et al (2016) need to be thoroughly discussed. The Introduction should clearly explain how the present study is novel compared to the previously reported one. Also, the Results section needs to fully discuss similarities and differences with results of Xu et al (2016).

3. Mechanistic conclusions seem preliminary.
(a) The authors say that PDS disrupts replication causing DSBs that can be repaired by NHEJ. However PDS also stabilizes G4 structures triggering a DNA-damage response at telomeres (Rodriguez et al J Am Chem Soc 2008), and the authors have previously found that PDS affects telomere replication in BRCA deficient cells (Zimmer et al., 2016). Origin of DNA damage induced by PDS is unclear, and further experiments are needed.
(b) The authors say that PDS activates cGAS/STING in BRCA-deficient cells. Previous reports show that BRCA deficiency itself strongly activates cGAS/STING pathway & downstream signaling. Fig 5B shows little or no activation of cGAS/STING-induced transcription by BRCA2 shRNA alone. This mechanism needs to be clarified.
(c) The authors imply in the abstract and text that paclitaxel potentiates in vivo toxicity of PDS via cGAS-dependent apoptosis. The results shown are not enough to support this mechanism, they only show that the effect of PDS on tumor growth is enhanced by paclitaxel (which has many mechanisms of action).

4. The results of the cell fractionation experiments in Figure 3 are somewhat surprising. Is the lack of XRCC4 expression in Brca1-/-Tp53bp1-/- cells expected? Is it supported by WB data in whole cell lysates? If that is the case, blots showing whole cell lysates would be more informative.

5. Considerably higher concentrations of PDS and longer drug treatments were used to induce innate immune signalling (Figure 5) than the conditions used for the other experiments in the manuscript. In fact, levels of PDS that induce substantial DNA damage do not seem to be sufficient to activate the cGAS/STING pathway. This could mean that the mechanisms for cGAS activation by PDS are different from those causing DNA damage.

6. Finally, several factors limit potential impact in molecular medicine. Many of the experiments are carried out using DLD-1 colorectal cancer cells, which is not ideal because these cells are deficient in mismatch repair & accumulate point mutations at a very high rate. The clinical use of PDS is not yet clear, but other G4-stabilizers are already in clinical trials. So, it would be better to repeat at least key experiments using alternative cells & drugs.
Minor comments
1. Scattered typos and grammatical mistakes (e.g., "consistent our previously ..." - page 6; "tumors were allowed grow" - page 36; etc.).
2. Some conclusions should be moderated (e.g., on page 7 the authors write: "pyridostatin ... effectively and specifically

eliminates ... tumors lacking BRCA2". This is not supported by the data in Fig 1, which shows a lack of tumor growth at best. Xu et al (2016) also show effects of Gq stabilizers on tumors with other DNA repair deficiencies, suggesting the effects are not totally selective.
3. Page 10, last paragraph should refer to Fig 2, not to Fig 1.
4. Page 18, end of first paragraph should read "...47% and 40% increase in survival ..."
5. The antibody list in the Materials and Methods section includes antibodies not used in the manuscript and lacks antibodies shown (e.g., POLQ).
6. Legend for Fig 2G: please delete "BRCA2-proficient (+BRCA2)".
7. Figure 5B: please indicate PDS concentrations.
8. Figure 6D: statistical significance is described in legend but not shown in the figure.
9. Table 4 consists of one line. In general, the Tables might be better suited for supplementary material.

Referee #3 (Remarks for Author):

Groelly and Porru et al. provide a comprehensive and systematic study demonstrating the viability of using G4 stabilizers, like pyridostatin (PDS), as an effective chemotherapeutic in an in vivo xenograft model. More importantly, the authors further extend these findings towards selectively targeting BRCA1-mutated xenograft tumors that have developed PARPi resistance, a prevalent and clinically significant outcome when using PARPi to target HR-deficient tumors. Additionally, they demonstrate that pyridostatin in combination with the DNA-PKcs inhibitor, Nu-7441 and microtubule-stabilizing agent, paclitaxel, have potent anti-tumor activity in BRCA- deficient tumors and propose the use of this combinational therapeutics in treatment of BRCA1/2 mutant tumors.
This manuscript provides an important body of work on the potential therapeutic use and effects of PDS, yet some of the conclusions presented in this study are somewhat premature and lack sufficient experimental support, especially with respect to the specific repair pathways utilized in the repair of G4 lesions and would benefit from further investigations. Nevertheless, these do not diminish the overall importance of the data and observations presented in the manuscript, which is of significant interest to the scientific and clinical community at large. I therefore recommend this paper for publication, and ask that the authors address several (minor) points detailed below, and further elaborate in their discussion on possible mechanisms:

1. In previous work by Olivieri et al Cell 2020, the genetic factors involved in DNA damage response were mapped for different therapeutic insults, and categorized according to specific pathways, where PDS was found to be strongly associated with end joining pathway. The Olivieri study and other recent studies (Bruno et al PNAS 2020 - PMCID: PMC7049172; Pipier et al Biorxiv 2020) have linked G-quadruplex ligands to activity of topoisomerase 2, and lesions that are repaired by the NHEJ pathway. On the other hand, drugs that induce replication fork stalling and collapse such as the Topoisomerase I poison CPT2 and PARP inhibitor Olaparib were strongly correlated with HR repair, which is anticipated to facilitate the repair of lesions that occur following fork collapse (rather than end joining). Overall, these studies indicate that lesions induced by PDS will rely on repair via NHEJ whereas lesions induced by PARP inhibition will utilize HR. In the present study, the authors relate PDS treatment to replication fork and use it to target BRCA and HR deficient models, a result that seems to differ from the specific repair pathways that were previously assigned. While I realize that the assignment of repair pathways in Olivieri et al is somewhat lacking with respect to the specific definition of repair mechanisms and the mechanistic complexity of said pathways, I would ask that the authors discuss and at least speculate on the various lesions formed and their repair mechanisms. A possible scenario that the authors might want to consider is that PDS can induce two (or more) types of lesions, one type of lesion in forks that is repaired by HR and another lesion that is repaired by NHEJ. This could be a plausible scenario, considering that PDS might act as a DNA structure driven topoisomerase 2 poison, and the fact that the top2 poison etoposide induces lesions that are either repaired by NHEJ by also HR as demonstrated by Olivieri et al, (in Fig S1B). Such discussion will be very beneficial to readers who might find these results confusing or conflicting, while also providing a conceptual framework for future mechanistic studies. In their discussion, the authors should also consider additional reports that have also used other G-quadruplex ligands to show effect on HR proficient cells (for example PMID: 33173151 and PMID: 32457376)
2. The authors provide an interesting finding that PDS in combination with the DNA-PKcs inhibitor and microtubule-stabilizing agent, paclitaxel, as a therapeutic strategy to target BRCA1- deficient tumors. However, exacerbation of DNA-damage via these methods could potentially have detrimental effects on tumors irrespective of their BRCA1/2 status. I would recommend the assessment of these effects by replicating the experiments in Figure 6C in a BRAC1/2 proficient xenograft tumor model before posing it as a therapeutic strategy specifically for patients with BRCA1/2 mutations. If this is not possible, I would ask that the authors would comments about this matter.
3. Figure 5B: the authors show the error bars, but not if the differences between the samples are significant. What are the associated p-values?
4. Figure 1D lack statistics and smaple size. The authors showed show error bars and reflect the significance and relevant difference between the PDS and Talazoparib.
5. The authors should provide representative images of IF staining for all samples, and particularly of gH2Ax foci (Fsuch as the data in Figure 2F)and 53BP1 foci. These can be included as new SI figures.

We, the authors, are grateful to the Referees for their constructive comments on our manuscript. We have addressed all the points raised by the Referees, which significantly improved our manuscript.

Point-by-point response:

Referee #1:

The authors use several xenograft tumor models with specific genetic deficiencies. Although for some experiments it would be useful to examine tumor models with deficiencies for both BRCA1 and BRCA2 (instead of just one), the models are suitable and are utilized in well-designed experiments.

Remarks for Author:
Groelly et al. (EMM-2021-14501) examine different aspects of the activity of a specific inhibitor, pyridostatin, a member of an interesting new class of anti-cancer agents that stabilize G4 quadruplexes. It has been published previously that BRCA1/2 deficient, and other HR deficient, cells are hypersensitive to pyridostatin and other stabilizers of G4 quadruplex DNA, such as CX-5461. These authors (Zimmer et al., 2016) and others (Xu et al. 2017), both of which are cited here, together have previously shown, similar to the current manuscript, that G4 stabilizers can kill BRCA1/2-deficient cells and tumors (in xenograft models), including those that are resistant to PARP inhibitors. In general, this study is well-designed, including the description of the results of xenograft experiments in Tables 1-5. More novel aspects of the current study, which may open new possibilities for the treatment of HR-deficient tumors, including those that are resistant to PARP inhibitors, include the findings that pyridostatin activates innate immune signaling via cGAS-STING, and initial tests of combinations of pyridostatin with paclitaxel and/or NU-7441 to more effectively inhibit the growth of BRCA1-deficient tumors. While many parts of the manuscript seem relatively incremental, these latter aspects are more novel. It is the recommendation of this reviewer, that along with appropriate revisions, this manuscript might be better suited to publication in EMBO Molecular Medicine as a short report that focuses on the activation of innate immune signaling and novel drug combinations.

Specific Comments:
1. While this may be the first demonstration of the sensitivity of BRCA1/2-deficient tumors to pyridostatin, this has previously demonstrated for different G4 ligands, RHPS4 and CX-5461 (Zimmer et al. 2016; Xu et al. 2017). Therefore, the finding of tumor sensitivity to G4 ligands is not by itself particularly novel. Those studies even use some of the same cell lines, such as DLD1 and HCC116 cells that are deficient for BRCA2.
Response: As the Referee points out, other G4 ligands (RHPS4 and CX-5461) have been shown to be active against BRCA1/2-deficient xenograft tumours established in mice. However, these have not yet been successfully used in cancer patients with *BRCA* mutations, which develop resistance to targeted therapies (e.g. PARP inhibitors; PARPi). Therefore, it is imperative to identify new G4 ligands that not only eliminate BRCA-deficient tumours, but also counteract resistant disease. Here, we demonstrate that pyridostatin inhibits growth of BRCA1/2-deficient xenograft tumours in mice, which recapitulates its activity *in vitro*. Importantly, pyridostatin has very low *in vivo* toxicity relative to CX-5461, the only G4-binding compound currently in clinical trials (see answer to point 11 below). This is

a key novel aspect of pyridostatin biology, essential for establishing its translational potential.

2. Xu et al. (2017) show that BRCA1-deficient xenograft tumors developed from cells that are resistant to PARP inhibitor are sensitive to CX-5461. As such, the finding here that BRCA1-deficient/PARP inhibitor resistant tumors are hypersensitive to pyridostatin is not especially novel.
Response: The Referee correctly points out that Xu et al. (2017) showed that CX-5461 is active against BRCA1-deficient xenograft tumours established from cells that are resistant to PARP inhibitors (PARPi). Here, we demonstrate that pyridostatin is not only active against tumours established from cells, but also against tumours obtained from patients (PDXs) that have acquired resistance to PARPi. As an additional novel aspect of our work, we demonstrate mechanistically that loss of classical non-homologous end joining (c-NHEJ) repair underlies the sensitivity of BRCA1-deficient PARPi-resistant tumours to pyridostatin.

3. While the authors show that BRCA1/2-deficient tumors treated with pyridostation resume growth when treatment is stopped, and show that this involves c-NHEJ and can be suppressed by the DNA-PKcs inhibitor NU-7441, this is not unexpected since Xu et al. (2017) show that c-NHEJ factors are required for cellular resistance to CX-5461.
Response: As the Referee indicates, Xu et al (2017) showed that abrogating c-NHEJ sensitises cells in culture to pyridostatin. However, this does not necessarily imply that abrogating c-NHEJ repair also sensitises BRCA1/2-deficient cells and tumours to pyridostatin, as other backup DNA repair pathways act in the context of BRCA-deficiency and may be involved in the repair of pyridostatin-inflicted damage in these cells. Here we demonstrate for the first time that chemical inhibition of c-NHEJ (using DNA-PK inhibitor NU-7441) inhibits growth of BRCA1/2-deficient cells and tumours and that NU-7441 acts synergistically with pyridostatin and paclitaxel in targeting these tumours.

4. Many of the descriptions of the cellular DNA damage response to G4 stabilizers, such as increased DNA double-strand breaks and chromosome breakage, activation of ATM/ATR signaling, induction of gamma-H2AX foci, and effects on DNA replication, have been documented previously by Zimmer et al. (2016) and Xu et al. (2017) albeit often with agents other than pyridostatin or depletion of other homologous recombination factors such as RAD51.
Response: Distinctly from previous studies, in our current work we use the DNA damage response (i.e. γH2AX foci, ATM/ATR activation) as means to study the recovery of BRCA1/2-deficient cells after removal of pyridostatin. This has now been clarified in Fig 2 and Fig 4, and corresponding figures legends. In other words, we investigate whether the DNA damage inflicted by pyridostatin can be repaired in cells lacking homologous recombination (i.e. BRCA1/2-deficient) and, if so, which repair pathways are required. This approach enabled us to define a key resistance mechanism to pyridostatin, uniquely mediated by c-NHEJ, and modalities to counteract it. Moreover, we demonstrate the DNA damage inflicted by pyridostatin in BRCA1/2-deficient cells, including those resistant to PARPi, triggers cGAS/STING dependent innate immune response. These results highlight the potential of pyridostatin treatment to facilitate tumour recognition by the immune system *in vivo.*

5. In Fig. S3B, the authors demonstrate that PRKDC WT and KO HAP1 cells display the

same viability over a range of concentrations of NU-7441. This is not convincing evidence that NU-7441 is a specific inhibitor of DNA-PKcs. A better experiment might be to compare the viability of PRKDC KO cells to vehicle vs NU-7441.
Response: Following the Referee's suggestion, in the new Figure S4B we represent the viability data in *PRKDC* KO cells as NU-7441 effect relative to DMSO. Importantly, the specificity of NU-7441 for DNA-PKcs is further demonstrated in Fig. S4D, where NU-7441 potentiates the cytotoxicity of pyridostatin in wild type cells, but not in *PRKDC* KO cells.

6. The authors utilize male mice for xenograft experiments in Fig. 1/Tables 1-2 and female mice in experiments shown in Fig. 3/Table 3. At a minimum, there should be a justification for this and perhaps also discussion of how this may affect the results that were obtained.
Response: We believe that the exclusive use of male animals would be less informative for future clinical trials and therefore included animals of both sexes in our *in vivo* experiments. This should strengthen the possible clinical use of pyridostatin for the treatment of *BRCA1/2*-mutated tumours.

7. Innate immune signaling is tested in H1299 cells with DOX-inducible shBRCA2, but no indication is given as to why this system rather than BRCA2-deficient DLD1 and/or HCT116 cells were utilized as elsewhere. Additionally, it might strengthen the manuscript to show activation of innate immune signaling in more than one BRCA2-deficient cell line.
Response: We would like to thank the Referee for this suggestion. Consistently, we have included in the new manuscript (Fig 3D) data showing that pyridostatin induces innate immune responses in BRCA2-deleted RPE-1 cells, which substantially strengthens our findings.

8. Since combination treatments (pyridostatin, paclitaxel and/or NU-7441) is one of the most novel parts of this study, the conclusions should be strengthened by testing in vitro sensitivity to the various combinations, in addition to the in vivo experiments that have been performed.
Response: In response to the Referee's suggestion, we have included in new Fig S6 *in vitro* data from clonogenic survival assays showing the response of BRCA1-deficient MDA-MB-436 cells to treatment with pyridostatin, paclitaxel and NU-7441, as well as their various combinations.

9. Combination treatments including pyridostatin, paclitaxel and/or NU-7441 are performed with BRCA1-deficient MDA-MB-436 cells. The conclusions would be strengthened by testing BRCA2-proficient vs deficient xenografts in parallel.
Response: To address this point (also raised by Referee #3, point 2) we have included in new Fig S7 and new Fig S8 *in vivo* data showing the response of xenografts established using HCT116 *BRCA2*$^{+/+}$ and *BRCA2*$^{-/-}$ cells, to the double and triple combinations of pyridostatin, paclitaxel and NU-7441. These new data demonstrate that the pyridostatin/paclitaxel/NU-7441 combination selectively targets the *BRCA2*$^{-/-}$ xenograft tumours and leads to increased survival in mice.

10. The authors should consider whether combinations can be developed in which at least one of the drugs can be continued for maintenance, since this might make the strategy particularly effective.
Response: On pages 20-21 of the revised manuscript we discuss whether and how any of the drugs used in the triple combination could be used in maintenance therapy.

11. It might be useful for the authors to consider, in the Discussion, whether there is any reason the use of pyridostatin is preferable to CX-5461 for future pre-clinical studies.

Response: We observed that pyridostatin used in mice at the maximum tolerated dose had a higher activity against BRCA1/2-deficient tumours than CX-5461 (at a dose used by Xu et al., 2017). We included these results in the Table for Referees below. In Discussion, we mention that CX-5461, which also acts as an inhibitor of rDNA transcription by preventing RNA polymerase I binding to rDNA promoters (Drygin et al., Cancer Res. 2011), is likely to have adverse effects in patients, as indicated by a recent clinical dose-escalation study (Khot et al., Cancer Disc. 2019).

Table for Referees. *In vivo* anti-tumour efficacy of pyridostatin and CX-5461 on *BRCA2*$^{-/-}$ DLD1 and *BRCA2*$^{-/-}$ HCT116 xenografts

CB17-SCID male mice were were injected intramuscularly with $5x10^6$ cells per mouse. Tumours were allowed grow to approximately 250 mm^3 before initiation of treatment (day 1). Mice were treated with with pyridostatin (*i.v.*; 7.5 mg/kg/day) for five consecutive days, followed by two-day break and five more days of treatment or with CX-5461(*o.s.*; 50 mg/kg/day) three times, once every three days. Each experimental group included n = 5 mice. Tumour weight inhibition was calculated at the nadir of the effect using the formula: (1 - [tumour weight in treated mice] / [tumour weight in untreated mice]) x100 and expressed as average for n = 5 mice in each group. Tumour growth delay was calculated as the median time in days required for untreated and treated tumours to reach the same size. Stable disease was defined as mice in which tumour volume did not change for at least two weeks after initiation of treatment. Body weight loss is reported as weight at the end of treatment relative to the first day of treatment (%), as average for n = 5 mice in each group.

Treatment	Tumour weight inhibition (%)	Tumour growth delay (days)	Stable disease	Body weight loss (%)	Toxic deaths
***BRCA2*$^{-/-}$ DLD1 Pyridostatin**	66	10	2/5	0	0/5
***BRCA2*$^{-/-}$ DLD1 CX-5461**	46	6	0/5	25	2/5
***BRCA2*$^{-/-}$ HCT116 Pyridostatin**	59	9	1/5	0	0/5
***BRCA2*$^{-/-}$ HCT116 CX-5461**	47	14	0/5	24	1/5

12. In the Materials and Methods, if the authors developed H1299 cell lines carrying an inducible shBRCA2, more information on this should be given. Alternatively, the commercial source should be given.

Response: The H1299 cells containing the DOX-inducible shRNA against BRCA2 have been previously reported: Lai et al., *Nature Communications* 8, 2017; Reislander et al., *Nature Communications* 10, 2019. These references have been included in Materials and Methods.

Referee #2
Novelty: A previous publication (Xu et al., Nat Commun 2016) reports that another G-quadruplex stabilizer CX-5461 currently in clinical trials is selectively lethal to BRCA1/2 deficient tumors, and also presents an extensive analysis of underlying mechanisms which overlaps with the results & conclusions in this paper. The novelty of the current manuscript is limited.

Medical impact and adequacy of model system: Several factors limit potential impact in molecular medicine. Many of the experiments are carried out using DLD-1 colorectal cancer cells, which is not ideal because these cells are deficient in mismatch repair & accumulate point mutations at a very high rate. The clinical use of PDS is not yet clear, but other G4-stabilizers are already in clinical trials. So, it would be better to repeat at least key experiments using alternative cells & drugs.

Remarks for Author:
In their manuscript entitled "Anti-tumoral activity of the G-quadruplex ligand pyridostatin against BRCA1/2-deficient tumors", Groelly, Porru and colleagues take advantage of various mouse model systems to demonstrate the selective toxicity of the G-quadruplex stabilizer pyridostatin (PDS) towards BRCA1/BRCA2-deficient tumors, including PARP-inhibitor resistant xenografts. In addition, the authors evaluate various drug combinations that act in combination with PDS in targeting BRCA-deficient xenografts in mice. The manuscript is a logical extension of the authors' previous work (Zimmer et al., Mol Cell 2016), confirming and extending their previous in vitro findings.

Major concerns:
1. A previous publication (Xu et al., Nat Commun 2016) reports that another G-quadruplex stabilizer CX-5461 currently in clinical trials is selectively lethal to BRCA1/2 deficient tumors, and also presents an extensive analysis of underlying mechanisms which overlaps with the results & conclusions in this paper. The novelty of the current manuscript is limited.
Response: As the Referee correctly points out, some G4 ligands have entered clinical trials, with CX-5461 as a prominent example. Key to their success is good tolerability in patients and effective tumour targeting. Here we propose that pyridostatin is a promising candidate for future clinical development, because it shows low animal toxicity under our experimental conditions (see response to Referee #1, point 11) and efficient targeting of BRCA1/2-deficient tumours in mice. In addition to demonstrating the clinical potential for pyridostatin, the work presented in our manuscript is novel and furthers our understanding of G4 ligand biology for the following reasons: 1. Mechanistically, we show that PARPi resistant BRCA1-deficient cells lack of c-NHEJ, which is critical for their susceptibility to pyridostatin; 2. We furthermore demonstrate that c-NHEJ provides the main pathway for repair of pyridostatin-induced DNA damage in the absence of BRCA1 or BRCA2, and therefore represents a key target for counteracting pyridostatin resistance; 3. Consistent with the former, we demonstrate synergy between pyridostatin and the DNA-PKcs inhibitor NU-7441 against BRCA1/2-deficient tumours; the specific toxicity is further enhanced by the triple combination with paclitaxel; 4. We show that pyridostatin triggers cGAS/STING-dependent immune responses in BRCA1/2-deficient cells, including those that have acquired PARPi resistance, and that this correlates with the pyridostatin ability to inflict ATM-activating DNA damage.

2. The overlaps between this manuscript and the experiments reported by Xu et al (2016) need to be thoroughly discussed. The Introduction should clearly explain how the present study is novel compared to the previously reported one. Also, the Results section needs to fully discuss similarities and differences with results of Xu et al (2016).
Response: As requested by the Referee, we highlighted in the Discussion (page 17-18) the differences between our current work and that of Xu et al. (2017) and Zimmer et al. (2016), which reported sensitisation to G4 ligands of cells lacking HR or NHEJ. We emphasized how our findings may help elucidate the mechanism of action of G4 ligands and their combinations with other drugs, thereby increasing their potential for future clinical development.

3. Mechanistic conclusions seem preliminary.

(a) The authors say that PDS disrupts replication causing DSBs that can be repaired by NHEJ. However PDS also stabilizes G4 structures triggering a DNA-damage response at telomeres (Rodriguez et al J Am Chem Soc 2008), and the authors have previously found that PDS affects telomere replication in BRCA deficient cells (Zimmer et al., 2016). Origin of DNA damage induced by PDS is unclear, and further experiments are needed.
Response: In the revised text (page 17) we explain that the replication defects and DSBs induced by pyridostatin are inflicted throughout the genome, including the telomeres. We propose that regardless of their position in the genome, DSBs activate the same signalling pathways and are repaired by the same mechanisms.

(b) The authors say that PDS activates cGAS/STING in BRCA-deficient cells. Previous reports show that BRCA deficiency itself strongly activates cGAS/STING pathway & downstream signaling. Fig 5B shows little or no activation of cGAS/STING-induced transcription by BRCA2 shRNA alone. This mechanism needs to be clarified.
Response: As the Referee points out, our previous work demonstrated activation of the cGAS/STING pathway and downstream innate immune signalling upon BRCA2 abrogation. However, these former experiments were performed under chronic BRCA2 depletion (28 days of DOX treatment; clarified on page 10), in contrast with Fig 3C (formerly Fig 5B) where we used short-term BRCA2 abrogation (3 days of DOX treatment). Under these conditions, and in the absence of genotoxic treatments, innate immune responses are not activated in BRCA2-deficient cells. However, pyridostatin triggers robust cGAS/STING activation in these cells, caused by the rapid increase in DNA damage levels.

(c) The authors imply in the abstract and text that paclitaxel potentiates in vivo toxicity of PDS via cGAS-dependent apoptosis. The results shown are not enough to support this mechanism, they only show that the effect of PDS on tumor growth is enhanced by paclitaxel (which has many mechanisms of action).
Response: We agree with the Referee that our data do not show an effect of paclitaxel on cGAS-dependent responses and this is now reflected in the Abstract and text of the revised manuscript.

4. The results of the cell fractionation experiments in Figure 3 are somewhat surprising. Is the lack of XRCC4 expression in Brca1-/-Tp53bp1-/- cells expected? Is it supported by WB data in whole cell lysates? If that is the case, blots showing whole cell lysates would be more informative.

Response: In response to the Referee's request, we included in the new Fig S6A Western blots showing the XRCC4 levels in whole cell extracts prepared from the same cell lines.

5. Considerably higher concentrations of PDS and longer drug treatments were used to induce innate immune signalling (Figure 5) than the conditions used for the other experiments in the manuscript. In fact, levels of PDS that induce substantial DNA damage do not seem to be sufficient to activate the cGAS/STING pathway. This could mean that the mechanisms for cGAS activation by PDS are different from those causing DNA damage.
Response: The Referee questions whether the innate immune signalling triggered by pyridostatin in BRCA2-deficient cells is due to DNA damage accumulation, or to some other, non-specific effect of the treatment. To address this concern, in addition to the results showing dose-dependent induction of DNA damage and immune responses by pyridostatin in BRCA2-deficient cells (Fig 3A) we performed the following experiments: 1. Time course experiments to monitor the kinetics of DNA damage induction and cGAS/STING pathway activation in BRCA2-deficient H1299 cells treated with 10 μM pyridostatin. The results shown in new Fig 3B demonstrate similar increase in pKAP1 (a marker for ATM-dependent DNA damage response) and in pIRF3 (a marker for cGAS/STING-dependent immune response) with increased exposure to pyridostatin; 2. Quantification of cGAS-labelled micronuclei under the same experimental conditions (new Fig 3E,F), shows time-dependent induction. Taken together, these new results demonstrate that the cGAS/STING responses are activated in cells lacking BRCA2 by pyridostatin-inflicted DNA damage.

6. Finally, several factors limit potential impact in molecular medicine. Many of the experiments are carried out using DLD-1 colorectal cancer cells, which is not ideal because these cells are deficient in mismatch repair & accumulate point mutations at a very high rate. The clinical use of PDS is not yet clear, but other G4-stabilizers are already in clinical trials. So, it would be better to repeat at least key experiments using alternative cells & drugs.
Response: The experiments showing that pyridostatin inflicts DNA damage were carried out in H1299 (non-small-cell lung carcinoma) and in RPE1 (retinal pigment epithelial) human cells, in addition to DLD1 cells. Moreover, the *in vivo* potential of pyridostatin was evaluated not only in *BRCA2*$^{+/+}$ and *BRCA2*$^{-/-}$ DLD1-derived xenografts, but also in MDA-MB-436-derived, BRCA1-deficient xenografts (Fig 6), in patient derived xenografts from patients with WT or germline *BRCA1* mutation (Fig 5), as well as in *BRCA2*$^{+/+}$ and *BRCA2*$^{-/-}$ HCT116-derived xenografts (Figs S7, S8; requested by Referee #1, point 9 and Referee #3, point 2), the same model used in Xu et al. (2016). CX-5461 was not used due to its enhanced toxicity in our experimental conditions (see point 2 above and Referee #1, point 11).

Minor comments:

1. Scattered typos and grammatical mistakes (e.g., "consistent our previously ..." - page 6; "tumors were allowed grow" - page 36; etc.).
Response: We have corrected these errors in the revised manuscript and are grateful to the Referee for pointing them out.

2. Some conclusions should be moderated (e.g., on page 7 the authors write: "pyridostatin ... effectively and specifically eliminates ... tumors lacking BRCA2". This is not supported by the data in Fig 1, which shows a lack of tumor growth at best. Xu et al (2016) also show effects of Gq stabilizers on tumors with other DNA repair deficiencies, suggesting the effects are not totally selective.

Response: We have altered the text on page 7 to indicate that pyridostatin specifically inhibits the growth of tumours lacking BRCA2.

3. Page 10, last paragraph should refer to Fig 2, not to Fig 1.
Response: We have corrected this in the revised text (page 12).

4. Page 18, end of first paragraph should read "...47% and 40% increase in survival ..."
Response: We have inserted "increase in survival" in the revised text.

5. The antibody list in the Materials and Methods section includes antibodies not used in the manuscript and lacks antibodies shown (e.g., POLQ).
Response: The revised manuscript contains a complete list of antibodies used in our experiments.

6. Legend for Fig 2G: please delete "BRCA2-proficient (+BRCA2)".
Response: We have deleted "BRCA2-proficient (+BRCA2)" in the Fig 2G legend. Indeed, as the Referee pointed out, only BRCA2-deficient DLD1 cells are shown in this figure.

7. Figure 5B: please indicate PDS concentrations.
Response: In the revised manuscript we have included PDS concentrations both in the figure and figure legend (Fig 3C).

8. Figure 6D: statistical significance is described in legend but not shown in the figure.
Response: We have now included a comprehensive statistical analysis of the survival of the different treatment groups in Table S1.

9. Table 4 consists of one line. In general, the Tables might be better suited for supplementary material.
Response: To ensure consistency with the other figures/tables showing tumour data, we think it best to keep Table 4 in the main text.

Referee #3
Remarks for Author:
Groelly and Porru et al. provide a comprehensive and systematic study demonstrating the viability of using G4 stabilizers, like pyridostatin (PDS), as an effective chemotherapeutic in an in vivo xenograft model. More importantly, the authors further extend these findings towards selectively targeting BRCA1-mutated xenograft tumors that have developed PARPi resistance, a prevalent and clinically significant outcome when using PARPi to target HR-deficient tumors. Additionally, they demonstrate that pyridostatin in combination with the DNA-PKcs inhibitor, Nu-7441 and microtubule-stabilizing agent, paclitaxel, have potent anti-tumor activity in BRCA- deficient tumors and propose the use of this combinational therapeutics in treatment of BRCA1/2 mutant tumors.

This manuscript provides an important body of work on the potential therapeutic use and effects of PDS, yet some of the conclusions presented in this study are somewhat premature and lack sufficient experimental support, especially with respect to the specific repair pathways utilized in the repair of G4 lesions and would benefit from further investigations. Nevertheless, these do not diminish the overall importance of the data and observations

presented in the manuscript, which is of significant interest to the scientific and clinical community at large. I therefore recommend this paper for publication, and ask that the authors address several (minor) points detailed below, and further elaborate in their discussion on possible mechanisms:

1. In previous work by Olivieri et al Cell 2020, the genetic factors involved in DNA damage response were mapped for different therapeutic insults, and categorized according to specific pathways, where PDS was found to be strongly associated with end joining pathway. The Olivieri study and other recent studies (Bruno et al PNAS 2020 - PMCID: PMC7049172; Pipier et al Biorxiv 2020) have linked G-quadruplex ligands to activity of topoisomerase 2, and lesions that are repaired by the NHEJ pathway. On the other hand, drugs that induce replication fork stalling and collapse such as the Topoisomerase I poison CPT2 and PARP inhibitor Olaparib were strongly correlated with HR repair, which is anticipated to facilitate the repair of lesions that occur following fork collapse (rather than end joining). Overall, these studies indicate that lesions induced by PDS will rely on repair via NHEJ whereas lesions induced by PARP inhibition will utilize HR. In the present study, the authors relate PDS treatment to replication fork and use it to target BRCA and HR deficient models, a result that seems to differ from the specific repair pathways that were previously assigned. While I realize that the assignment of repair pathways in Olivieri et al is somewhat lacking with respect to the specific definition of repair mechanisms and the mechanistic complexity of said pathways, I would ask that the authors discuss and at least speculate on the various lesions formed and their repair mechanisms. A possible scenario that the authors might want to consider is that PDS can induce two (or more) types of lesions, one type of lesion in forks that is repaired by HR and another lesion that is repaired by NHEJ. This could be a plausible scenario, considering that PDS might act as a DNA structure driven topoisomerase 2 poison, and the fact that the top2 poison etoposide induces lesions that are either repaired by NHEJ by also HR as demonstrated by Olivieri et al, (in Fig S1B). Such discussion will be very beneficial to readers who might find these results confusing or conflicting, while also providing a conceptual framework for future mechanistic studies. In their discussion, the authors should also consider additional reports that have also used other G-quadruplex ligands to show effect on HR proficient cells (for example PMID: 33173151 and PMID: 32457376)
Response: We thank the Referee for suggesting a discussion of the results reported by Olivieri et al. (2020), as well as by other studies, which propose that pyridostatin may also act as a topoisomerase 2 (Top2) inhibitor. Such dual mode of action is not uncommon among G4-binding compounds, as CX-5461 is also known to act as an inhibitor of RNA polymerase I (Drygin et al., Cancer Res. 2011). We are also grateful to the Referee for the excellent suggestion that pyridostatin might induce different types of DNA damage: some derived from G4 stabilisation and others from Topo2 trapping on DNA. Both types of lesions are caused by replication fork stalling at these sites and could be repaired by homologous recombination (HR) or NHEJ. As such, both types of damage should be deleterious to cells lacking BRCA1 or BRCA2. We have included a discussion of this possible scenario on page 18 of our revised manuscript.

2. The authors provide an interesting finding that PDS in combination with the DNA-PKcs inhibitor and microtubule-stabilizing agent, paclitaxel, as a therapeutic strategy to target BRCA1- deficient tumors. However, exacerbation of DNA-damage via these methods could potentially have detrimental effects on tumors irrespective of their BRCA1/2 status. I would recommend the assessment of these effects by replicating the experiments in Figure 6C in a BRAC1/2 proficient xenograft tumor model before posing it as a therapeutic strategy

specifically for patients with BRCA1/2 mutations. If this is not possible, I would ask that the authors would comments about this matter.

Response: To address this point (also raised by Referee #1, point 9) we have included in the new Fig S7 and Fig S8 *in vivo* data showing the response of HCT116 *BRCA2*$^{+/+}$ and *BRCA2*$^{-/-}$ xenografts to the double and triple combinations of pyridostatin, paclitaxel and NU-7441. These new data demonstrate specific toxicity of the pyridostatin/paclitaxel/NU-7441 combinations against HCT116 *BRCA2*$^{-/-}$ xenograft tumours, leading to increased survival in mice.

3. Figure 5B: the authors show the error bars, but not if the differences between the samples are significant. What are the associated p-values?

Response: We have included *p*-values in the new Fig 3C (previously Fig 5B) showing qRT-PCR data.

4. Figure 1D lack statistics and sample size. The authors should show error bars and reflect the significance and relevant difference between the PDS and Talazoparib.

Response: We have repeated the immunohistochemistry (IHC) staining for gH2AX using HCT116 xenograft tumours treated with pyridostatin and included error bars as well as statistical analyses (Fig 1E,F). The analyses done in DLD1 tumours, formerly in Fig 1D, have been moved to Fig S1.

5. The authors should provide representative images of IF staining for all samples, and particularly of gH2Ax foci (such as the data in Figure 2F) and 53BP1 foci. These can be included as new SI figures.

Response: As the Referee requested, we included representative images for γH2AX and 53BP1 foci in the new Fig. S2B.

29th Oct 2021

Dear Madalena,

Thank you for the submission of your revised manuscript to EMBO Molecular Medicine. I am extremely sorry for this unusual delay, which is due to the fact that we were waiting for the report from ref #2. Unfortunately, despite several chasers, this referee still has not delivered a report, and in order not to delay the process further, we decided to make a decision at this stage.
We have received the report from referee #1, who also commented on your responses to referee #3's concerns. Should referee #2 provide a report shortly, we will send it to you, with the understanding that we would not ask you for further-reaching revisions in addition to the ones required in the enclosed report.

As you will see below, while referee #1 acknowledges the effort that was made to respond to the referee's concerns, he/she also regrets that the novelty of this manuscript compared to previous work has not been sufficiently clarified.

Therefore, we would like you to revise the manuscript further along the lines suggested by this referee. In particular, we would suggest turning the manuscript into a report, focusing on the original advances presented in this manuscript by comparison with Zimmer et al 2016 and Xu et al 2017, and on the impactful new findings.

As EMBO Press usually encourages one single round of revisions, please be aware that this will be the last chance for you to address these points.

Additionally, please also address the following editorial issues:
- Please carefully check the references to your figures in the main text (current Appendix Fig. S6A and S7D are missing), and correct the appendix callouts to "Appendix Table Sx" or "Appendix Fig. Sx")
- Please note that the reference list should come before the figure legends, and each reference should list 10 authors max. before et al.
- As part of the EMBO Publications transparent editorial process initiative (see our Editorial at http://embomolmed.embopress.org/content/2/9/329), EMBO Molecular Medicine will publish online a Review Process File (RPF) to accompany accepted manuscripts.
This file will be published in conjunction with your paper and will include the anonymous referee reports, your point-by-point response and all pertinent correspondence relating to the manuscript. Let us know whether you agree with the publication of the RPF and as here, if you want to remove or not any figures from it prior to publication.
Please note that the Authors checklist will be published at the end of the RPF.

I look forward to receiving your revised manuscript.

With my best wishes,

Lise

Lise Roth, PhD
Editor
EMBO Molecular Medicine

***** Reviewer's comments *****

Referee #1 (Comments on Novelty/Model System for Author):

Novelty is rated as medium because, although there are clearly novel aspects, there is too much focus, in comparison, on relatively incremental findings. For example, more incremental findings are in main Figures 1-2, while more novel/impactful findings are in supplemental Figures 6-8. The medical impact is also rated as medium because there is not enough focus on the more novel and potentially impactful findings here. The authors often validate their in vitro findings using multiple cell lines with a deficiency for BRCA2 and utilize several xenograft tumor models with specific genetic deficiencies, so the model systems are adequate.

Also, I was asked to comment on whether the authors suitably addressed concerns raised by Reviewer 3 - and I would say yes. Information was added to the Discussion, as requested, to address point 1. The authors added new Fig. S7 and S8 to address

whether BRCA2-deficient xenografts respond to combinations that include pyridostatin. Further, the authors added statistics to most figures in response to points 3-4 (but are still missing from Figure S1B), and added representative images of IF staining to new Fig. S2B to address point 5.

Referee #1 (Remarks for Author):

This revised manuscript by Groelly et al. (EMM-2021-14501-V2) clearly made an effort to respond to my comments from the original review of this manuscript. For example, in response to point 5, the authors added a demonstration that NU-7441 is a specific inhibitor of DNA-PKcs using PRKDC KO cells (new Figure S4B), as requested, and also demonstrate that PRKDC is necessary for NU-7441 to potentiate cell killing by pyridostatin (new Figure S4D). Further, in response to point 7, they added a demonstration that pyridostatin induces innate immune responses in a second BRCA2 deficient cell line (new Figure 3D), as suggested. Similarly, the authors added new Figure S6 as in vitro experiments that parallel their in vivo demonstration that combinations with pyridostatin are effective. While the authors previously included xenograft experiments treating BRCA1-proficient vs deficient xenografts with drug combinations, in response to Rev. 1's point 9 and Rev. 3's point 2, they have added parallel experiments utilizing BRCA2-proficient vs deficient xenografts. Besides these new experiments, which are generally convincing, and in the opinion of this reviewer, greatly strengthen the manuscript, the authors have made additions to the Discussion, and to the Materials and Methods, that address my points 9-12. As for points 1-4, which were related to where the current manuscript overlaps with or is distinct from Zimmer et al. 2016 and Xu et al. 2017, the authors provide a reasonable response in the rebuttal, on a point-by-point basis, and/or by adding information to the manuscript (mostly in the Discussion). However, as currently written, the novelty of this manuscript is still largely unclear. In agreement with Reviewer 2 (point 2), it is my opinion that this could have been remedied by explaining in the Introduction and/or Results section, in some detail, how this manuscript is similar and different from Zimmer et al. 2016 and Xu et al. 2017, and by including a rationale there for why the authors focus on pyridostatin instead of CX-5461. There are novel and interesting findings in this manuscript, including effects of pyridostatin on DNA replication, induction of innate immune signaling and the effects of combination treatments including pyridostatin, but the authors should use Zimmer et al. and Xu et al. to set the stage up-front for these advances. At least as currently presented, this manuscript still feels incremental. Another way to emphasize the novelty of this manuscript might have been to move some or all of Figures 1-3, which appear somewhat incremental, to supplementary information and move some or all of Figures S6-8 on combinatorial treatments to the main figures. As a minor point, why does phospho-KAP1 increase after release from pyridostatin in Fig. 2E? Also, the first paragraph of p. 13 discusses Appendix Fig S4E, but there is no part E in Figure S4. Further, what is the importance of the finding that deficiency for 53BP1 results in an absence of detectable XRCC4 in the chromatin when a defect in C-NHEJ is already suggested by the deficiency for 53BP1?

Dear Lise,

thank you very much for the opportunity to revise our manuscript entitled “Anti-tumoral activity of the G-quadruplex ligand pyridostatin against BRCA1/2-deficient tumours” by Groelly, Porru *et al.* according to the comments of Referee #1.

As outlined in my email and suggested by the Referee, the revised version highlights more clearly in the Introduction and Results sections the clinical benefits of pyridostatin as a second drug with a mechanism of action similar to CX-5461, but superior in terms of toxicity. We also moved Figures 1 and 2 of the previous manuscript to the Appendix and, conversely, moved to the main text previous Appendix Figure S8, detailing the *in vivo* efficacy of pyridostatin and its combinations in a second tumour model (HCT116 cell-derived). Additionally, former Figure 4E showing absence of XRCC4 from chromatin fraction of PARPi-resistant cells (on which the Referee commented), was moved to Appendix Fig S8 containing immunoblots of the whole cell extracts from the same experiment. The latest text changes are highlighted in blue.

In addition, we addressed all Editorial requests, i.e. placing References before Figure Legends, corrected reference format and adding the missing references in the text for Appendix Figures S6A and S7D. We agree with RPF online publication.

We hope that with these revisions our manuscript meets the standards for publication in *EMBO Molecular Medicine.* Many thanks in advance for your consideration and we are looking forward to hearing from you.

With my best wishes,

Madalena Tarsounas, PhD
Professor of Molecular and Cell Biology
University of Oxford, Department of Oncology
CRUK/MRC Oxford Institute for
Radiation Biology, Oxford, UK
madalena.tarsounas@oncology.ox.ac.uk

30th Nov 2021

Dear Madalena,

Thank you for the submission of your revised manuscript to EMBO Molecular Medicine. We have now received the enclosed report from referee #1. As you will see, this referee is still not fully satisfied with the way the data are presented. We have discussed this issue within the team, and as the conclusions are well supported by the data and the technical quality of the manuscript is high (as mentioned by the referees), we think that there is no need to substantially change the manuscript at this point. Therefore, I am pleased to inform you that we will be able to accept your manuscript, once the following minor points will be addressed:

1/ Comments from Referee #1: Please address the issues on manuscript presentation. As mentioned above, we do not ask for a substantial rewriting at this point, but rather for minor modifications that would help the reader understand the medical impact and novelty of this manuscript compared to your previous work.

2/ Main manuscript text
- Please remove the blue in the text.
- Material and methods:
o Cells: please indicate whether the cells were authenticated (when applicable) and tested for mycoplasma contamination.
o Please make sure that the age and gender of the mice is indicated for all experiments.
o As PDX were generated, please add a paragraph on human samples, informed consent, and if applicable, conformity to the WMA Declaration of Helsinki and the Department of Health and Human Services Belmont Report. Please update the checklist accordingly.

3/ Figures:
- For all figures, please indicate in the figures or in their legends (including in the Appendix) the exact p= values, not a range (including for non-significant, ns). Some people found that to keep the figures clear, providing a supplemental table with all exact p-values was preferable. You are welcome to do this if you want to. Please also define in the figure legends (including in the appendix) the statistical test, as well as the bars and error bars, e.g. mean {plus minus} SD. Please define the number and the nature, i.e. biological or technical, of the replicates.
- Appendix Fig. S8 contains error bars based on n=2. Please use scatter blots showing the individual datapoints in these cases. The use of statistical tests needs to be justified.
- Please make sure that all figure panels with microscopy pictures contain scale bars defined in the legends (i.e. Appendix Fig. S2).

4/ Thank you for providing Source Data. Please double check the labeling of source data figure 2E (KAP1/S824KAP1).

5/ Thank you for providing The Paper Explained section. I shortened it a bit to fit our format, please let me know if you agree with the following:
Problem
Mutations in BRCA1 and BRCA2 are frequently found in familial cancers, including breast, ovarian and prostate cancers, as well as in sporadic cancers. Exploiting the vulnerabilities of BRCA1/2-mutated tumours with targeted therapies permits to specifically eliminate these tumours. However, resistance to standard chemotherapeutic regimens and to targeted therapies rapidly develops in patients. Therefore, there is an imperative need to identify novel drug candidates or treatment strategies to treat BRCA1/2-deficient tumours.
Results
We report that the G-quadruplex ligand pyridostatin specifically inflicts DNA damage and eliminates BRCA1/2-deficient tumours in vivo, including PDXs resistant to PARP inhibitors. We demonstrate that, in the absence of BRCA1/2, pyridostatin causes replication fork stalling and DNA double-strand breaks, which can be repaired by C-NHEJ reactions. Furthermore, we show that pyridostatin-inflicted DNA damage leads to formation of cGAS-associated micronuclei, which trigger innate immune responses. Finally, our study demonstrates that pyridostatin is well-tolerated in vivo and that its combination with paclitaxel and the NU-7441 DNA-PKcs inhibitor exhibits long-term anti-cancer activity against BRCA1/2-deficient tumours and substantially increases overall survival in mice.
Impact
Pyridostatin is a strong candidate drug for targeting BRCA1/2-deficient tumours and for overcoming PARPi resistance in vivo. The combination of this compound with DNA-PKcs inhibitors and paclitaxel could represent an effective treatment for the eradication of BRCA1/2-mutated tumours. Additionally, our results suggest that pyridostatin may potentiate the efficacy of immune checkpoint inhibitors. Thus, pyridostatin has a clear potential for further clinical development.

6/ Thank you for providing a synopsis text and figure. Please make sure that the image has the following dimensions: 550 px

wide x 300-600 px high, and that the text remains legible.

7/ As part of the EMBO Publications transparent editorial process initiative (see our Editorial at http://embomolmed.embopress.org/content/2/9/329), EMBO Molecular Medicine will publish online a Review Process File (RPF) to accompany accepted manuscripts.
This file will be published in conjunction with your paper and will include the anonymous referee reports, your point-by-point response and all pertinent correspondence relating to the manuscript. Let us know if you don't agree with the publication of the RPF and as here, or if you want to remove any figures from it prior to publication.

I look forward to receiving your revised manuscript.

With my best wishes,

Lise

Lise Roth, PhD
Editor
EMBO Molecular Medicine

***** Reviewer's comments *****

Referee #1 (Comments on Novelty/Model System for Author):

Technical quality is generally high with experiments being conducted in reinforcing cell systems, solid mouse experiments, and appropriate statistical analyses in most instances. While the portions on activation of innate immune signaling by pyridostatin and combination treatments are of interest, issues with the presentation still limit the apparent novelty and medical impact.

Referee #1 (Remarks for Author):

In response to previous comments and to better clarify the novelty of the manuscript, the authors have added text at the end of the Introduction and beginning of the Results sections which note that G4 quadruplex stabilizers such as RHPS4 and CX-5461 have not yet been successfully utilized in patients, so there is a need to test the capacity of other G4 stabilizers, such as pyridostatin. Further, the authors now do a better job at the end of the Introduction of stating the focus here on demonstrating the efficacy of pyridostatin against BRCA-deficient tumors, and also note novel findings that pyridostatin induces innate immune signaling in BRCA1/2-deficient cells and that it is effective against BRCA1-deficient tumors that are resistant to PARP inhibitors. Further, as suggested by this reviewer previously, there was a rearrangement of figures, attempting to make the novelty and importance of the current study more apparent by putting newer and/or impactful results in the main figures, with more confirmatory data as supplemental figures.

Despite these changes, in the opinion of this reviewer, the novelty and importance of this work is still not made sufficiently clear for readers. This is especially so for the non-specialist. Among the contributing factors:

1. It is not until well into the 6th page of the Results section, and only after 6 supplemental figures have been presented, that the first main figure is discussed. Perhaps not surprisingly, much of the Results section feels like there is no central focus. One alternative would be to put a main figure early in the Results section and use it as an anchor on which other main figures build. Supplemental Figures would then be there to support the story. Currently, there appears to be equal weight given to supplemental and main figures, making it more difficult to determine which findings are truly new.

2. The authors appropriately refer to their previous work (Zimmer et al., 2016) multiple times in the Results section as a foundation for discussing results that are shown in this manuscript. However, except for the 3rd paragraph on p. 12 (which begins "The clinical efficacy...") that supports Fig. 2A-B, in every other case Zimmer et al., 2016 is cited in the context of a supplemental figure. This contributes to the manuscript feeling incremental because of the repeated emphasis on confirmatory or direct extensions of previous results. For example, findings by Zimmer et al. that pyridostatin is toxic to, and induces DNA damage in, BRCA2-deficient cells are extended to BRCA2-deficient tumors in Figs. S1 and S2, respectively. Further, findings by

Zimmer et al. that pyridostatin slows DNA replication and inflicts DNA damage that activates the ATM/ATR dependent checkpoint, respectively, are extended to affects on replication fork protection (Fig. S3) and ATM kinase activation (Fig. S4). Additionally, work by Xu et al. 2017 is cited only once in the Results section, and Fig. S6 feels incremental by extending findings that DNA-PKcs deficient cells are hypersensitive to pyridostatin. Meanwhile, findings by Xu et al. that another G4 stabilizing agent, CX-5461, induces increased chromosome breakage in BRCA2-deficient cells, as well as DNA damage after a pulse, is ignored; thus, the presentation of similar results for the pyridostatin G4 stabilizer in Figs. S1, S2 and S3D-E also makes some of the supplemental figures feel incremental.

3. If the novel story presented in the main figures is the efficacy of pyridostatin combinations against BRCA1/2-deficient tumors, as well as induction of cGAS/STING innate immune signaling in BRCA1/2-deficient cells, perhaps part of the current Figure S1 (either A-B with DLD1-derived cells or C-D in HCT116-derived cells) should be part of the main figures as a foundation upon which many of the other main figures are built.

4. The order of presentation jumps back-and-forth between BRCA2 and BRCA1 deficiency in the Results section, somewhat interfering with the development of a cohesive story. For example the effect of pyridostatin on BRCA2 and BRCA1-deficient tumors are presented in Figs. S1 (beginning on p. 6) and 3 (p. 14), respectively. Similarly, effects of pyridostatin on ATM signaling after release from pyridostatin are presented in Fig. S4 (p. 9) and in Fig. 2C-D (p. 13) for BRCA2 and BRCA1 deficient cells, respectively. But for consideration of the effects of drug combinations, the order is reversed with BRCA1-deficient cells (Fig. 4, pp. 14-16) considered before BRCA2-deficient cells (Fig. 5, p. 16).

5. While Fig. 3 (p. 14 in the Results) demonstrates that pyridostatin targets BRCA1-deficient tumors that are resistant to PARPi, it is later disclosed in the Discussion that Xu et al., 2017 and Zimmer et al. 2016 already demonstrated that pyridostatin and CX-5461 "can counteract PARPi-resistance in BRCA1/2 deficient cells and tumours". It is the opinion of this reviewer that, as in other places, the manuscript needs to better clarify what is new vs what builds on previous results and/or is merely confirmatory.

Dear Lise,

thank you very much for the opportunity to revise our manuscript entitled “Anti-tumoral activity of the G-quadruplex ligand pyridostatin against BRCA1/2-deficient tumours” by Groelly, Porru *et al.* Firstly, we have changed the manuscript following suggestions made by Referee no.1. To do this, we included a new Figure 1 with selected relevant data from the Appendix, which now serves as the focal point for the start of our Results section, and which also shifts the balance towards main figures (as opposed to equal weight being given to main and supplementary figures, as the Reviewer commented). Secondly, we made all the editorial corrections requested in your email. The Paper Explained has also been corrected according to your suggestions.

We hope that with these revisions our manuscript meets the standards for publication in *EMBO Molecular Medicine.* Many thanks in advance for your consideration and we are looking forward to hearing from you.

With my best wishes,

Madalena Tarsounas, PhD
Professor of Molecular and Cell Biology
University of Oxford, Department of Oncology
CRUK/MRC Oxford Institute for
Radiation Biology, Oxford, UK
madalena.tarsounas@oncology.ox.ac.uk

7th Jan 2022

Dear Madalena,

Thank you for sending the revised files. Everything is now fine, and I am therefore very pleased to inform you that your manuscript is accepted for publication in EMBO Molecular Medicine!
It will now be sent to our publisher to be included in the next available issue of EMBO Molecular Medicine.

Please read below for additional IMPORTANT information regarding your article, its publication and the production process.

Congratulations on your interesting work, and my best wishes for the new year!

Lise

Lise Roth, Ph.D
Editor
EMBO Molecular Medicine

Follow us on Twitter @EmboMolMed
Sign up for eTOCs at embopress.org/alertsfeeds

EMBO PRESS

YOU MUST COMPLETE ALL CELLS WITH A PINK BACKGROUND ➔

PLEASE NOTE THAT THIS CHECKLIST WILL BE PUBLISHED ALONGSIDE YOUR PAPER

Corresponding Author Name: Madalena Tarsounas and Annamaria Biroccio
Journal Submitted to: EMBO Molecular Medicine
Manuscript Number: EMM-2021-14501

Reporting Checklist For Life Sciences Articles (Rev. June 2017)

This checklist is used to ensure good reporting standards and to improve the reproducibility of published results. These guidelines are consistent with the Principles and Guidelines for Reporting Preclinical Research issued by the NIH in 2014. Please follow the journal's authorship guidelines in preparing your manuscript.

A- Figures
1. Data
The data shown in figures should satisfy the following conditions:

- the data were obtained and processed according to the field's best practice and are presented to reflect the results of the experiments in an accurate and unbiased manner.
- figure panels include only data points, measurements or observations that can be compared to each other in a scientifically meaningful way.
- graphs include clearly labeled error bars for independent experiments and sample sizes. Unless justified, error bars should not be shown for technical replicates.
- if n< 5, the individual data points from each experiment should be plotted and any statistical test employed should be justified
- Source Data should be included to report the data underlying graphs. Please follow the guidelines set out in the author ship guidelines on Data Presentation.

2. Captions

Each figure caption should contain the following information, for each panel where they are relevant:

- a specification of the experimental system investigated (eg cell line, species name).
- the assay(s) and method(s) used to carry out the reported observations and measurements
- an explicit mention of the biological and chemical entity(ies) that are being measured.
- an explicit mention of the biological and chemical entity(ies) that are altered/varied/perturbed in a controlled manner.

- the exact sample size (n) for each experimental group/condition, given as a number, not a range;
- a description of the sample collection allowing the reader to understand whether the samples represent technical or biological replicates (including how many animals, litters, cultures, etc.).
- a statement of how many times the experiment shown was independently replicated in the laboratory.
- definitions of statistical methods and measures:
  - common tests, such as t-test (please specify whether paired vs. unpaired), simple χ2 tests, Wilcoxon and Mann-Whitney tests, can be unambiguously identified by name only, but more complex techniques should be described in the methods section;
  - are tests one-sided or two-sided?
  - are there adjustments for multiple comparisons?
  - exact statistical test results, e.g., P values = x but not P values < x;
  - definition of 'center values' as median or average;
  - definition of error bars as s.d. or s.e.m.

Any descriptions too long for the figure legend should be included in the methods section and/or with the source data.

In the pink boxes below, please ensure that the answers to the following questions are reported in the manuscript itself. Every question should be answered. If the question is not relevant to your research, please write NA (non applicable). We encourage you to include a specific subsection in the methods section for statistics, reagents, animal models and human subjects.

USEFUL LINKS FOR COMPLETING THIS FORM

http://www.antibodypedia.com
http://1degreebio.org
http://www.equator-network.org/reporting-guidelines/improving-bioscience-research-repor
http://grants.nih.gov/grants/olaw/olaw.htm
http://www.mrc.ac.uk/Ourresearch/Ethicsresearchguidance/Useofanimals/index.htm
http://ClinicalTrials.gov
http://www.consort-statement.org
http://www.consort-statement.org/checklists/view/32-consort/66-title
http://www.equator-network.org/reporting-guidelines/reporting-recommendations-for-tumo
http://datadryad.org
http://figshare.com
http://www.ncbi.nlm.nih.gov/gap
http://www.ebi.ac.uk/ega
http://biomodels.net/
http://biomodels.net/miriam/
http://jjj.biochem.sun.ac.za
https://osp.od.nih.gov/biosafety-biosecurity-and-emerging-biotechnology/
http://www.selectagents.gov/

B- Statistics and general methods

Please fill out these boxes ➔ (Do not worry if you cannot see all your text once you press return)

1.a. How was the sample size chosen to ensure adequate power to detect a pre-specified effect size?	Cell line experiments were performed at least three times (unless otherwise stated), with technical triplicates for viability or survival assays. Results show the average of three independent repeats (unless otherwise stated) and SEM bars for the three repeats are shown for every datapoint.
1.b. For animal studies, include a statement about sample size estimate even if no statistical methods were used.	The use of five mice per experimental group is acceptable practice, in line with national and international guidelines for Laboratory Animal Care and as in previous publications (Zimmer et al., Mol Cell 2016).
2. Describe inclusion/exclusion criteria if samples or animals were excluded from the analysis. Were the criteria pre-established?	N/A
3. Were any steps taken to minimize the effects of subjective bias when allocating animals/samples to treatment (e.g. randomization procedure)? If yes, please describe.	N/A
For animal studies, include a statement about randomization even if no randomization was used.	Animal studies were randomized.
4.a. Were any steps taken to minimize the effects of subjective bias during group allocation or/and when assessing results (e.g. blinding of the investigator)? If yes please describe.	Investigators were blinded when quantifying microscopy and immunofluoresence images.
4.b. For animal studies, include a statement about blinding even if no blinding was done	All animal analysis was performed blinded to experimental group assignment.
5. For every figure, are statistical tests justified as appropriate?	Yes.
Do the data meet the assumptions of the tests (e.g., normal distribution)? Describe any methods used to assess it.	Yes. The software Graphpad Prism was used to assess data sets.

Is there an estimate of variation within each group of data?	Yes.
Is the variance similar between the groups that are being statistically compared?	Yes.

C- Reagents

6. To show that antibodies were profiled for use in the system under study (assay and species), provide a citation, catalog number and/or clone number, supplementary information or reference to an antibody validation profile. e.g., Antibodypedia (see link list at top right), 1DegreeBio (see link list at top right).	The antibodies used in the study are extensively used in scientific literature and consistent results can be found in previous publications of the group (Zimmer et al. Mol Cell 2016; Tacconi et al. EMBO Mol Med 2017 and 2019; Reisländer et al. Nature Commun 2020). All antibodies catalog numbers and dilutions are provided in the Materials and Methods section of the manuscript.
7. Identify the source of cell lines and report if they were recently authenticated (e.g., by STR profiling) and tested for mycoplasma contamination.	All sources of cell lines and/or references are stated in the Materials and Methods section of the manuscript. All cell lines are routinely tested for mycoplasma contamination and authenticated.

* for all hyperlinks, please see the table at the top right of the document

D- Animal Models

8. Report species, strain, gender, age of animals and genetic modification status where applicable. Please detail housing and husbandry conditions and the source of animals.	The information is included in Materials and Methods.
9. For experiments involving live vertebrates, include a statement of compliance with ethical regulations and identify the committee(s) approving the experiments.	The information is included in Materials and Methods.
10. We recommend consulting the ARRIVE guidelines (see link list at top right) (PLoS Biol. 8(6), e1000412, 2010) to ensure that other relevant aspects of animal studies are adequately reported. See author guidelines, under 'Reporting Guidelines'. See also: NIH (see link list at top right) and MRC (see link list at top right) recommendations. Please confirm compliance.	We confirm compliance.

E- Human Subjects

11. Identify the committee(s) approving the study protocol.	Collection of fresh tumour samples from patients for implantation into mice was done under an institutional IRB-approved protocol or by the National Research Ethics Service, Cambridgeshire 2 REC (REC reference number: 08/H0308/178) (Bruna et al., 2016).
12. Include a statement confirming that informed consent was obtained from all subjects and that the experiments conformed to the principles set out in the WMA Declaration of Helsinki and the Department of Health and Human Services Belmont Report.	Written informed consent was obtained from all patients and the experiments conformed to the principles set out in the WMA Declaration of Helsinki and the Department of Health and Human Services Belmont Report.
13. For publication of patient photos, include a statement confirming that consent to publish was obtained.	N/A
14. Report any restrictions on the availability (and/or on the use) of human data or samples.	N/A
15. Report the clinical trial registration number (at ClinicalTrials.gov or equivalent), where applicable.	N/A
16. For phase II and III randomized controlled trials, please refer to the CONSORT flow diagram (see link list at top right) and submit the CONSORT checklist (see link list at top right) with your submission. See author guidelines, under 'Reporting Guidelines'. Please confirm you have submitted this list.	N/A
17. For tumor marker prognostic studies, we recommend that you follow the REMARK reporting guidelines (see link list at top right). See author guidelines, under 'Reporting Guidelines'. Please confirm you have followed these guidelines.	N/A

F- Data Accessibility

18: Provide a "Data Availability" section at the end of the Materials & Methods, listing the accession codes for data generated in this study and deposited in a public database (e.g. RNA-Seq data: Gene Expression Omnibus GSE39462, Proteomics data: PRIDE PXD000208 etc.) Please refer to our author guidelines for 'Data Deposition'.<br><br>Data deposition in a public repository is mandatory for:<br>a. Protein, DNA and RNA sequences<br>b. Macromolecular structures<br>c. Crystallographic data for small molecules<br>d. Functional genomics data<br>e. Proteomics and molecular interactions	N/A
19. Deposition is strongly recommended for any datasets that are central and integral to the study; please consider the journal's data policy. If no structured public repository exists for a given data type, we encourage the provision of datasets in the manuscript as a Supplementary Document (see author guidelines under 'Expanded View' or in unstructured repositories such as Dryad (see link list at top right) or Figshare (see link list at top right).	N/A
20. Access to human clinical and genomic datasets should be provided with as few restrictions as possible while respecting ethical obligations to the patients and relevant medical and legal issues. If practically possible and compatible with the individual consent agreement used in the study, such data should be deposited in one of the major public access-controlled repositories such as dbGAP (see link list at top right) or EGA (see link list at top right).	N/A
21. Computational models that are central and integral to a study should be shared without restrictions and provided in a machine-readable form. The relevant accession numbers or links should be provided. When possible, standardized format (SBML, CellML) should be used instead of scripts (e.g. MATLAB). Authors are strongly encouraged to follow the MIRIAM guidelines (see link list at top right) and deposit their model in a public database such as Biomodels (see link list at top right) or JWS Online (see link list at top right). If computer source code is provided with the paper, it should be deposited in a public repository or included in supplementary information.	N/A

G- Dual use research of concern

22. Could your study fall under dual use research restrictions? Please check biosecurity documents (see link list at top right) and list of select agents and toxins (APHIS/CDC) (see link list at top right). According to our biosecurity guidelines, provide a statement only if it could.	N/A
